# Loss of full-length DAZL isoform disrupts PABPC1-dependent translational regulation and meiosis

**DOI:** 10.1038/s41419-025-08179-7

**Published:** 2025-11-17

**Authors:** Xin Li, Dandan Cao, Yue Lu, Zexin Bian, Tingting Zhao, Wenbo Liu, Yan Zhao, Jun Tan, Lei Li, Eugene Yujun Xu

**Affiliations:** 1 Reproductive Medicine Center, JXHC Key Laboratory of Fertility Preservation, Jiangxi Maternal and Child Health Hospital, Nanchang Medical College, Nanchang, Jiangxi Province China; 2 State Key Laboratory of Reproductive Medicine and Offspring Health, Nanjing Medical University, Nanjing, Jiangsu China; 3 State Key Laboratory of Medical Genomics, Research Center for Experimental Medicine, Ruijin Hospital, Shanghai Jiao Tong University School of Medicine, Shanghai, China; 4 Department of Cardiology, Zhongda Hospital, School of Medicine, Southeast University, Nanjing, Jiangsu China; 5 State Key Laboratory of Reproductive Medicine and Offspring Health, Center for Reproductive Medicine, Institute of Women, Children and Reproductive Health, Shandong University, Jinan, Shandong China; 6 National Research Center for Assisted Reproductive Technology and Reproductive Genetics, Shandong University, Jinan, Shandong China; 7 Key Laboratory of Organ Regeneration and Reconstruction, Beijing Institute of Stem Cell and Regenerative Medicine, Institute of Zoology, Chinese Academy of Sciences, Beijing, China; 8 Cellular Screening Center, The University of Chicago, Chicago, IL USA

**Keywords:** Spermatogenesis, Infertility, Ribosome

## Abstract

Alternative splicing (AS) events are exceptionally active during spermatogenesis, enhancing the diversity of the testicular transcriptome and proteome. In mouse testes, the germ cell-specific RNA-binding protein DAZL undergoes alternative splicing to produce two isoforms: a full-length DAZL (DAZL_FL) and a short isoform lacking exon 8 (DAZL_Sh). While DAZL is a hallmark of germ cell development, the physiological roles of its alternative splicing, and the distinct functions of these two isoforms remain yet to be fully elucidated. To investigate this, we disrupted alternative splicing of *Dazl* by generating DAZL short isoform only mice via deletion of the DAZL exon 8 and found that the resulting loss of DAZL_FL led to male infertility, characterized by extensive spermatocyte apoptosis and arrest of spermatid development. Ribosome profiling (Ribo-seq) revealed that loss of DAZL_FL downregulated the translation of meiotic genes specifically bound by DAZL. Mechanistically, DAZL_Sh failed to interact with Poly(A) Binding Protein Cytoplasmic 1 (PABPC1), resulting in impaired translation of DAZL-targeted germ cell transcripts. In females, DAZL_FL ablation caused complete infertility, marked by massive primordial follicle apoptosis and failure of primary follicle recruitment, highlighting a shared role for DAZL_FL in meiotic regulation in both sexes. Our findings established DAZL/PABPC1 complex formation as a pivotal mechanism controlling meiotic progression. By functionally dissecting DAZL isoforms, we uncovered a critical role of *Dazl* alternative splicing during spermatogenesis and folliculogenesis, further expanding the roles of DAZL in germ cell development and thereby providing novel genetic causes and potential therapeutic targets for azoospermia and primary ovarian insufficiency.

## Introduction

Alternative splicing (AS) serves as a pivotal regulatory mechanism in gene expression, significantly expanding the functional complexity and diversity of the proteome [1]. Specifically, the 21,144 multi-exonic protein-coding genes in the human genome can generate over 210,000 distinct protein isoforms, with an average of 3.4 isoforms per gene [1, 2]. Comparative transcriptome analyses have revealed that the brain and testis exhibit the highest prevalence of alternative splicing events [3–6]. Spermatogenesis is a highly coordinated and complex process that requires precise spatiotemporal control of gene expression in different spermatogenic cells. Abnormal pre-mRNA alternative splicing affects mRNA stability, translation, and localization [7]. Animal knockout models of numerous genes involved in alternative splicing, including *Bcas2*, *Alkbh5*, *Cwf19l2*, *Ptbp2*, *Srsf10*, and *Mettl3*, have demonstrated that aberrant splicing leads to severe defects in spermatogenesis [2, 8–13]. Additionally, BCAS2, SRSF1, and DSN1 play critical roles in granulosa cell development, oocyte maturation, and early embryonic development through the regulation of alternative splicing [14–16]. Elucidating the roles of specific splicing variants in germ cell development is crucial for uncovering the molecular mechanisms underlying reproductive biology.

The *DAZ* (*Deleted in Azoospermia*) gene family, which includes *DAZ*, *DAZL* (*DAZ-like*), and *BOULE*, is highly conserved across species and is essential for germ cell development and fertility [17–20]. Among these, DAZL is particularly crucial, as it is required throughout germ cell development from embryonic development to postnatal gametogenesis in both male and female. Loss of DAZL results in a complete arrest of germ cell development, making DAZL a crucial regulator of both embryonic germ cell development and postnatal gametogenesis [21–24]. Mechanistically, DAZL binds to the 3’ untranslated regions (3’ UTRs) of target mRNAs to regulate spermatogonial proliferation and differentiation, synaptonemal complex assembly during meiosis, and spermiogenesis [24–26].

Furthermore, DAZL post-transcriptionally enhances the expression of target mRNAs, promoting oocyte maturation [27, 28]. While DAZL has been shown to be a critical regulator in embryonic germ cell development, likely through a similar posttranscriptional mechanism [25, 29–31], the exact molecular mechanism remains largely unknown. Recent report uncovered a novel mode of DAZL function in promoting the proliferation of human primordial germ cells and suppressing the pluripotency program by binding and facilitating miRNA maturation (e.g., let-7 family) [32]. Additionally, the germ cell-specific DAZL, when mutated, could lead to germ cell tumor or could be activated in somatic tumors, like non-small cell lung cancer development and cisplatin resistance through distinct molecular pathways [33]. In mice, *Dazl* undergoes alternative splicing, generating two predominant transcript variants: the full-length isoform (DAZL_FL) and a shorter isoform (DAZL_Sh) that lacks exon 8 [34]. While both isoforms are expressed in wild type mice, the precise molecular mechanisms and functional roles of each isoform in germ cell development remain largely unknown.

DAZL is a well-established posttranscriptional regulator playing a crucial role during gametogenesis. Recent studies have emphasized the important role of DAZL in translational regulation by binding to the 3’ UTRs of target mRNAs, especially through its interaction with PABPC1 [24–27, 35]. These interactions are thought to be crucial for the stabilization and translation of target mRNAs involved in meiosis and germ cell differentiation during spermatogenesis. Moreover, DAZL and CPEB1 cooperatively regulate the translation of maternal mRNAs during mouse oocyte maturation [28, 36]. Based on these findings, it is suggested that the combinatorial action of DAZL with different partner proteins in distinct cellular contexts highlights its versatile role in posttranscriptional regulation throughout gametogenesis.

This study demonstrated that DAZL_FL was indispensable for spermatogenesis and folliculogenesis. In male mice, the absence of DAZL_FL resulted in a spermatogenic arrest at the elongating spermatid stage, accompanied by extensive apoptosis of spermatocytes. Mechanistically, we demonstrated that the loss of the DAZL_FL isoform disrupted the interaction between DAZL and PABPC1, resulting in the translational downregulation of key meiotic genes. Similarly, DAZL_FL deficiency resulted in the arrest of folliculogenesis at the primordial follicle stage in female mice, further emphasizing the importance of DAZL in meiotic progression. This study provides new insights into the molecular mechanisms by which DAZL regulates gametogenesis and underscores the importance of alternative splicing in fine-tuning gene function of key germ cell regulators during reproductive development.

## Materials and methods

### Animals

The heterozygous *Dazl*^*E8KO*^ mice were constructed by targeting the intronic region upstream and downstream of exon 8 using CRISPR-Cas9 technology. The Dazl^+/-^ mice were derived from offspring of our lab′s Dazl conditional knockout line [24]. Intercrossing these heterozygotes produced homozygous Dazl^-/-^ mice for this study. The mouse lines were maintained in the C57BL/6J background. The animals were housed in the SPF (Specific-Pathogen-Free) animal facility of Nanjing Medical University with standard 12-h light/dark cycles, and standard humidity and temperature, where they had unrestricted access to both water and food. Genotyping of *Dazl*^*E8KO*^ was performed by PCR using genomic DNA extracted from mouse toes. Forward primer (F1, 5-TGTGATGGATGC TGTGA AATAG-3) and a reverse primer (R1, 5-CACTGCGGTGGCATCTTAA-3) were used to detect the wildtype allele, and Forward primer F1 and reverse primer R2 (5-CAAAATATCAGCTCCTGGATCAA CT-3) were used to detect the mutant allele in PCR genotyping.

### Histological analysis, chromosome spread, TUNEL assay, and immunostaining

Testes from wildtype and *Dazl*^*E8KO*^ male mice were isolated and fixed in Hartman′s fixative (H0290, Sigma) for 48 h. All samples were dehydrated stepwise through a gradient concentration of ethanol (70%, 80%, 90%, 100%), subsequently embedded in paraffin (product number: 39601006, Leica) and sectioned at a thickness of 5 μm. The sections were dewaxed in a 56 °C oven for 4 h and subjected to hydration, subsequently stained with hematoxylin and 1% eosin, followed by clearing with xylene and then mounted with resin. Histological analysis was performed with a Nikon ECLIPSE TI2 microscope. To perform chromosome spread analysis, we collected germ cells from the testes of P35 wildtype and *Dazl*^*E8KO*^ mice using a previously established method [24, 37]. TUNEL assays were carried out as previously described [38], and the number of TUNEL-positive cells was counted in at least three randomly selected, non-contiguous testicular sections of different ages from both WT and *Dazl*^*E8KO*^ mice.

For immunostaining, following dewaxing and hydration, the testicular sections or embryonic ovarian sections were subjected to antigen retrieval through boiling in EDTA Antigen Retrieval Solution (P0085, Beyotime) for 15 min in a microwave oven. Following three washes with PBS, the sections were blocked with 5% BSA for 90 min at room temperature and then incubated with primary antibodies overnight at 4 °C. The primary antibodies were as follows: Mouse anti-γH2A.X monoclonal antibody (sc-517348, Santa Cruz, 1:500); Mouse anti-SYCP3 monoclonal antibody (ab181746, Abcam, 1:200)；Rabbit anti-DDX4 monoclonal antibody (ab270534, Abcam, 1:200); and Rat anti-TRA98 monoclonal antibody (ab82527, Abcam, 1:200). The following day, the sections were washed three times with PBS, then incubated with secondary antibodies for 1 h at room temperature. Afterwards, they were washed three more times with PBS and mounted with DAPI (D9542, Sigma). The immunofluorescence staining was imaged with a Laser Scanning Confocal Microscope (LSM800, Carl Zeiss).

### RNA extraction and RT-qPCR

Total RNA from testes samples of WT and *Dazl*^*E8KO*^ mice was extracted with TRIzol reagent (15596026CN, Invitrogen). Single-stranded cDNAs were generated from 500 ng of total RNA with the cDNA synthesis kit Prime Script RT Master Mix (RR036Q, Takara). Diluted cDNAs were used for each reaction using SYBR Green Master Mix (Q141-02, Vazyme). A standard 20 μl reaction volume contained forward and reverse primers, 1 μl of cDNA, and 10 μl of SYBR Green Mix. Primer sequences are shown in Supplemental Table I. *Actb* and *Rpl22* were used as controls for normalization.

### Western blotting

Cell lysates obtained from mouse testes were homogenized using glass homogenizers in RIPA lysis buffer (P10013B, Beyotime), which was supplemented with an EDTA-free protease inhibitor cocktail (04693132001, Roche). The lysates were centrifuged at 20,000 × *g* for 20 min at 4 °C, and the supernatants were used for western blotting analyses by following standard procedures. Equal quantities of total protein were separated on 10% or 15% SDS-PAGE gels and subsequently transferred onto PVDF membranes (IPFL00010, Millipore). The membranes were blocked with 5% skim milk for 1 h at room temperature, followed by three washes with TBST, and then incubated with the primary antibodies at 4 °C overnight. The primary antibodies used were as follows: mouse anti-DAZL antibody (ABD Serotec, MCA2336, 1:1000); rabbit anti-PABPC1 antibody (Abcam, ab21060, 1:1000); mouse anti-GAPDH antibody (Abcam, ab8245, 1:3000); mouse anti-ACTB antibody (Abcam, ab8224, 1:3000); mouse anti-EIF4G3 antibody (Sigma, SAB1411985, 1:1000); rabbit anti-HSPA2 antibody (Abcam, ab108416, 1:1000); rabbit anti-CDK2 antibody (CST, 18048, 1:1,000); rabbit anti-TEX19.1 antibody (Abmart, PC13612, 1:1000); rabbit anti-MDC1 antibody (Abcam, ab271061, 1:1000); rabbit anti-PSMA8 antibody (Proteintech, 14022-1-AP, 1:1000); rabbit anti-HORMAD1 antibody (Abcam, ab307424, 1:1000); rabbit anti-MEIOC antibody (Abmart, PS13237, 1:1000); rabbit anti-MLH3 antibody (Abmart, TP73001, 1:1000). Following three washes with TBST, the membranes were then incubated with HRP-conjugated secondary antibodies for 1 h at room temperature. The signals were developed with ECL Chemiluminescence Kit (E411, Vazyme), and finally detected with Bio-Rad ChemiDoc XRS (Bio-Rad Laboratories)

### RNA immunoprecipitation

Testes were homogenized on ice using a glass homogenizer in lysis buffer (50 mM pH 7.4 Tris, 100 mM KCl, 12 mM MgCl_2_, 1% NP-40, 1 mM DTT, 100 μg/mL Cyclohexamide, 1 mg/mL Heparin) supplemented with protease inhibitor (04693132001, Roche) and RNase inhibitor (N2515, Promega). The homogenized testes were centrifuged at 4 °C for 15 min at 20,000 × *g*, and then the supernatants were rotated at 4 °C for 20 min. We added 10 μg antibody or IgG to each lysate sample and incubated for 4 h on ice. The supernatant was immunoprecipitated with rinsed Dynabeads (Invitrogen, 10004D) at 4 °C for at least 8 h. Next, the magnetic beads were washed four times with wash buffer (50 mM pH 7.4 Tris, 100 mM KCl, 12 mM MgCl_2_, 1% NP-40) and an additional three times with high salt buffer (50 mM Tris, 300 mM KCl, 12 mM MgCl_2_, 1% NP-40, 1 mM DTT). The bead-antibody-protein complexes were resuspended in 50 μl of wash buffer and then digested with DNase at 37 °C for 20 min and 30 μg of proteinase K (A610451, Sangon Biotech) at 56 °C for 20 min. Finally, total RNAs were extracted with TRIzol reagent (15596026CN, Invitrogen).

### Dual luciferase reporter assay

For the construction of reporter plasmids, the 3’ UTRs of *Tex19.1*, *Psma8* or *Mdc1* were amplified by PCR and then subcloned into the psiCHECK-2 vector (Promega). For the construction of effector plasmids, mouse *Dazl_FL or Dazl_Sh* cDNA or mouse *Pabpc1* cDNA was subcloned into the pCMV6 plasmid (OriGene). NIH3T3 cells in 24-well plates were co-transfected with 150 ng of the psiCHECK2 reporter plasmid and 450 ng of the effector plasmid using FuGENE HD transfection reagent (E2311, Promega) according to the manufacturer’s protocol. After 48 h, Firefly and Renilla luciferase activities were measured using the Dual Luciferase Reporter Assay System (E1910, Promega) according to the manufacturer’s instructions.

### Ribo-seq

The testes obtained from adult WT and *Dazl*^*E8KO*^ mice were homogenized in a homogenizer and then subjected to sonication in a Bioruptor. Following lysis on ice for 15 min, the lysate was centrifuged at 20,000 × *g* for 15 min at 4 °C. To prepare ribosome footprints (RFs), 10 μl RNase I and 10 μl DNase I were added to each supernatant and incubated for 45 min at room temperature. The digested RFs were loaded onto the pre-equilibrated columns and centrifuged. A volume of 10 μL of 10% SDS was added to the elution, and subsequently, RFs larger than 17 nt in size were isolated using the RNA Clean and Concentrator-25 kit (R1017, Zymo Research). After rRNA removal, RFs were further purified using magnetic beads. To construct Ribo-seq libraries, the RFs were then appended with adapters at both ends, followed by reverse transcription and PCR amplification. Finally, the cDNA libraries were sequenced using an Illumina HiSeq X10 sequencer provided by Novogene (Beijing, China).

### Statistical analysis

Statistical analyses were performed using GraphPad Prism 9 (GraphPad Software, San Diego, CA). Data are presented as mean ± standard deviation (SD) from at least three independent biological replicates (*n* ≥ 3), unless otherwise stated, For comparisons between two groups, two-tailed unpaired Student’s t-tests were used to calculate *P* values. Statistical significance was defined as *P* < 0.05. Exact *P* values are reported in the figures for all comparisons, whether significant or non-significant (ns).

## Results

### The expression patterns of DAZL isoforms during spermatogenesis

**Fig. 1:**
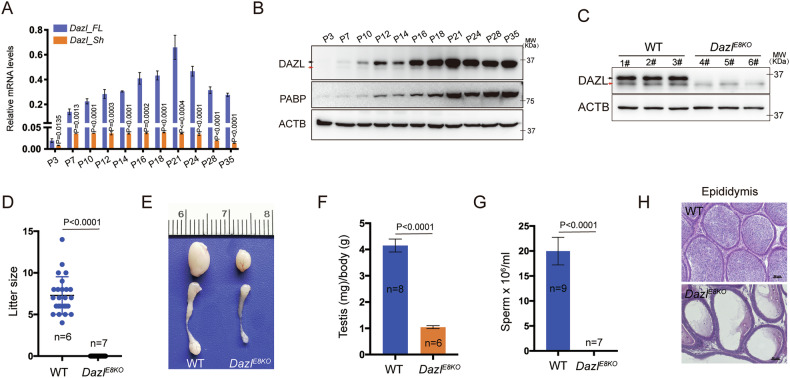
Loss of DAZL_FL isoform results in male infertility. **A** RT-qPCR results demonstrating the expression of full-length (*Dazl_FL*) and short (*Dazl_Sh*) splicing variants of *Dazl* in postnatal testes from postnatal day 3(P3) to day 35(P35). Data are represented as the mean ± SD, with *P* values indicated. **B** Western blot analysis of DAZL_FL and DAZL_Sh isoforms, as well as PABPC1 protein, in the testes of wildtype mice at different postnatal ages. The black and red arrows indicate the DAZL_FL and DAZL_Sh isoforms, respectively. ACTB protein was used as a loading control. **C** Verification of the DAZL_FL knockout in the 35-day-old testes of *Dazl*^*E8KO*^ mice. ACTB protein was employed as a loading control. The black arrow indicates DAZL_FL, and the red arrow indicates DAZL_Sh. **D** Histogram shows the litter size of adult wildtype (WT, *n* = 6) and *Dazl*^*E8KO*^ (*n* = 7) male mice. Data are presented as mean ± SD. **E** Representative images of testes and epididymis from adult *Dazl*^*E8KO*^ and wildtype littermates. **F** Quantitation of the testis/body weight ratio for adult *Dazl*^*E8KO*^ (*n* = 6) and wildtype littermates (*n* = 8). **G** Quantitative comparison of epididymal sperm concentration between adult wildtype and *Dazl*^*E8KO*^ littermates. Data bars are mean ± SD. **H** Hematoxylin and eosin-stained cross-sections from the cauda of epididymis from adult wildtype and *Dazl*^*E8KO*^ littermates. Bars are 50 μm.

To characterize the roles of different isoforms of DAZL during spermatogenesis, we first examined the expression of *Dazl_FL* and *Dazl_Sh* transcripts during the first wave of spermatogenesis, using mouse testes collected at 11 time points ranging from postnatal day 3 (P3) to postnatal day 35 (P35). The first wave of mouse spermatogenesis is synchronized and the different stages of spermatogenic cells will appear sequentially during the postnatal 35 days, allowing us to determine the developmental express profile of DAZL isoforms using RT-qPCR and Western blot analyses of the neonatal testes. Our results indicated that the *Dazl_FL* transcript gradually increased in the testes after birth, reaching a peak at postnatal day 21, whereas the expression of the short transcript remained relatively stable after P7 (Fig. 1A). The protein expression profiles of DAZL isoforms showed consistent co-expression throughout spermatogenesis, with DAZL-FL being the predominant isoform. The expression of DAZL increased upon meiosis entry (P10) compared to the spermatogonia stage (P7) (Fig. 1B). When spermatogenesis progressed to the mid-pachytene stage (P16), the expression level of DAZL protein became higher and remained high until postnatal day 35 (Fig. 1B). However, the expression of the DAZL_Sh subtype was significantly lower than that of DAZL_FL, which was consistent with the expression of mRNA (Fig. 1B). These results suggested that DAZL_FL might play a significant role during spermatogenesis. Interestingly, PABPC1, a partner protein of DAZL, was also detected during the first wave of spermatogenesis, and exhibited a similar increase at postnatal day 12 and postnatal day 21, supporting the interaction between these two proteins during spermatogenesis (Fig. 1B) [24, 35]

### Loss of DAZL_FL protein resulted in male infertility

In order to evaluate the physiological requirement of DAZL alternative splicing, in particular the predominant full-length isoform during gametogenesis, we generated a *Dazl* exon 8 knockout mice on a C57BL/6 background using CRISPR-Cas9 method (Fig. S1A, B). Heterozygous mice displayed no developmental abnormalities and were fertile, allowing us to generate homozygous offspring. Quantitative real-time PCR (RT-qPCR) and Western blotting analyses of the 35-day-old testes from WT and *Dazl*^*E8KO*^ mice revealed complete loss of mRNA and protein expression of the DAZL_FL subtype (Fig. S1B and Fig. 1C). Notably, we found that the transcript level of *Dazl_Sh* was elevated in *Dazl*^*E8KO*^ testes compared to that of wildtype, while its protein level was significantly reduced (Figs. 1C, S1C, D). This suggests that DAZL_FL deletion may affect DAZL_Sh expression at the posttranscriptional level. While the loss of the DAZL_FL isoform does not impair reproductive behavior in mice (Fig. S1E), extended mating trials over six months failed to produce offspring (Fig. 1D). Additionally, *Dazl*^*E8KO*^ mice exhibited significantly smaller testes compared to those of their littermate controls (Fig. 1E, F). Mature sperm were absent in the cauda epididymis of *Dazl*^*E8KO*^ mice, as confirmed by sperm count (Fig. 1G) and histology (Fig. 1H). Collectively, our results demonstrate that the DAZL_FL isoform is required for spermatogenesis and fertility.

### The absence of DAZL_FL led to delayed postnatal germline development, increased apoptosis of pachytene spermatocytes, and spermiogenesis arrest, but did not affect the maintenance and differentiation of spermatogonia

Previous reports have shown that DAZL protein serves as a gateway for germ cell specification and migration, and loss of DAZL results in the absence of germ cells in both ovaries and testes after birth in C57BL/6 mouse strain [30, 31]. We detected spermatogonia in the testes of *Dazl*^*E8KO*^ mice on P7, which were very similar to those in wildtype mice (Fig. S2A). In contrast, there were completely no germ cells in the testes of *Dazl*^*-/-*^ (knockout of both DAZL isoforms) mice on P7 (Fig. S2A), indicating that the DAZL_Sh isoform is capable of supporting the differentiation of primordial germ cells (PGCs) into spermatogonia. We utilized PLZF [39], a marker protein for undifferentiated spermatogonial stem cells, to label these cells through immunohistochemical staining. By analyzing testicular sections of wildtype and *Dazl*^*E8KO*^ mice at 1, 2, 3, 4, 5, 10, and 25 weeks, we found that the DAZL_Sh alone was sufficient to sustain both the maintenance and differentiation of spermatogonial stem cells (Fig. S2B, C). This was also validated by another marker of spermatogonial stem cells, LIN28A [40] (Fig. S2D, E). Hence, the DAZL_FL protein is not essential for embryonic germ cell development and maintenance and differentiation of spermatogonial stem cells during postnatal spermatogenic development.

**Fig. 2:**
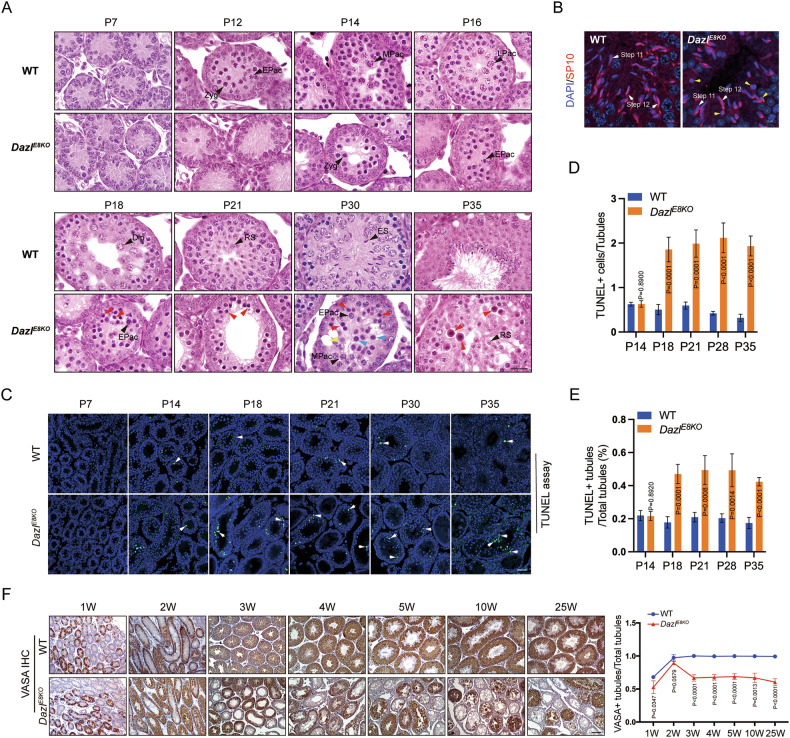
Extensive apoptosis of pachytene spermatocytes and arrested spermiogenesis in *Dazl*^*E8KO*^ male mice. **A** Hematoxylin and eosin stain of wildtype and *Dazl*^*E8KO*^ testes at P7, P12, P14, P16, P18, P21, P30 and P35. P, postnatal. Zyg zygotene, EPac early pachytene, MPac middle pachytene, LPac late pachytene, Dip diplotene, RS round spermatid, ES elongated spermatid. Red arrows mark apoptotic cells; The yellow arrows indicate the vacuoles resulting from the apoptosis of germ cells; The blue arrows mark the spermatocytes undergoing apoptosis. The scale bar is 50 μm. **B** Paraffin wax-embedded sections prepared from the testes of 8-week *Dazl*^*E8KO*^ and their wildtype littermates were immunolabeled with SP10 antibody (red) and DAPI (blue). Yellow arrows indicate apoptotic germ cells. Scale bar: 10 μm. **C** TUNEL staining of testicular sections from wildtype and *Dazl*^*E8KO*^ mice at P7, P14, P18, P21, P30, P35. Chromatin was stained with DAPI (blue). P, postnatal. White arrows indicate apoptotic germ cells. Scale bar: 50 μm. **D** The quantification of TUNEL-positive cells in each seminiferous tubule of testes from *Dazl*^*E8KO*^ and wildtype mice. **E** The percentage of TUNEL-positive seminiferous tubules from *Dazl*^*E8KO*^ and wildtype mice. *N* = 3 for each time point in each genotype. Data are presented as mean ± SD, with *P* values indicated. **F** Immunostaining of germ cells in testis sections at various ages using an antibody against VASA, and quantification of the percentage of VASA-positive tubules in *Dazl*^*E8KO*^ and wildtype mice. Data presented are mean ± SD, *P* values are annotated in the line chart.

To further investigate the impact of DAZL_FL deletion on spermatogenesis, we conducted histological analysis on the mutant mice during the first wave of spermatogenesis. There were no significant differences in the cellular composition of the seminiferous tubules in the testes between wildtype and *Dazl*^*E8KO*^ mice on P7 (Fig. 2A). In the wildtype seminiferous tubules at postnatal day 12, many zygotene spermatocytes and a small number of early pachytene spermatocytes were observed (Fig. 2A). However, zygotene spermatocytes first appeared in the tubules of *Dazl*^*E8KO*^ mice on postnatal day 14, by which time mid-pachytene spermatocytes were already present in wildtype tubules (Fig. 2A). When late-pachytene spermatocytes emerged in wildtype tubules at postnatal day 16, the germ cells in the tubules of *Dazl*^*E8KO*^ mice had not advanced beyond early spermatocytes (Fig. 2A). These results indicate that deletion of the DAZL_FL leads to a delay in spermatocyte development.

By P18, the most advanced spermatogenic cells have transitioned through pachytene and progressed to the diplotene stage (Fig. 2A). Meanwhile, the germ cells within the tubules of *Dazl*^*E8KO*^ mice exhibited a large number of abnormally condensed nuclei and apoptotic cells (Fig. 2A). On postnatal days 21, 30, and 35, round spermatids, elongated spermatids, and mature spermatozoa could be observed within the tubules of wildtype mice, respectively (Fig. 2A). In contrast, only a very small number of round spermatids were detected in the tubules of P35 *Dazl*^*E8KO*^ mice and no spermatozoa were found in the lumen, while apoptotic germ cells and vacuoles were readily observable (Fig. 2A).

We next determined the furthest developmental stage that knockout germ cells could reach. After careful observation, we found a few elongated spermatids in the tubules of adult *Dazl*^*E8KO*^ mice, which were step 12 elongated spermatids based on SP10 immunofluorescence staining (Fig. 2B) [41]. Furthermore, we also observed elongated spermatids undergoing abnormal nuclear condensation, ultimately leading to apoptosis (Fig. 2B). Therefore, the absence of DAZL_FL protein led to disrupted spermiogenesis and an eventual arrest at the elongated spermatid stage.

We next investigated whether DAZL_FL deficiency induces apoptosis of spermatocytes at various developmental stages of spermatogenesis. Apoptotic cells were rarely detected at P7 in both wildtype and *Dazl*^*E8KO*^ testes (Fig. 2C). By P14, a limited number of TUNEL-positive cells could be detected in both wildtype and *Dazl*^*E8KO*^ tubules, with no statistical difference between them (Fig. 2C–E). Nevertheless, from P18 onwards, the proportion of TUNEL-positive cells in the *Dazl*^*E8KO*^ tubules was significantly higher than that in wildtype (Fig. 2C–E), indicating a notable increase in apoptosis of pachytene spermatocytes within *Dazl*^*E8KO*^ tubules, which was consistent with our histological analysis.

Next, using the VASA protein as a marker for germ cells, we determined the germ cell loss by quantifying VASA-positive cells in both wildtype and *Dazl*^*E8KO*^ testes. Our results indicated that the proportion of seminiferous tubules with complete germ cell loss in the 3-week-old *Dazl*^*E8KO*^ mice was significantly increased compared to that of wild-type mice. The reduced proportion of germ cells was observed all the way to the adult mice, with the oldest mice examined at 25-week of age in *Dazl*^*E8KO*^ mice (Fig. 2F). The substantial loss of germ cells occurring between 2 and 3 weeks of age, is consistent with our histological analysis and suggested that the apoptosis of spermatocytes starting at P18 was the primary cause for the substantial germ cell loss in the seminiferous tubules.

### The deletion of the DAZL_FL led to defects in DSB repair, XY body formation, and metaphase chromosome alignment

**Fig. 3:**
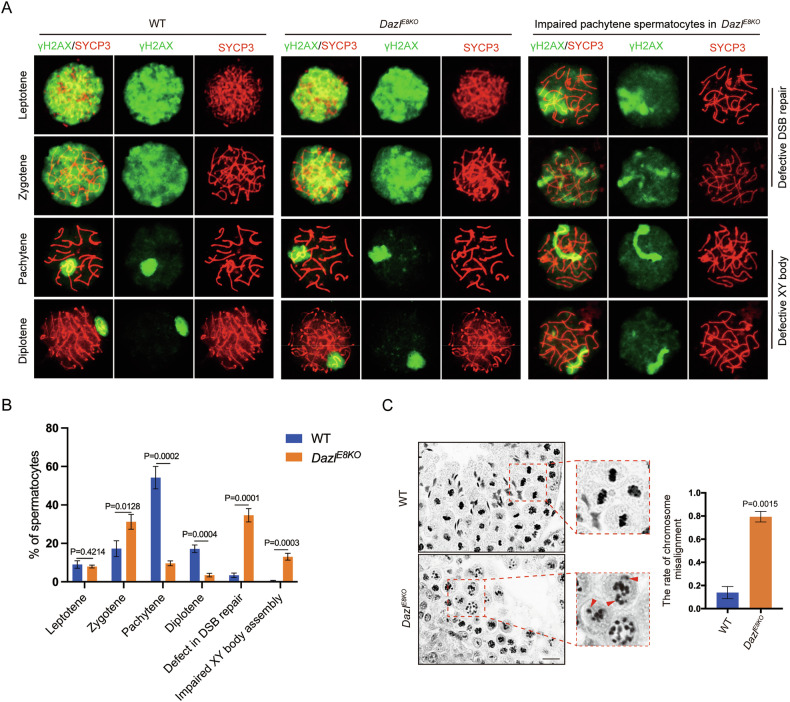
*Dazl*^*E8KO*^ male mice exhibit meiotic defects. **A** Immunofluorescent double labeling of γH_2_AX (green) and SYCP3 (red) in mouse leptotene, zygotene, pachytene, diplotene spermatocytes during metaphase I. Representative images of DSB repair defects and impaired XY body formation in *Dazl*^*E8KO*^ spermatocytes are displayed on the right panel. **B** Quantitative analysis of spermatocyte populations and defects in DSB repair and XY body formation in wildtype and *Dazl*^*E8KO*^ males. Spermatocyte counts were 1151 (wildtype) and 1285 (*Dazl*^*E8KO*^). Data presented are mean ± SD, *P* values are annotated in the histograms. **C** H&E staining in testis sections of P35 wildtype and *Dazl*^*E8KO*^ mice. Red arrowheads in red dashed boxes indicate misaligned chromosomes in metaphase I spermatocytes. The histogram represents the rate of chromosome misalignment. Data presented are mean ± SD. Scale bar, 20 μm.

We next attempted to determine the cause of the increased apoptosis in *Dazl*^*E8KO*^ testes using specific meiotic markers. The distinct phases of meiotic prophase I, namely leptotene, zygotene, pachytene, and diplotene, were characterized by the dynamic localization patterns of γH2AX [42], a marker for DNA double-strand break (DSB) formation and SYCP3 [43], a synaptonemal complex (SC) protein on chromosome spreads of meiotic prophase spermatocytes. SYCP3 was typically assembled into synaptonemal complexes in both wildtype and *Dazl*^*E8KO*^ mice (Fig. 3A). The proportion of leptotene spermatocytes in *Dazl*^*E8KO*^ mice was similar to that in wildtype mice (Fig. 3B). In wildtype testes, zygotene, pachytene, and diplotene spermatocytes accounted for approximately 20%, 60%, and 18% of the total, respectively (Fig. 3B). In comparison, zygotene spermatocytes increased to more than 30%, while pachytene and diplotene spermatocytes decreased to less than 10% and 5%, respectively (Fig. 3B). Analysis of the substages of meiotic prophase I suggested that the developmental defects in spermatocytes caused by the absence of DAZL_FL isoform were primarily observed in pachytene spermatocytes. From pachytene onwards, γH2AX staining disappeared from autosomal chromosomes and was confined to sex chromosomes, suggesting that the DSBs had been repaired (Fig. 3A). Remarkably, strong diffuse γH2AX staining was present on autosomal chromosomes of some pachytene spermatocytes in *Dazl*^*E8KO*^ testes, and the number of these spermatocytes with DSB repair defects was significantly increased compared with wildtype testes (Fig. 3A).

Furthermore, we also observed abnormalities in the XY body of pachytene spermatocytes in *Dazl*^*E8KO*^ testes (Fig. 3A). A previous study showed meiotic sex chromosome inactivation includes two genetically separable steps [44]. The linear shape of the XY body formed by the X-axis and Y-axis of sex chromosomes was highly similar to the first step of MCSI (Fig. 3A). This abnormal XY body also appeared in the testes of *Mdc1*^*-/-*^ mice [44] or *Brdt*^*-/-*^ mice [45], further confirming that DAZL_FL plays a crucial role in the MCSI process and XY body formation.

Despite significant apoptosis of pachytene spermatocytes in *Dazl*^*E8KO*^ testes, a small number of germ cells still advanced to the metaphase stage of meiosis. Through histological analysis of wildtype and *Dazl*^*E8KO*^ testes, the results indicated a very high proportion of chromosome lagging in the metaphase spermatocytes of *Dazl*^*E8KO*^ mice (Fig. 3C). Collectively, our data argued that the DAZL_FL isoform played a crucial role in DSB repair and XY body formation of pachytene spermatocytes, and chromosome alignment during the metaphase of meiosis.

### The absence of the DAZL_FL isoform disrupted the interaction between DAZL protein and the translation machinery, but not the binding to its mRNA target

**Fig. 4:**
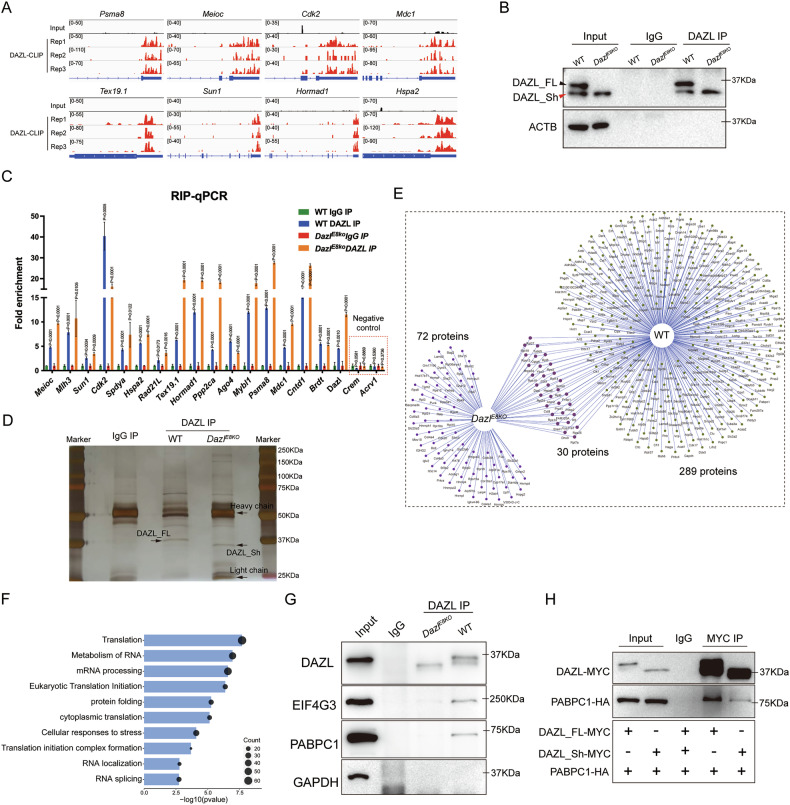
The absence of DAZL_FL protein disrupts the binding of DAZL to the translation machinery, but does not affect its enrichment of target genes. **A** Integrative genomics viewer (IGV) genome tracks showing DAZL binding peak distributions on the 3′ UTR of meiosis-associated genes. **B** RNA immunoprecipitation (RIP) was performed in WT and *Dazl*^*E8KO*^ testes, and GAPDH was used to quantify. **C** RIP-qPCR assays showing the enrichment levels of DAZL′s targets in the testes of both WT and *Dazl*^*E8KO*^ mice, with the non-target genes *Crem* and *Acrv1* serving as a negative control. Data are presented as mean ± SD, with *P* values indicated. **D** Sliver staining of DAZL immunoprecipitation (IP) in the testes of wildtype (WT) and *Dazl*^*E8KO*^ mice. IgG IP used as a negative control. **E** Venn network for DAZL interactomes in WT and *Dazl*^*E8KO*^ testes. 30 proteins are shared by the two groups. The wildtype and *Dazl*^*E8KO*^ groups possess 289 and 72 specific proteins, respectively. **F** Gene ontology (GO) enrichment analysis of 289 unique genes in WT group. **G** Co-IP assays showing the interactions between DAZL and EIF4G3 or PABPC1 in WT and *Dazl*^*E8KO*^ testes. Non-immune IgG was used as a negative control. **H** Western blot showing Co-IP of HA-tagged PABPC1 with either over-expressed MYC-tagged DAZL_FL or DAZL_Sh in HEK293T cells. IgG was used as a negative control.

We previously reported that DAZL is a master translational regulator during spermatogenesis [24]. DAZL targets identified by high-throughput sequencing of RNAs isolated by cross-linking immunoprecipitation (CLIP-seq), showed significant enrichment in pathways related to spermatogenesis and meiotic cell cycle [24, 26]. Extensive apoptosis of spermatocytes observed in the testes of *Dazl*^*E8KO*^ mice could be attributed to the translational disruption of DAZL target genes associated with meiosis due to the deficiency of DAZL_FL. To test this hypothesis, we first identified the specific binding of DAZL protein to the 3’ UTR of target genes associated with meiosis and their DAZL binding peaks at 3’ UTR region (including *Psma8*, *Meioc*, *Cdk2*, *Mdc1*, *Tex19.1*, *Sun1, Hormad1*, and *Hspa2*) using the Integrative Genomics Viewer (IGV) from our DAZL HITS-CLIP data [24] (Fig. 4A). Those genes are well-known for their essential roles during spermatogenesis [44, 46–52]. Next, we used DAZL antibody to conduct RNA immunoprecipitation (RIP) followed by qPCR (RIP-qPCR) to determine the enrichment of meiotic-related target genes that were identified by HITS-CLIP [24, 26] in both wild-type and mutant testes. Western blot analysis revealed that both wildtype and *Dazl*^*E8KO*^ testes exhibited significant enrichment of different isoforms of DAZL, with comparable enrichment levels (Fig. 4B). The RIP-qPCR results demonstrated that DAZL could significantly enrich the target genes involved in spermatogenic meiosis, with *Crem* and *Acrv1* (non-DAZL targets) serving as negative controls (Figs. 4C and S2F). Interestingly, compared to wildtype testes, the DAZL_Sh isoform in *Dazl*^*E8KO*^ testes not only bound to the same target mRNAs but also showed significantly higher enrichment of these genes (Fig. 4C), suggesting that the absence of the DAZL_FL protein does not impair the binding ability of the DAZL_Sh isoform to target genes. Consistently, the binding of DAZL to target mRNAs relies on the RNA recognition motif (RRM) domain, which is located within amino acids 40-115 of the DAZL protein. Meanwhile, exon 8 is positioned within amino acids 191-207 of the same protein, downstream RRM. Therefore, the deletion of exon 8 does not disrupt the binding ability of DAZL to its target mRNAs.

The DAZL-PABPC1 complex has been proposed to play a crucial role in translational regulation during spermatogenesis and oocyte maturation [24, 35]. Further investigation is needed to determine whether the deletion of DAZL_FL impacts the binding of DAZL to the translational machinery. We performed immunoprecipitation mass spectrometry (IP-MS) to screen and discover interacting proteins of DAZL_Sh isoform in *Dazl*^*E8KO*^ testes. The silver staining revealed that DAZL_Sh isoform could be significantly enriched by DAZL antibody, and there were numerous differential bands between WT and *Dazl*^*E8KO*^ testicular samples, suggesting alterations in DAZL interactome following the loss of DAZL_FL (Fig. 4D). Given the almost complete absence of elongated spermatids in the testes of *Dazl*^*E8KO*^ mice, we utilized the IP-MS results of DAZL in the testes of 25-day-old wildtype mice, previously published by our laboratory [24], as a control. In the testes of *Dazl*^*E8KO*^ mice, we identified 102 DAZL_Sh interacting proteins (Fig. 4E). Comparison with wildtype mice revealed 30 common proteins and 289 proteins specific to the wildtype group (Fig. 4E). As expected, these 289 proteins were predominantly associated with GO terms such as translation, RNA metabolism, mRNA processing, eukaryotic translation initiation, and cytoplasmic translation (Fig. 4F). Subsequently, we demonstrated that the absence of DAZL_FL affects the formation of the DAZL-PABPC1 complex by Co-IP assays in wildtype and *Dazl*^*E8KO*^ testes (Fig. 4G). Additionally, DAZL protein lost its ability to bind to the translation initiation factor EIF4G3 in *Dazl*^*E8KO*^ testes (Fig. 4G). We also validated the interaction of DAZL and PABPC1 in HEK293 cells by overexpressing tag-labeled proteins (MYC-tagged DAZL_FL, MYC-tagged DAZL_Sh, and HA-tagged PABPC1). The results of Co-IP assays in vitro were similar to those obtained in testes, showing that the binding ability of DAZL_Sh to PABPC1 was significantly lower than that of DAZL_FL (Fig. 4H). Therefore, we hypothesized that the impaired binding of DAZL_Sh to PABPC1 in the testes of *Dazl*^*E8KO*^ mice might affect the translation of target genes.

### Loss of the DAZL_FL isoform led to the translational downregulation of meiotic genes

**Fig. 5:**
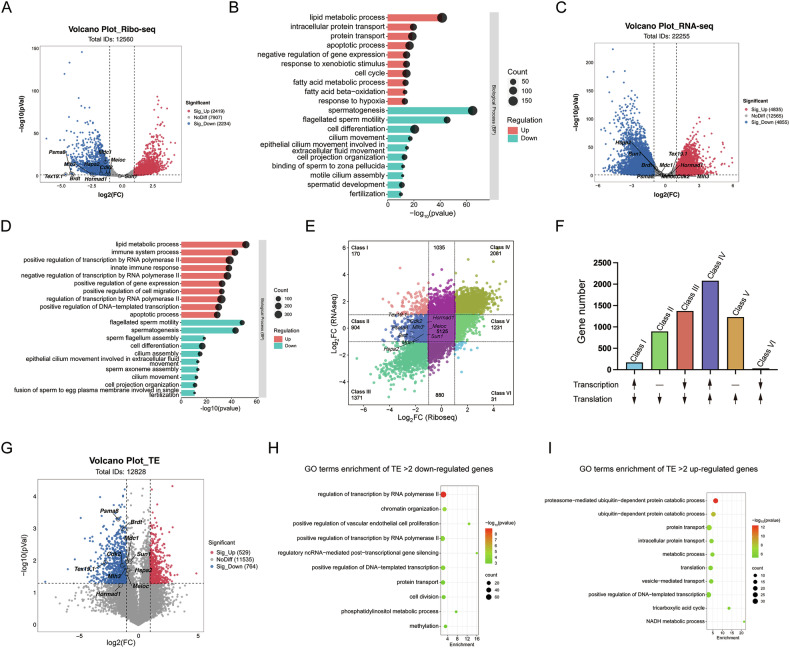
Combined analysis of Ribo-seq and RNA-seq reveals the impact of DAZL_FL isoform deletion on the translatome and transcriptome in mouse testes. **A** Volcano plot depicts differentially expressed genes (DEGs) of Ribo-seq obtained from WT and *Dazl*^*E8KO*^ testes. The meiotic genes targeted by DAZL are labeled on the plot. **B** GO analysis of DEGs from Ribo-seq. The size of each bubble represents the number of genes involved in that particular GO term. Additionally, each bar indicates a corresponding *P*-value. **C** The volcano plot represents the DEGs obtained from RNA-seq of WT and *Dazl*^*E8KO*^ testes. The meiotic genes targeted by DAZL have been marked out. **D** GO analysis of DEGs from RNA-seq. Each bar represents the corresponding *P*-value, and the size of each bubble corresponds to the number of genes involved in that particular GO term. **E** Scatter plot showing the changes in gene translation and transcription in WT and *Dazl*^*E8KO*^ testes. Class I denotes genes with down-regulated translation but up-regulated transcript in *Dazl*^*E8KO*^ testes. Class II denotes genes with down-regulated translation but transcriptionally constant in *Dazl*^*E8KO*^ testes. Class III denotes genes with down-regulated translation and transcription in *Dazl*^*E8KO*^ testes. Class IV denotes genes with up-regulated translation and transcription in *Dazl*^*E8KO*^ testes. Class V denotes genes that exhibit up-regulated translation in *Dazl*^*E8KO*^ testes while maintaining constant transcription levels. Class VI denotes genes with up-regulated translation but down-regulated transcript in *Dazl*^*E8KO*^ testes. The number of genes in each class is annotated in the plot. **F** Upward arrow represents up-regulation of translation or transcription in *Dazl*^*E8KO*^ testes. The downward arrow indicates down-regulation of translation or transcription in *Dazl*^*E8KO*^ testes. Horizontal line indicates gene expression remains stable in wildtype and *Dazl*^*E8KO*^ testes. **G** Volcano plot depicting translation efficiency (TE) changes between WT and *Dazl*^*E8KO*^ testes. A ∣log2 fold change ∣ > 1 and *P* < 0.05 indicate significant upregulation or downregulation. **H** Bubble plot showed the significantly enriched GO terms of TE down-regulated genes. **I** Bubble plot showed the significantly enriched GO terms of TE up-regulated genes.

To better understand the underlying mechanisms that cause the meiotic defects during spermatogenesis of *Dazl*^*E8KO*^ testes, we performed ribosome profiling sequencing (Ribo-seq) and bulk RNA sequencing (RNA-seq) on testes from 8-week-old wildtype and *Dazl*^*E8KO*^ mice. Compared to wildtype testes, 2234 genes were downregulated and 2419 genes were upregulated in translation in *Dazl*^*E8KO*^ testes (Fig. 5A), indicating extensive dysregulation of testicular translatome. Gene ontology analysis revealed that the downregulated genes in translation were primarily associated with “spermatogenesis”, “flagellated sperm motility”, “cell differentiation” and “cilium movement” (Fig. 5B), while the upregulated genes were mainly linked to “lipid metabolic process” and “apoptotic process” (Fig. 5B). These data implied the spermatogenesis defects observed in *Dazl*^*E8KO*^ mice might be attributed to the translational downregulation of spermatogenic genes. Additionally, RNA-seq results showed that the absence of DAZL_FL protein had a significant impact on the testicular transcriptome as well, with 4855 genes downregulated and over 4000 genes upregulated (Fig. 5C). The downregulated genes at the transcript level were predominantly enriched in “spermatogenesis”, “sperm flagellum assembly” and “cell differentiation”, while the upregulated genes were mainly associated with “lipid metabolic process”, “immune system process” and “apoptotic process” (Fig. 5D).

To rule out the possibility that the dramatic transcriptome alterations in adult *Dazl*^*E8KO*^ testes resulted from off-target effects of CRISPR-Cas9 editing, we performed RNA-seq analysis of postnatal day 16 (P16) wild type and *Dazl*^*E8KO*^ testes (Fig. S3), a developmental stage preceding major germ cell depletion. The transcriptomes showed strong correlation between genotypes (Fig. S3A), with only 11 differentially expressed genes (2 downregulated, 9 upregulated) (Fig. S3B), suggesting minimal baseline disruption from the knockout procedure. Instead, the profound transcriptomic changes observed in adult *Dazl*^*E8KO*^ testes likely stem from two interrelated factors: (1) Altered germ cell composition, as DAZL_FL deficiency triggers massive spermatocyte apoptosis, depleting meiotic and post-meiotic cell populations; and (2) Indirect transcriptional dysregulation, wherein DAZL_FL’s loss impairs the posttranscriptional regulation of transcription factor mRNAs it normally binds [24], creating downstream cascades of gene expression changes. These data imply that the transcriptome remodeling is not a direct consequence of DAZL’s transcriptional regulation but rather a secondary effect of germ cell loss and disrupted mRNA homeostasis.

Next, we performed a combined analysis with translatome and transcriptome to better characterize the molecular defects in *Dazl*^*E8KO*^ testes. Differentially expressed genes of translatome were categorized into six classes according to their transcriptional levels. The alterations in the translatome and transcriptome of genes in Class III and Class IV were consistent (Fig. 5E, F), indicating that the enhancement or suppression of their translational activity was dependent on their transcriptional levels. The biological process related to “spermatogenesis” and “apoptotic process” was enriched in Class III and Class IV, respectively (Fig. S4C, D). Genes in Class I and Class VI exhibited a negative correlation between transcription and translation, and the biological functions associated with these genes were presented in Fig. S4. Intriguingly, many of the genes that showed increased translation in *Dazl*^*E8KO*^ testes (specifically, those in Class IV and VI) were related to the “apoptotic process” (Fig. S4D, F), which coincided with the phenotype of massive spermatocyte apoptosis induced by the absence of the DAZL_FL isoform. Compared to the wildtype testes, the transcriptional levels of genes in Class V did not exhibit significant differences in *Dazl*^*E8KO*^ testes, whereas their translational levels were markedly elevated (Fig. 5E, F). These genes were primarily associated with “protein transport” and “translation” (Fig. S4E), suggesting a compensatory increase in the translation of genes related to the translational machinery following the deletion of DAZL_FL. Additionally, the translation of Class II genes was repressed, yet these genes were observed to maintain constant transcription levels (Fig. 5E, F). Notably, GO analysis showed that many genes in Class II were significantly enriched in “DNA damage response”, “double-strand break repair via homologous recombination”, “homologous chromosome pairing at meiosis”, “spermatogenesis” and “DNA repair” (Fig. S4B), suggesting the expression of genes involved in regulating meiotic process was translationally repressed in *Dazl*^*E8KO*^ testes, without significant changes in their transcriptional expression. Many of these genes associated with meiotic process were target genes of DAZL protein, including *Tex19.1*, *Mdc1*, *Psma8*, *Cdk2*, *Mlh3*, *Brdt*, *Hspa2*, *Meioc*, *Sun1*, *Hormad1* (Figs. 4C and 5E).

Furthermore, we evaluated the correlation between transcription and translation by translation efficiency (TE). Indeed, 764 genes showed a low TE (log_2_TE < −1), but 529 genes had a high TE (log_2_TE > 1) (Fig. 5G), further indicating the translation of many genes was altered in *Dazl*^*E8KO*^ testes. GO terms with low TE were significantly enriched in “regulation of transcription by RNA polymerase II” and “chromatin organization” (Fig. 5H). In contrast, GO terms with high TE included “ubiquitin-dependent protein catabolic process” and “protein transport” (Fig. 5I). According to the analysis, meiotic genes of DAZL targets, such as *Psma8*, *Brdt*, *Cdk2*, *Tex19.1* and *Mlh3* were clearly down-regulated in translation efficiency (Fig. 5G). Therefore, we hypothesize that the absence of DAZL_FL in the testes leads to the translational downregulation of these proteins, which in turn disrupts the processes of DSB repair, XY body formation, and metaphase chromosome alignment.

### The translational promotion of meiotic genes by DAZL protein relied on its binding to PABPC1

**Fig. 6:**
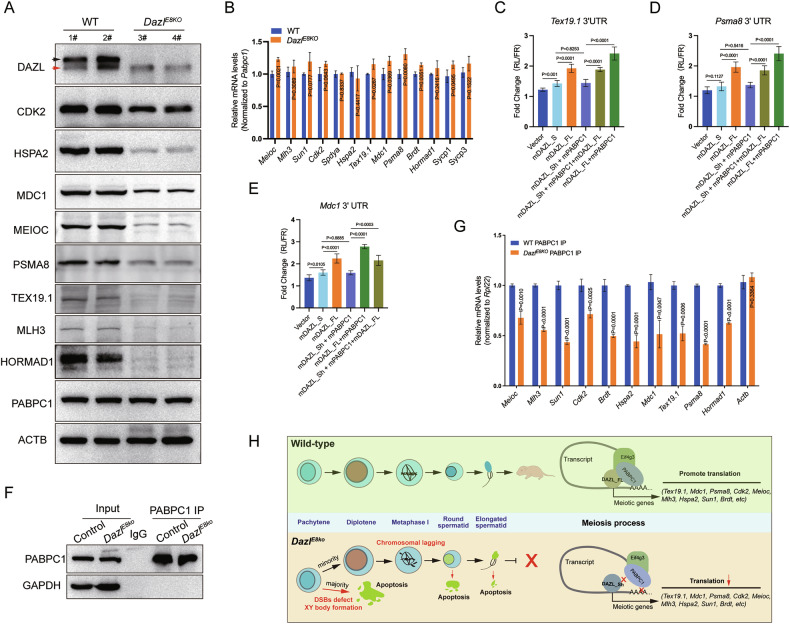
The expression of meiotic genes bound by DAZL relies on PABPC1 binding to their transcripts. **A** Western blot analysis demonstrated the protein levels of meiotic genes in the testes of postnatal day 18 (P18) wildtype (WT) and *Dazl*^*E8KO*^ mice. PABPC1 and ACTB were used as loading controls. 1#-4# represent different mice. **B** Comparison of the expression levels of 13 meiotic genes bound by DAZL in WT and *Dazl*^*E8KO*^ testes at P18 using RT-qPCR. The data are presented as means ± SD, with *P* values indicated. **C**–**E** Reporter plasmids containing the 3′ UTRs of *Tex19.1*, *Pmsa8*, or *Mdc1* were co-transfected with their respective dual-luciferase effector plasmids. The effector plasmids included an empty vector, individual constructs of DAZL_Sh or DAZL_FL, pairwise combinations of DAZL_Sh or DAZL_FL with PABPC1, and a triple combination of DAZL_FL, DAZL_Sh, and PABPC1. The relative luciferase activity was measured 48 hours later. Data are presented as mean ± SD, with *P* values indicated. **F** The RNA immunoprecipitation experiment for PABPC1 was conducted in wildtype and *Dazl*^*E8KO*^ testes with similar expression levels of PABPC1 protein, GAPDH served as a loading control. **G** The enrichment level of PABPC1 on meiosis-related genes in wildtype and *Dazl*^*E8KO*^ testes by RIP-qPCR. Actb, a non-DAZL target gene, was used as a negative control. Data are presented as mean ± SD, with *P* values indicated. **H** The schematic diagram shows the DAZL_FL isoform, rather than the DAZL_Sh isoform, forming a protein complex with PABPC1, thereby regulating the translation of meiosis-related genes.

Based on the histological analysis of the first spermatogenic wave in *Dazl*^*E8KO*^ testes, we found that a large number of spermatocytes had begun to undergo apoptosis from P18 (Fig. 2A, C). Therefore, we selected the testes at P18 for the expression validation of target genes. To explore whether the absence of DAZL_FL could affect the expression of meiosis-associated proteins, we determined the expression patterns of DAZL-bound meiotic genes in *Dazl*^*E8KO*^ testes. To minimize cellular composition bias caused by germ cell apoptosis in the mutant testes, we performed synergistic quantification of samples using both PABPC1, which is highly expressed in germ cells, and the conventional internal reference protein ACTB as controls. Western blotting verified decreased expression of CDK2, HSPA2, MDC1, MEIOC, PSMA8, TEX19.1, HORMAD1 and MLH3 (Fig. 6A). However, the mRNA expression of these genes in the *Dazl*^*E8KO*^ testes at P18 either did not decrease or even increased in some cases (Fig. 6B). These results suggested that the deletion of DAZL_FL resulted in a decrease in the protein expression of meiotic genes at the translational level.

To confirm the effect of DAZL-PABPC1 complex on the regulation of these candidate mRNAs, dual luciferase reporter assays were conducted. We selected DAZL targets, *Tex19.1*, *Mdc1*, and *Psma8*, which have been well-established to be associated with defects in DSB repair [48], XY body formation [44], and meiotic division [46], while these defects were also present in testes with DAZL_FL deficiency. Independent experiments in HEK293T cells demonstrated that DAZL_Sh significantly enhanced the luciferase reporter activities of the 3’ UTRs of *Tex19.1* and *Mdc1* (Fig. 6C, E), but not that of *Psma8* 3’ UTR (Fig. 6D). In contrast, DAZL_FL could markedly elevate the dual luciferase signals of all three genes and exhibited a significantly higher ability to promote the expression of target genes compared to DAZL_Sh (Fig. 6C–E). When DAZL_Sh was co-expressed with PABPC1, the luciferase activities of the reporter plasmids for *Tex19.1* (Fig. 6C), *Psma8* (Fig. 6D), and *Mdc1* (Fig. 6E) did not show any significant difference compared to when only DAZL_Sh was expressed. Strikingly, the introduction of DAZL_FL into the DAZL_Sh and PABPC1 combination robustly enhanced the reporter activity of all target genes, indicating that DAZL_FL can functionally compensate for or even reverse the limited activity of DAZL_Sh (Fig. 6C–E). Furthermore, the luciferase activities of the reporter plasmids were markedly increased when DAZL_FL was co-expressed with PABPC1 alone, compared to when DAZL_Sh was co-expressed with PABPC1 (Fig. 6C–E). The luciferase activity of the *Tex19.1* 3’ UTR reporter plasmid increased by more than 1.6-fold, the luciferase activity of the *Psma8* 3’ UTR reporter plasmid increased by more than 1.8-fold, and the luciferase activity of the *Mdc1* 3’ UTR reporter plasmid increased by more than 1.7-fold (Fig. 6C–E). Therefore, the formation and stabilization of the DAZL-PABPC1 complex played a crucial role in promoting the expression of DAZL target genes.

To further investigate the molecular links between the DAZL-PABPC1 complex and DAZL target transcripts, we performed RIP-qPCR analysis using PABPC1 antibody and confirmed the enrichment of PABPC1 on DAZL targets in both wildtype and *Dazl*^*E8KO*^ testes. The Western blot results indicated that the expression level of PABPC1 protein was consistent in the input groups of both wildtype and *Dazl*^*E8KO*^ testes. Additionally, there was no notable difference in the enrichment level of PABPC1 in the testes of both groups through immunoprecipitation experiments (Fig. 6F), suggesting the feasibility of conducting subsequent qPCR experiments. Not surprisingly, comparisons with the wildtype testes suggested a significant decrease in the ability of PABPC1 to bind to the transcripts of DAZL targets in *Dazl*^*E8KO*^ testes, which included numerous genes related to meiosis, such as *Tex19.1*, *Mdc1*, *Psma8*, *Cdk2*, *Meioc*, *Brdt*, *Mlh3*, *Sun1*, *Hspa2*, *Hormad1*, among others (Fig. 6G). Therefore, the binding of PABPC1 to the mRNAs of DAZL targets depends on the recruitment of PABPC1 protein by DAZL_FL.

It has been well known that PABPs activate the translation of mRNAs by interacting with RNA-binding proteins and specifically bind to poly(A) tails at the 3′ ends of mRNAs in spermatogenic cells [24, 53]. Based on the analysis above, we proposed that DAZL_FL forms a protein complex with PABPC1, rather than the DAZL_Sh isoform, thereby facilitating the translation of meiotic genes directly bound by DAZL and ensuring meiotic biological processes such as DSB repair, XY body formation, and chromosome alignment (Fig. 6H).

### The DAZL_FL isoform is essential for meiosis in folliculogenesis

**Fig. 7:**
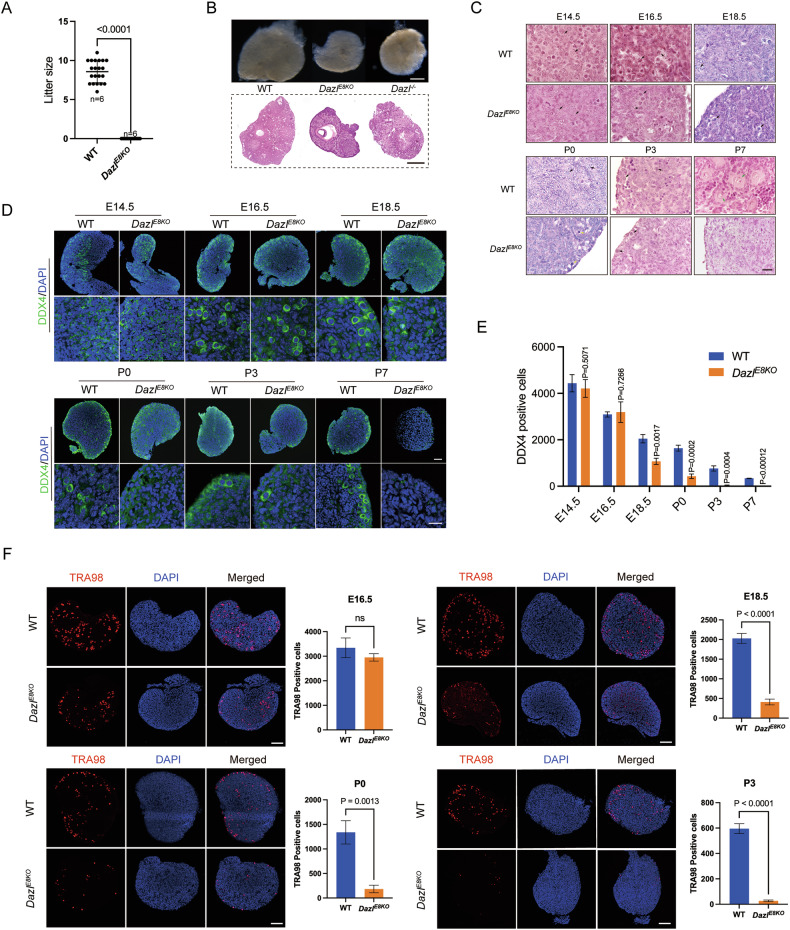
*Dazl*^*E8KO*^ female mice were infertile and suffered a significant decrease in the number of germ cells at perinatal ovaries. **A** Number of pups per litter produced by crossing 8-week-old *Dazl*^*E8KO*^ females (*n* = 6) with age-matched WT males (*n* = 6). **B** Representative images of ovaries from WT, *Dazl*^*E8KO*^, and *Dazl*^*-/-*^, along with corresponding H&E staining images of the ovaries. Scale bar, 500 μm. **C** The histology of mouse ovaries from embryonic day 14.5 (E14.5) to postnatal day 7 (P7). Black arrows indicate germ cells, yellow arrows indicate apoptotic germ cells, and green arrows represent oocytes of primary follicles. Scale bar, 10 μm. **D** Immunofluorescence staining for DDX4 (green) to indicate germ cells in WT and *Dazl*^*E8KO*^ perinatal ovaries. Scale bar, 20 μm. **E** Germ cell counts in the ovaries of wildtype and *Dazl*^*E8KO*^ mice from embryonic day 14.5 (E14.5) to postnatal day 7 (P7), with DDX4 signal (green) used as the marker. Data are presented as mean ± SD, with *P* values indicated. **F** Immunofluorescence staining of TRA98 (red) for labeling germ cells, and quantification of oocytes in WT and *Dazl*^*E8KO*^ female ovaries at E16.5, E18.5, P0 and P3. Data presented are mean ± SD, with *P* values indicated. ns not significant. Scale bar, 50 μm.

Previous reports have shown that DAZL is a critical factor for the initiation of meiosis in the ovaries of female embryonic mice [31, 54]. To investigate whether DAZL_FL plays a role in female meiosis and is essential for folliculogenesis, we conducted a comprehensive analysis of *Dazl*^*E8KO*^ female mice. When *Dazl*^*E8KO*^ female mice were crossed with wildtype male mice, no pups were obtained over a period of 6 months, suggesting that *Dazl*^*E8KO*^ female mice were completely infertile (Fig. 7A). The size of ovaries in 6-week-old *Dazl*^*E8KO*^ females was obviously reduced compared with that of wildtype littermates, yet similar in size to those of *Dazl*^*-/-*^ ovaries (Fig. 7B). H&E staining results showed that typical ovarian follicles were absent in *Dazl*^*E8KO*^ females, and occasionally, a cavity without an oocyte was visible (Fig. 7B). Further study found that apoptotic nuclei were first noted at P0 and no primary follicle was detected at P7 in *Dazl*^*E8KO*^ ovaries (Fig. 7C). Subsequently, we determined the timing of germline cell loss by immunofluorescence staining of DDX4 and TRA98, both of which are germ cell markers. The results of quantitative analyses showed DDX4-positive germ cells was significantly decreased in *Dazl*^*E8KO*^ ovaries compared with control ovaries from E18.5 (Fig. 7D, E), the germ cell loss at E18.5 was further confirmed using TRA98 immunofluorescence (Fig. 7F). Thus, the significant drop of germ cell number at E18.5 ovaries but not at E16.5 ovaries indicated germ cell loss at meiotic prophase, most likely pachytene stage, similar to the stage of spermatocyte loss in the testis. DAZL_FL isoform is crucial for post-migratory germ cell survival and meiotic progression in the ovary. We would like to propose that DAZL_FL isoform play a key role in meiotic progression of both males and females.

## Discussion

Spermatogenesis, a precisely regulated biological process essential for the production of male gametes, is conventionally categorized into three distinct stages: the maintenance and differentiation of spermatogonia, the meiotic division of spermatocytes, and the subsequent maturation of haploid spermatozoa [55]. Alternative splicing is crucial for male gamete biogenesis, driving intricate molecular mechanisms that give rise to one of the most complex and dynamic tissue-specific transcriptomes. Recent studies have identified and validated numerous regulators of alternative splicing that play significant roles in spermatogenesis [2, 3, 7]. However, there is limited research further exploring the specific functions of protein isoforms generated through alternative splicing. Previous research has identified two distinct isoforms of the DAZL protein in mice and elucidated their functional roles in embryonic stem cells using in vitro experimental approaches [34]. To further investigate the biological significance of these isoforms, we generated a *Dazl* exon 8 knockout mouse model, enabling us to delineate their specific contributions to the process of spermatogenesis. In current study, we discovered that the DAZL_FL isoform is essential for male meiosis and spermiogenesis, whereas DAZL_Sh is sufficient to support embryonic germ cell specification, PGC development and differentiation, all the way to postnatal spermatogonial maintenance and differentiation. Additionally, we observed post-migratory germ cell loss at meiotic prophase in embryonic ovaries and impaired primordial follicle development in *Dazl*^*E8KO*^ female mice, supporting that DAZL_FL plays a critical role in meiotic prophase in both sexes. The translational regulation of meiotic genes by DAZL depends on the formation of a complex between DAZL_FL and PABPC1.

Previous research demonstrated that full knockout or germ cell-specific deletion of *Dazl* in C57B6 background resulted in the absence of germ cells in the testes [21, 24]. In this study, we discovered that the DAZL_Sh isoform alone is sufficient to ensure the maintenance and differentiation of spermatogonia, suggesting that DAZL_FL protein is dispensable for spermatogonial development (Fig. S2). Mikedis et al. demonstrated that DAZL governed the translational regulation of numerous transcripts in undifferentiated spermatogonia, particularly those involved in spermatogonial expansion, spermatogonial differentiation, transcription, and RNA splicing [25]. Interestingly, DAZL and PABPC1 proteins displayed synchronized high expression in the testes beginning at postnatal day 12, a pattern that persisted until the completion of the first spermatogenic wave (Fig. 1B). This temporal expression profile further implies that the canonical DAZL/PABPC1 complex is not essential for DAZL-mediated translational regulation of target genes in spermatogonia. Therefore, the biological functions of DAZL_FL and DAZL_Sh proteins in spermatogonia appear to be redundant. However, upon meiotic entry, the deletion of DAZL_FL led to meiotic defects and extensive spermatocyte apoptosis, highlighting the DAZL/PABPC1 complex as a pivotal regulator of DAZL-mediated translational control of target transcripts.

Previous studies employing yeast two-hybrid assays identified critical regions mediating the interaction between mouse DAZL protein and PABPC1 protein, revealing that the region immediately downstream of the DAZ repeat domain is critical for PABPC1 binding [27]. Notably, DAZL exon 8 is positioned directly downstream of the DAZ repeat domain. Furthermore, recent investigations demonstrated that C-terminal truncation in DAZL impaired its interaction with PABPC1 [56]. Collectively, these findings suggest that the region downstream of the DAZ repeat domain, despite lacking well-defined functional domains, is essential for DAZL-PABPC1 complex formation and the subsequent translational regulation of target mRNAs.

Our findings demonstrating that DAZL requires PABPC1 interaction for its meiotic function align with emerging paradigms of RNA-binding protein (RBP) regulation during gametogenesis. Multiple RBPs, including hnRNPC, YTHDC2, and RBM46, have been shown to regulate meiosis through the formation of specific protein complexes, similar to the DAZL/PABPC1 complex we characterized. hnRNPC partners with HUR to regulate alternative splicing in an m^6^A-dependent manner [57]. YTHDC2 forms a complex with MEIOC that is involved in RNA regulation [58]. RBM46, YTHDC2, and MEIOC function together to regulate meiotic entry [59]. This recurring theme of RBP complex formation suggests it represents a fundamental regulatory strategy for precise spatiotemporal control of meiotic gene expression. The composition and dynamic regulation of these complexes are likely to determine their functional specificity. Our identification of exon 8 as critical for DAZL/PABPC1 interaction provides a molecular framework for understanding how alternative splicing may modulate RBP complex formation during germ cell development. Future comparative studies of these different RBP complexes could reveal both shared principles and unique features of meiotic gene regulation.

Although the deletion of DAZL_FL led to widespread spermatocyte apoptosis, a small number of round spermatids were still observed in the seminiferous tubules of *Dazl*^*E8KO*^ testes, which ultimately arrested at the elongating spermatid stage and failed to produce mature sperm (Fig. 2A, B). To elucidate the underlying mechanism, we examined the expression of TNP1, TNP2, PRM1, and PRM2 proteins. The levels of these proteins were significantly reduced in *Dazl*^*E8KO*^ testes, indicating that the loss of DAZL_FL may impair the transition from histones to protamine (Fig. S5A). Additionally, we examined the mRNA and pre-mRNA levels of these genes, and the results showed a significant decrease compared to wildtype (Fig. S5B, C), indicating that DAZL_FL deletion affects the transcription of these genes. Furthermore, we observed prominent meiotic defects at the metaphase stage in DAZL_FL-deficient spermatocytes (Fig. 3G), which may result in the production of aneuploid round spermatids, potentially contributing to spermiogenesis arrest. Our previous study demonstrated that conditional knockout of *Dazl* gene in pachytene spermatocytes resulted in developmental arrest at the round spermatid stage, and DAZL was found to directly bind to multiple regulatory factors critical for spermiogenesis [24]. Thus, our study further demonstrates that DAZL_FL plays a critical role during spermiogenesis, and the formation of the DAZL/PABPC1 complex may be a prerequisite for this process.

Research in Xenopus oocytes reveals that the DAZL-PABPC1 protein complex inhibits mRNA deadenylation through a physical blocking mechanism. Specifically, DAZL facilitates prolonged PABPC1 binding to poly(A) tails, which spatially obstructs deadenylase enzymes from accessing their substrate [60]. This mechanism operates completely independently of the DAZL-PABPC1 complex′s established function in translation initiation activation. Our analysis of *Dazl*^*E8KO*^ testes revealed widespread mRNA downregulation (Fig. 5C), consistent with a model wherein disrupted DAZL-PABPC1 interactions lead to transcript deadenylation and subsequent decay. However, we identified a subset of DAZL target mRNAs that maintained stable expression levels despite DAZL_FL deficiency (Fig. 6B), revealing an unexpected selectivity in this posttranscriptional regulatory mechanism within testicular germ cells. These findings highlight the complexity of DAZL-mediated mRNA stabilization and underscore the need for further investigation into the global transcriptional consequences of DAZL_FL deletion.

Our current study dissected the functional contributions of distinct murine DAZL isoforms during spermatogenesis, and demonstrated that the DAZL/PABPC1 complex serves as a critical determinant of spermatocyte developmental progression and post-migratory germ cell survival in the ovaries. Although similar exon 8 splicing resulting in a short form of DAZL has not been detected in human DAZL and only limited to rodent species and western painted turtle (Fig. S6A), conservation of exon 8-encoded amino acids and full-length DAZL containing exon 8 from across mammals including human to chicken, turtle and even lizards support highly conserved requirement of DAZL-PABPC1 in vertebrate reproduction (Fig. S6B). The differential requirement of DAZL_FL between embryonic germ cell development and meiotic progression raised the possibility of a distinct mechanism of DAZL regulation in two stages.

The DAZL protein has shown significant clinical associations with human idiopathic azoospermia [61–64] and primary ovarian insufficiency (POI) [20, 56], indicating its potential as a promising therapeutic target. Notably, a recent study by Liu et al. demonstrated that C-terminal truncated DAZL mutations disrupt PABPC1 binding, thereby reducing NANOS3 expression and causing premature ovarian insufficiency [56]. This finding establishes a direct pathophysiological link between the integrity of the DAZL/PABPC1 complex and female fertility. This parallels our observation that DAZL_FL deficiency (lacking the PABPC1-interacting exon 8) causes primordial follicle depletion in mice (Fig. 7), suggesting conserved mechanisms across species. In male reproduction, similar mutations could underlie cases of non-obstructive azoospermia characterized by meiotic arrest, as DAZL/PABPC1-dependent translation is crucial for spermatocyte survival (Figs. 2, 3). These insights open new therapeutic possibilities: (1) Small-molecule stabilizers could be developed to enhance DAZL/PABPC1 interaction, particularly for patients with hypomorphic DAZL variants; (2) Gene therapy approaches could deliver intact DAZL_FL to germ cells. While challenges remain in targeted delivery to gonadal tissue, the conserved nature of this interaction across mammals provides a strong rationale for translational development.

## Supplementary information

Supplementary figures and legends

Supplemental Table I: primer sequence for RT-qPCR

Mass Spectrum

Expression matrix from RNA-seq data

Expression matrix from Ribo-seq data

Original Western blot images

## Data Availability

The raw RNA-seq data of P16 testes from wildtype and *Dazl*^*E8KO*^ mice have been deposited in the China National Center for Bioinformation (CNCB) (accession no. PRJCA043888). All other datasets generated or analyzed during this study are included in this published article and its supplementary information files. These data are available from the corresponding author upon reasonable request.
