# Supplementary material for: Loss of full-length DAZL isoform disrupts PABPC1-dependent translational regulation and meiosis
Source: Cell Death Dis. 2025 Nov 17;16(1):841. doi: 10.1038/s41419-025-08179-7 (PMC12623972; doi:10.1038/s41419-025-08179-7)
Supplement: Supplementary file 1 — Supplementary figures and legends [file 41419_2025_8179_MOESM1_ESM.docx]

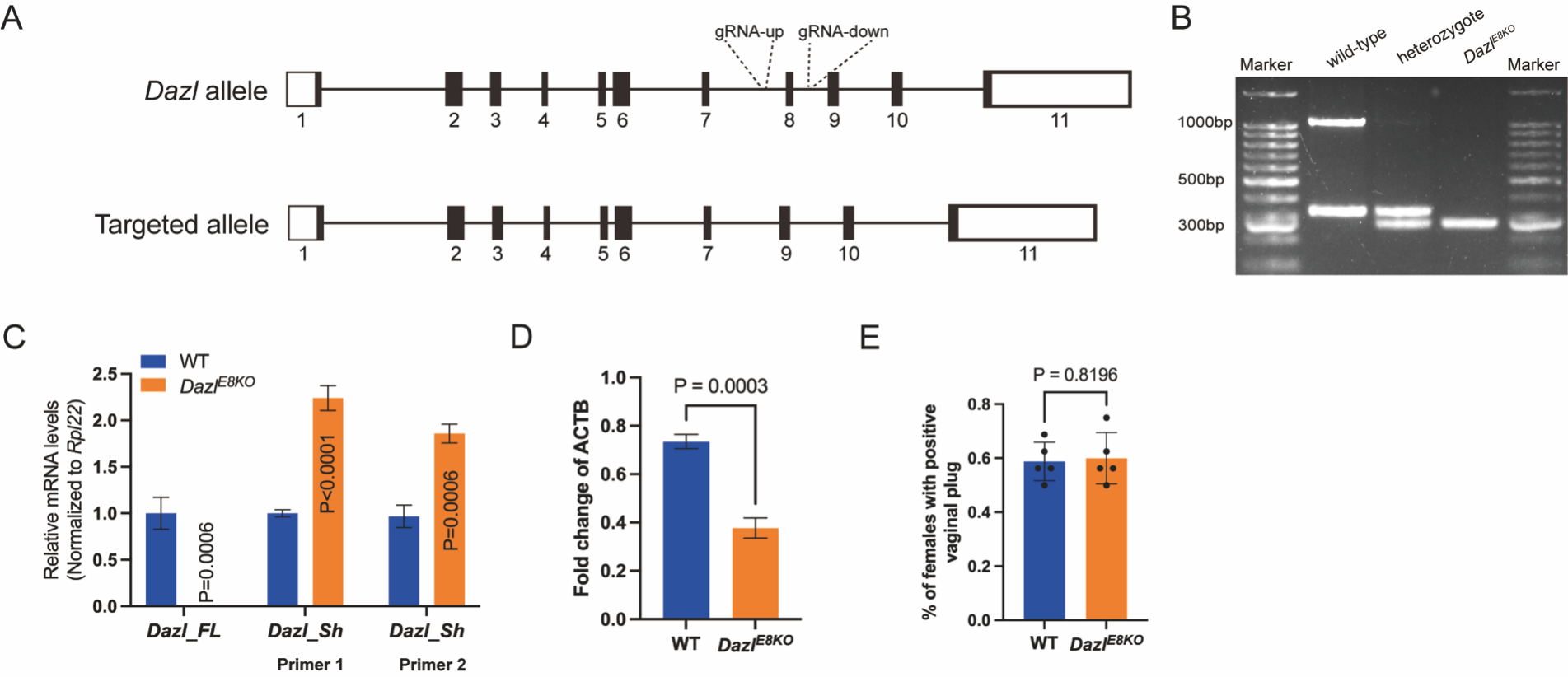


**Fig. S1: Generation of a mouse model with full-Length subtype knockout of *Dazl*.** A The schematic diagram of the strategy for knocking out the full-length transcript of *Dazl*. B DNA gel electrophoresis results showing the genotyping identification of wildtype, heterozygous, and *Dazl^E8KO^* mice. C The expression of *Dazl* full-length (*Dazl_FL*) and short (*Dazl_Sh*) transcripts in the testes of wildtype and *Dazl^E8KO^* mice, with RPL22 as the internal reference. Data are presented as mean ± SD, with P values indicated. D ImageJ software was used to analyze DAZL_Sh protein expression levels in Fig. 1C. Data are presented as mean ± SD, with P values indicated. E Bar graph showing vaginal plug formation rates in wildtype females mated with *Dazl^E8KO^* (n=8) or wildtype (n=8) male mice. Adult males of each genotype were paired with two 8-week-old WT females per mating trial. Plug formation was assessed the following morning. Five sequential trials were conducted at 2-day intervals. ns denotes no significant difference.


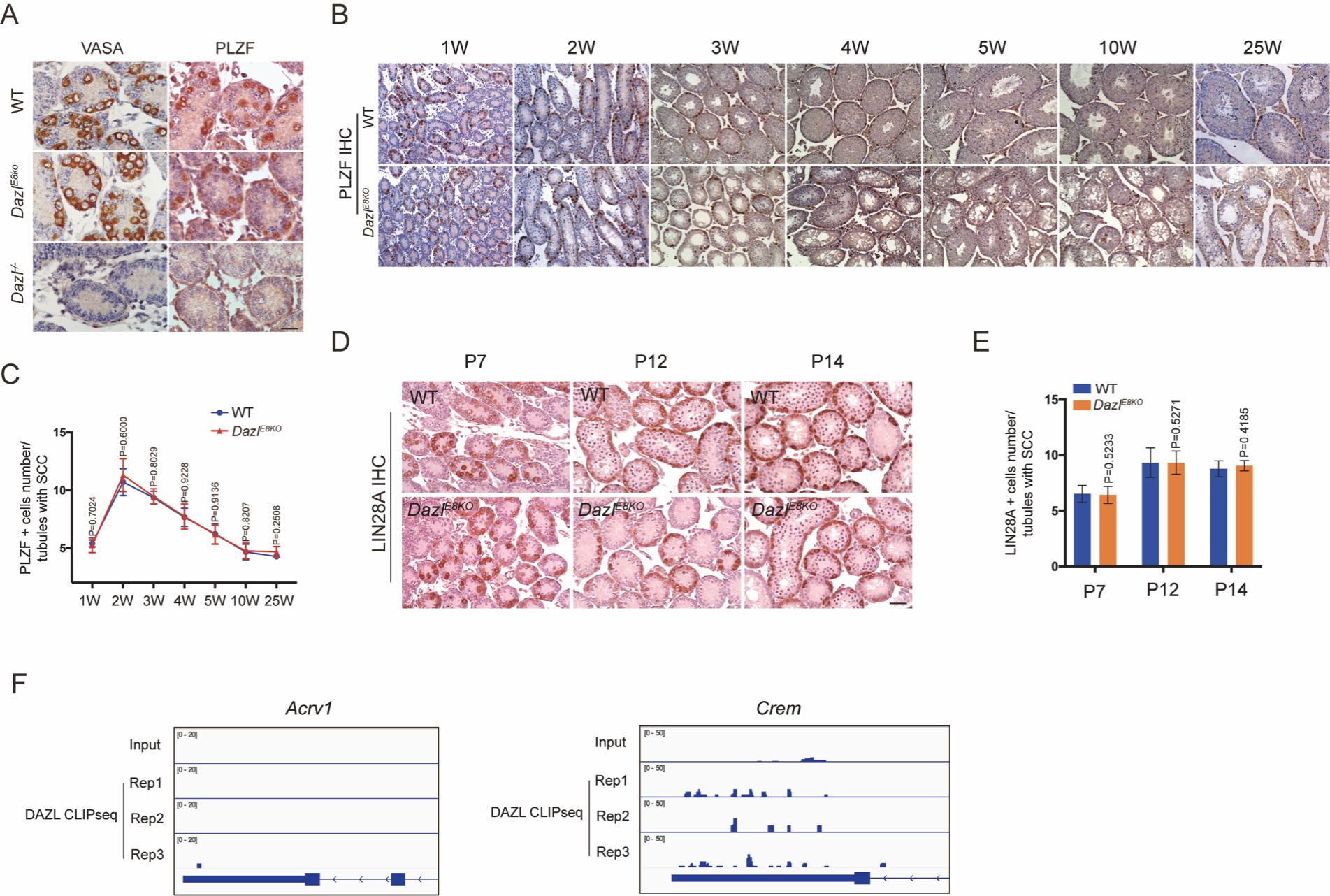


**Fig. S2: The absence of DAZL_FL isoform does not affect the maintenance of spermatogonia.** A Immunohistochemistry (IHC) analysis for VASA and PLZF staining in wildtype (WT), *Dazl^E8KO^* and *Dazl^-/-^* cross sections of P7 testes. Scale bar, 20 μm. B Cross sections of WT and *Dazl^E8KO^* testes stained with antibody for PLZF at 1, 2, 3, 4, 5, 10 and 25 weeks. Scale bar, 50 μm. C Quantitative comparison of PLZF positive cells per seminiferous tubule between WT and *Dazl^E8KO^* testes at 1, 2, 3, 4, 5, 10 and 25 weeks. Data are represented as the mean ± SD, the P-values have been marked on the line graph. D Cross sections of WT and *Dazl^E8KO^* testes stained with antibody for LIN28A at P7, P12 and P14. Scale bar, 50 μm. E Quantitative comparison of LIN28A positive cells per seminiferous tubule between WT and *Dazl^E8KO^* testes at P7, P12 and P14. Data are presented as mean ± SD, and P values are shown. F IGV genome tracks illustrating DAZL binding peak distributions across 3′ UTR regions of non-target genes.

**
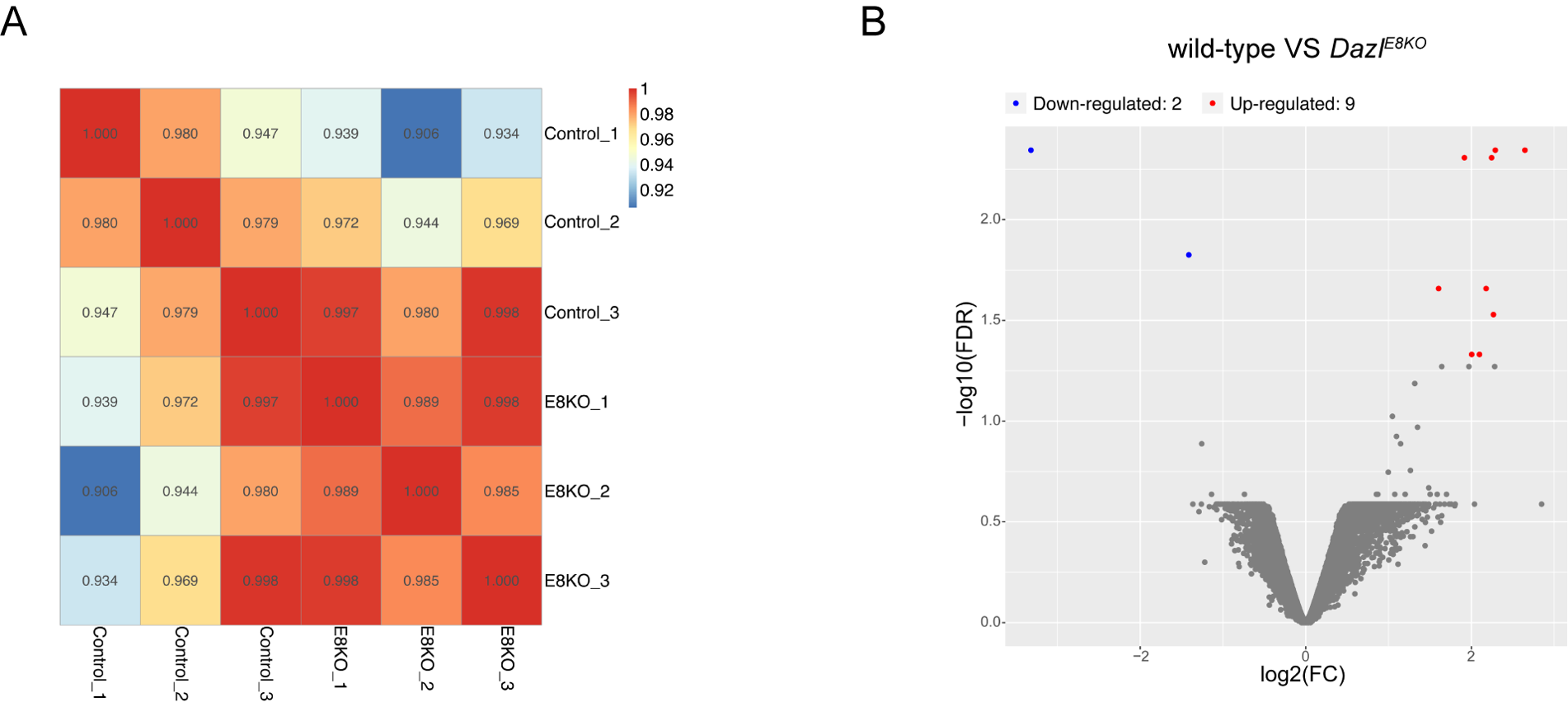
**

**Fig. S3: The absence of DAZL_FL disrupted the transition from histones to protamines in mouse testes.** A Western blot was performed to detect the expression of transition proteins and protamines in the testes of wildtype and *Dazl^E8KO^* testes, with ACTB used as an internal control for sample quantification. RT-qPCR was used to detect the mRNA (B) and pre-mRNA (C) levels of *Tnp1*, *Tnp2*, *Prm1* and *Prm*2 in the testes of wildtype and *Dazl^E8KO^* testes. **Data are expressed as mean ± SD, and P values are shown.**

**
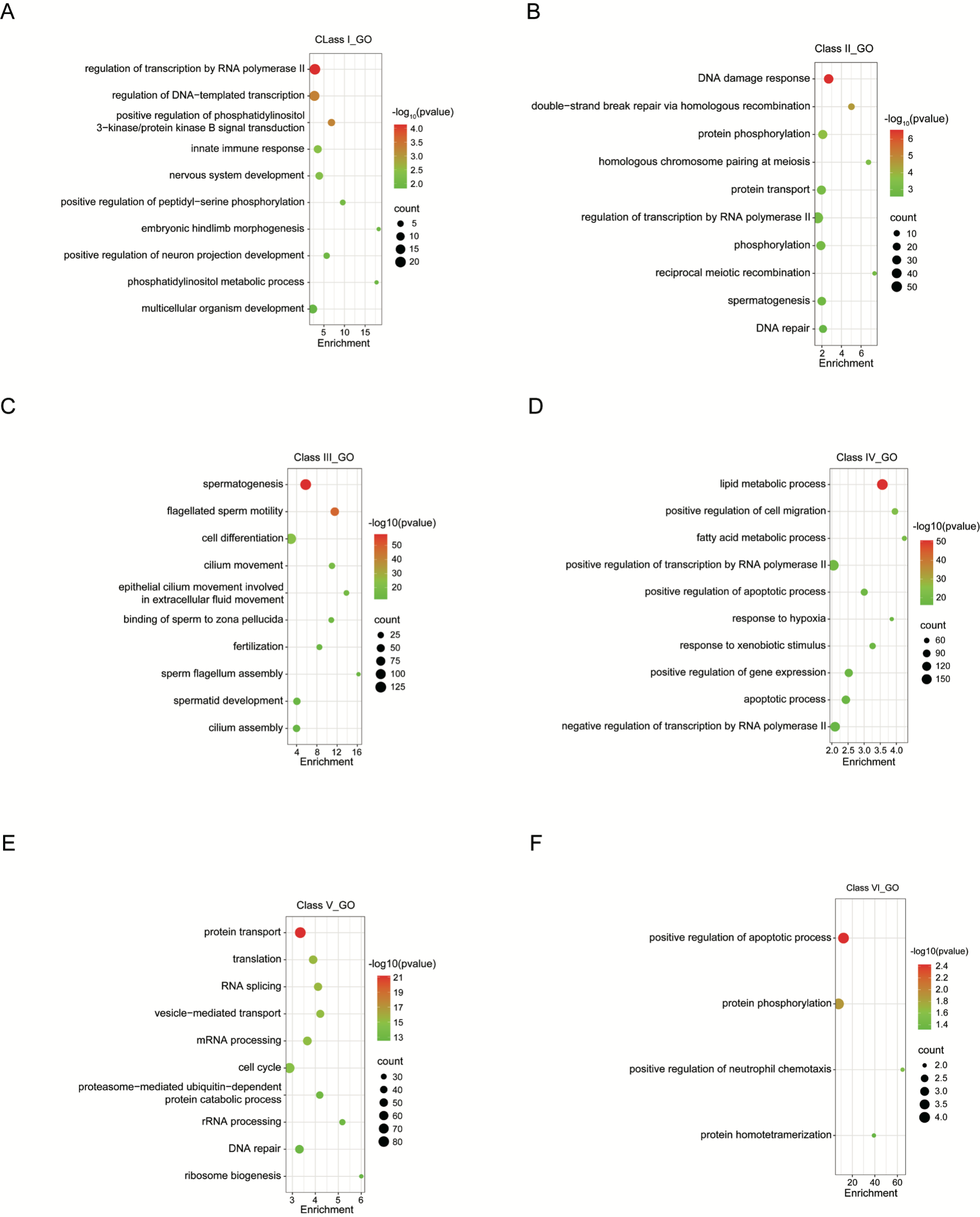
**Fig. S4: RNA-seq analysis of postnatal day 16 (P16) wildtype and *Dazl^E8KO^* testes. (A) Pearson correlation coefficient analysis between wildtype and DazlE8KO groups. (B) Volcano plot showing differentially expressed genes (DEGs) in P16 wildtype versus *Dazl^E8KO^* testes. Thresholds for DEGs: P < 0.05 and |log2FC| > 1.

**
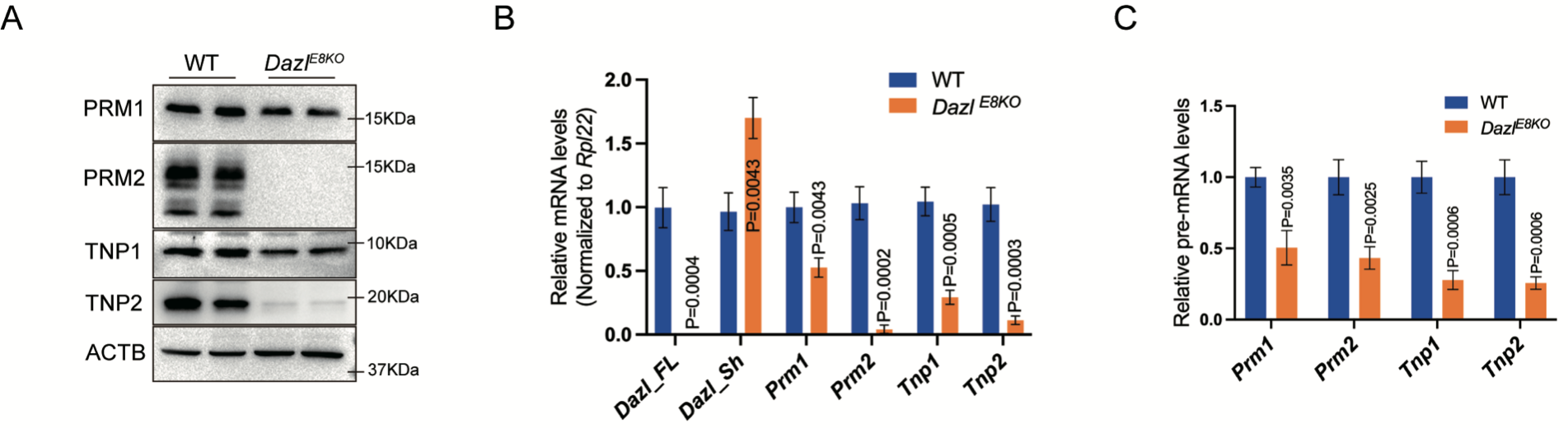
Fig. S5: Gene Ontology (GO) enrichment analysis.** GO analysis was performed for each group to identify significant biological processes associated with the genes in each category. A Genes with downregulated translation but up-regulated transcript in *Dazl^E8KO^* testes. B Genes with downregulated translation but unchanged transcription levels in *Dazl^E8KO^* testes. C Genes with co-downregulated transcription and translation in *Dazl^E8KO^* testes. D Genes with co-upregulated transcription and translation in *Dazl^E8KO^* testes. E Genes with stable transcription but upregulated translation in *Dazl^E8KO^* testes. F Genes with stable transcription but upregulated translation in *Dazl^E8KO^* testes.

**
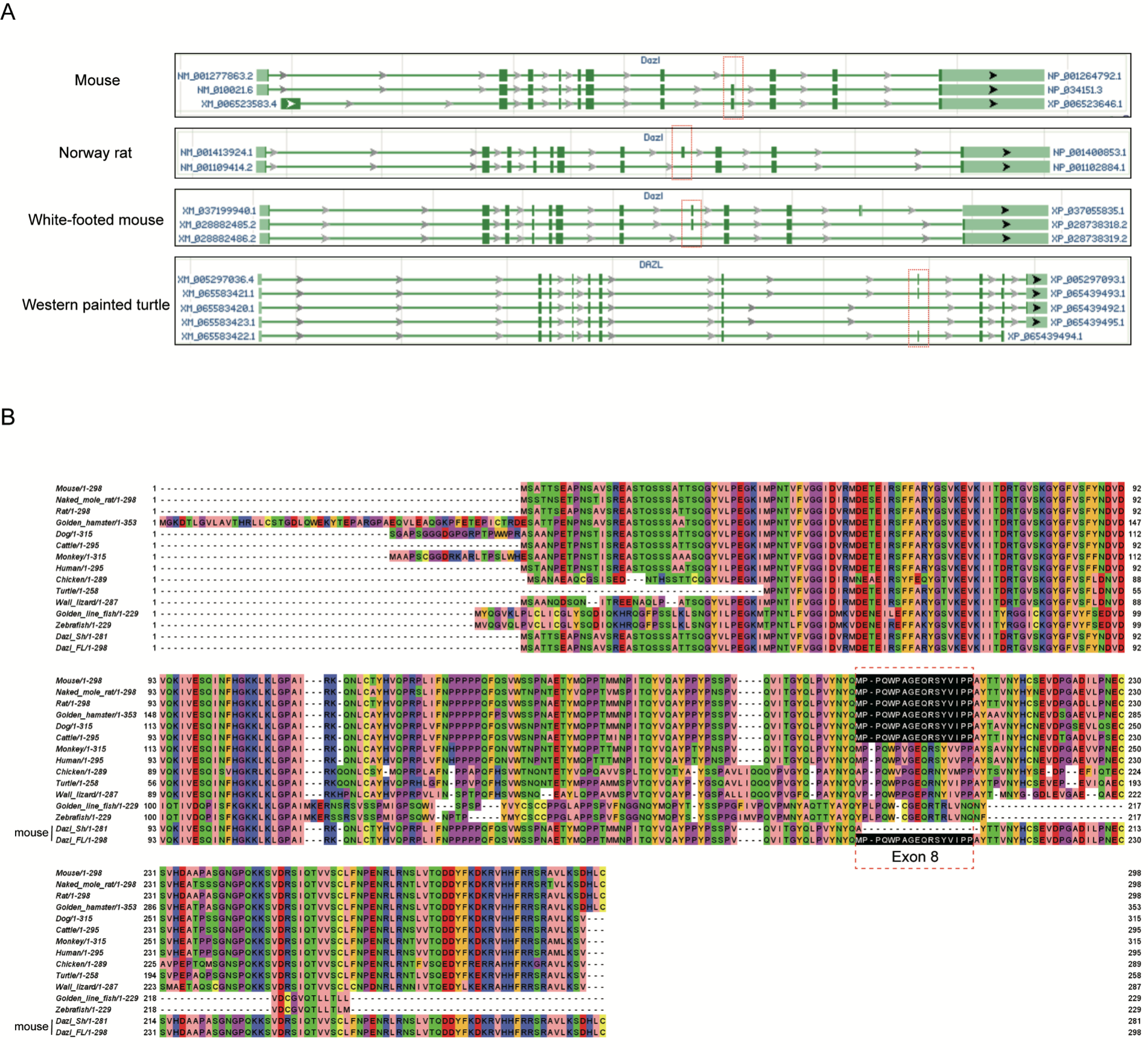
Fig. S6: Analysis of *Dazl*exon 8 skipping and sequence conservation.** A Screenshot from the NCBI database showing alternative splicing events involving *Dazl* exon 8 skipping in rodents and turtles. The red dashed box highlights the exon 8 region. B Amino acid sequence alignment of DAZL proteins from fish to human, generated using Jalview. The red dashed box indicates the exon 8-encoded region, demonstrating evolutionary conservation.
