# Supplementary material for: Loss of full-length DAZL isoform disrupts PABPC1-dependent translational regulation and meiosis
Source: Cell Death Dis. 2025 Nov 17;16(1):841. doi: 10.1038/s41419-025-08179-7 (PMC12623972; doi:10.1038/s41419-025-08179-7)
Supplement: Supplementary file 3 — Mass Spectrum [file 41419_2025_8179_MOESM3_ESM.pdf]

DAZL_IP1_E8KO

Gene Na	Descripti	Mass	Score	Matches	Sequenc	emPAI	Coverage
HC	MAb 106	51959	4693	241(167)	26(21)	10.70	46%
Ighg	Ighg prot	52711	2709	188(121)	26(20)	8.42	44%
Ighg	Ighg prot	52514	2612	187(117)	25(19)	7.41	39%
Oplah	5-oxoprc	138950	2482	130(88)	45(37)	2.76	40%
HC	MAb 31C	51376	2270	126(75)	17(13)	4.03	50%
	Uncharac	26104	2232	68(55)	10(8)	7.73	46%
Igkc	If kappa	24435	2210	83(62)	15(11)	18.13	76%
	Anti-colc	26780	2207	76(60)	14(10)	13.92	64%
Igh	Igh prote	52873	2018	166(96)	15(13)	7.82	37%
Igk	Igk prote	26570	1899	70(54)	13(9)	9.69	58%
Igkc	Kappa lig	24356	1896	72(56)	13(10)	12.17	55%
Igk	Igk prote	25971	1870	67(52)	11(9)	11.67	54%
HC	MAb 44E	52527	1859	164(92)	18(16)	11.12	42%
Oplah	Uncharac	92528	1793	85(59)	29(23)	2.50	38%
Igkv8-30	ENSMUS	26934	1763	79(60)	10(8)	7.15	42%
	Uncharac	52407	1742	167(92)	16(15)	10.47	41%
LC	MAb 110	26858	1695	63(49)	11(8)	7.22	47%
LC	MAb 106	26605	1681	64(51)	11(9)	9.59	55%
	Fab4201	24356	1675	61(48)	10(8)	9.17	48%
Igk	Igk prote	26086	1654	64(49)	11(9)	8.85	47%
LC	MAb 6H1	26372	1625	62(48)	11(8)	7.51	46%
Igk	Light cha	25972	1594	65(46)	10(7)	7.81	46%
	Anti-H5N	51702	1519	100(56)	14(11)	3.14	41%
Igh	Igh prote	52684	1470	109(58)	18(14)	3.83	50%
Igh	Igh prote	51659	1378	97(53)	12(9)	2.67	36%
	Anti-VIPa	12060	1362	22(22)	4(4)	6.38	51%
	Ig gamm	37086	1307	113(68)	9(8)	8.25	27%
Ighv1-81	Immunog	13094	1278	32(30)	5(5)	5.40	51%
Dazl	Deleted i	33605	898	31(25)	10(10)	3.51	32%
Ighv1-47	Immunog	11190	790	32(24)	6(4)	5.53	79%
	Ig heavy	13040	746	23(19)	7(6)	7.20	75%
Hspa8	Uncharac	71041	738	32(22)	17(14)	1.47	28%
Igkv4-55	Immunog	12842	679	10(10)	2(2)	0.60	27%
Ybx3	Y-box-b	38790	674	36(18)	14(8)	1.90	54%
	A6 anti-[	10974	619	55(26)	12(6)	33.83	41%
Igh	Igh prote	51955	616	51(33)	7(6)	1.09	15%
Krt10	Keratin, t	57178	568	23(16)	8(6)	0.65	12%
Ighv1-43	Immunog	10868	560	19(17)	5(4)	2.97	59%
Ighv1-18	Immunog	13018	503	23(15)	5(4)	4.14	56%
Ighv1-42	Immunog	10883	502	17(16)	6(5)	4.23	79%
Krt1	Keratin, t	66079	498	25(18)	7(6)	0.55	7%

Ybx2	Y box pr(	38058	494	30(21)	15(13)	3.13	43%
	Ig kappa	12017	489	14(12)	4(3)	2.52	53%
Igkv12-4	Immunog	12725	487	13(11)	3(3)	2.28	40%
	Ig kappa	12087	479	12(11)	4(4)	2.49	68%
Vim	Vimentin	53698	478	28(21)	16(13)	1.59	42%
Krt17	Keratin, t	48417	472	29(21)	18(13)	1.69	30%
Ighv1-82	Immunog	10757	466	31(19)	6(6)	20.35	84%
	Ig kappa	12149	461	10(10)	4(4)	2.49	68%
Igh	Igh prote	52966	460	52(29)	6(4)	0.72	11%
Tubb5	Tubulin b	50095	446	24(18)	13(12)	1.44	30%
Tubb4b	Tubulin b	50255	444	27(18)	14(12)	1.59	34%
Krt14	Keratin, t	53176	441	28(17)	15(10)	0.94	21%
	IgA heav	9845	441	13(13)	3(3)	2.34	34%
Ighv1-22	Immunog	13127	437	17(13)	4(3)	1.53	50%
Ighv1-62	Immunog	11332	426	19(13)	5(3)	2.77	77%
	Anti-HIV	13246	415	18(15)	4(4)	2.98	52%
	IgA heav	12907	407	18(9)	4(3)	1.03	27%
	Light cha	11273	398	23(14)	7(4)	3.92	50%
Krt42	Keratin, t	50444	390	31(19)	16(11)	1.28	25%
	Anti-HIV	12332	389	15(11)	4(3)	1.67	39%
Wgn-scF	Single-cl	25976	370	14(10)	5(4)	1.33	23%
Tuba1c	Tubulin a	50592	360	17(12)	9(8)	0.88	26%
Adib	Adiponec	29650	359	16(16)	5(5)	1.90	22%
scFv	ScFv 6H8	26188	355	15(9)	6(3)	0.62	33%
Igh-V718	B9-scFv	29591	350	17(13)	6(4)	0.70	23%
Krt6a	Keratin, t	59641	349	22(12)	14(9)	0.81	20%
C4b	C4a anap	194437	343	17(14)	11(9)	0.22	10%
Ighv10-3	Immunog	13707	340	14(7)	5(3)	2.05	47%
	Anti-HIV	13344	323	9(8)	3(3)	1.49	39%
Ighv6-3	Immunog	13422	322	13(10)	6(5)	3.89	49%
	Type I ep	10712	320	12(9)	6(5)	6.02	43%
Upf1	Regulato	125200	320	20(13)	17(11)	0.40	16%
Tuba1b	Tubulin a	50804	316	16(11)	9(8)	0.87	26%
Hspa9	Stress-7	73701	314	11(8)	8(6)	0.30	17%
Hnrnpm	Uncharac	86893	311	15(11)	10(8)	0.40	14%
Krt8	Keratin, t	54531	309	11(10)	4(3)	0.26	4%
Hnrnpu	B30.2/SP	77152	306	10(6)	5(3)	0.18	7%
Ighv6-6	Immunog	13456	301	8(7)	5(4)	3.89	47%
Ighv4-1	Immunog	13037	292	16(11)	7(7)	7.20	68%
Ighv8-12	Immunog	13369	291	9(6)	3(3)	1.49	31%
	Ig kappa	12071	285	5(5)	2(2)	1.12	30%
	Anti-hum	25325	285	23(11)	6(4)	1.10	40%
Igkv5-39	Immunog	10453	284	7(7)	2(2)	1.36	31%

Gm10881	Ig kappa	12721	279	6(6)	2(2)	1.04	23%
	Anti-HIV	13140	269	12(6)	2(2)	0.59	31%
	Ig heavy	12781	259	10(7)	5(3)	3.16	40%
	IgM heav	11306	255	17(11)	3(3)	7.38	60%
Grn	Uncharac	68298	242	9(7)	7(5)	0.26	18%
Actb	Actin, be	42052	240	20(13)	11(9)	1.30	41%
Igkv9-12	Immunog	10527	239	5(4)	2(1)	0.76	42%
Krt78	Krt78 prc	55415	235	9(4)	3(2)	0.12	5%
Igkv4-61	Immunog	10261	234	8(7)	1(1)	2.22	19%
Iglv2	LOC2076	25201	233	22(11)	6(4)	1.11	40%
Ighv5-16	Immunog	13380	231	10(8)	4(4)	2.90	38%
Igkv6-23	Immunog	12893	228	23(16)	3(3)	1.03	33%
VH186.2-	VH186.2-	16149	227	16(12)	3(3)	2.81	28%
Gapdh	Glyceralc	38914	226	9(6)	6(4)	0.50	23%
	Ig heavy	12753	222	10(5)	5(3)	2.28	40%
Gm5478	Predictec	58112	220	14(8)	9(6)	0.47	12%
Alb	Serum al	70700	219	12(7)	9(6)	0.31	17%
Hist1h2bj	Histone I	13570	218	6(4)	4(2)	2.08	35%
VH186.2-	V304-D-	15937	218	8(8)	2(2)	1.62	23%
Ighv1-76	Immunog	10986	217	15(8)	6(4)	5.76	69%
	IgM heav	11533	214	14(9)	3(3)	2.69	57%
	IgG1 hea	11042	209	21(11)	6(6)	10.68	76%
Igkv5-43	Immunog	12706	207	7(7)	2(2)	1.05	23%
Igkv8-27	Immunog	11083	207	21(15)	2(2)	0.72	21%
Igkv6-17	Immunog	12866	206	9(8)	4(4)	2.25	40%
Iglv1	Light cha	10669	205	7(5)	2(2)	2.08	56%
Iglc2	IgL2 (Fra	11419	205	24(9)	7(4)	9.69	77%
	AberrantI	12738	204	23(16)	4(3)	1.04	43%
V165-D-	V165-D-	15866	201	9(6)	2(2)	0.79	30%
Ighv7-3	IgA heav	12574	190	8(6)	4(4)	1.63	37%
Adia	Adiponec	30026	183	13(8)	5(5)	0.69	19%
Igkv2-13	Immunog	13294	181	5(4)	3(2)	0.98	43%
Hnrnpc	RRM dor	36997	179	9(4)	4(3)	0.41	12%
Lamb2	Laminin s	203579	176	16(8)	14(6)	0.15	9%
Ighv5-12	Immunog	13238	173	10(8)	4(3)	1.51	36%
Col6a3	Collagen	289998	173	12(5)	12(5)	0.06	5%
	Ig heavy	10768	169	16(7)	4(2)	1.30	38%
Ighv5-15	Immunog	13106	167	8(5)	3(2)	0.59	32%
	IgM heav	12606	164	15(5)	4(2)	3.21	58%
Ddx5	DEAD bo	69750	163	10(6)	7(5)	0.32	12%
Krt77	Keratin, t	61379	161	7(5)	4(2)	0.30	6%
Hspg2	Basemer	479445	159	15(6)	14(6)	0.04	4%
Igkv12-4	Immunog	12720	159	5(5)	3(3)	1.04	27%

Hnrnph1	Heteroge	51470	156	12(7)	7(5)	0.45	16%
Hist2h4	Histone I	11360	156	10(4)	6(3)	1.89	50%
Ighv9-3	Immunog	13101	153	7(4)	4(4)	1.53	40%
Cert1	Ceramid	68921	149	13(6)	9(5)	0.39	16%
Hnrnpk	Heteroge	48760	148	11(7)	7(4)	0.58	17%
Ighv2-2	Immunog	12798	147	6(4)	3(3)	1.59	47%
Trim21	Uncharac	54318	143	11(4)	9(4)	0.42	21%
Igkv5-37	Immunog	12712	140	8(3)	3(2)	1.04	20%
Col4a1	Collagen	161719	138	10(5)	5(3)	0.11	4%
Atr	Non-spe	304134	138	19(9)	17(8)	0.09	6%
Igkv4-53	Immunog	10474	138	4(4)	2(2)	2.15	41%
Ighv1-75	Immunog	13119	137	15(5)	1(1)	1.01	20%
Slc25a3	Phospha	40044	137	4(3)	4(3)	0.37	10%
Igkv8-28	Immunog	11010	136	5(5)	2(2)	1.27	23%
Igkv2-10	Immunog	13163	135	2(2)	1(1)	0.26	10%
Dvl1	Segment	75825	135	11(6)	9(6)	0.35	18%
Hnrnpf	Heteroge	46043	135	7(6)	3(3)	0.32	7%
Ighv9-4	Immunog	11041	133	9(4)	5(2)	1.98	60%
	Ig kappa	12137	132	4(3)	2(2)	0.65	20%
Ddx3x	RNA heli	73455	131	4(4)	4(4)	0.19	6%
Dhx9	DEAH bo	150836	127	15(10)	12(8)	0.21	9%
scFv	ScFv B8E	27656	125	9(6)	5(4)	0.58	22%
Ighm	Immunog	50708	122	4(4)	4(4)	0.29	11%
C3	Complem	187905	122	15(9)	11(7)	0.13	7%
Rps8	40S ribos	24533	121	8(4)	6(4)	0.67	37%
	B cell an	14944	120	4(4)	2(2)	0.51	15%
Sfpq	Splicing	75508	120	6(6)	6(6)	0.29	9%
Atxn2	LsmAD d	102271	120	5(4)	4(3)	0.13	7%
Igkv4-80	Immunog	12668	117	2(1)	1(1)	0.61	13%
C1s1	Uncharac	77975	114	3(3)	3(3)	0.13	6%
Piwil1	Piwi-like	99424	114	7(2)	7(2)	0.14	10%
Lamc1	Laminin s	182776	114	7(2)	7(2)	0.05	5%
H2ac4	Histone I	14127	112	3(3)	2(2)	0.91	13%
Ighv5-12	Immunog	13212	112	3(3)	2(2)	0.58	18%
	IgM heav	12075	111	9(3)	2(2)	1.12	36%
Rpl7	Uncharac	31388	111	8(5)	6(4)	0.50	16%
Igh	Igh prote	53563	109	4(4)	2(2)	0.20	4%
Ighv1-39	Immunog	13036	109	5(4)	2(2)	0.60	29%
Col4a3	Collagen	163278	108	9(4)	4(3)	0.08	4%
V303-D-	V303-D-	15866	107	10(5)	2(2)	1.17	30%
Slc25a31	ADP/ATF	35521	107	8(5)	7(4)	0.56	20%
Dynll1	Dynein li	10500	106	4(4)	2(2)	1.34	37%
Elavl1	ELAV-lik	34145	106	7(4)	6(3)	0.32	25%

C1qa	Complem	26186	104	5(4)	2(2)	0.43	10%
	VH gene	15412	103	9(3)	4(3)	0.82	22%
Ighv1-67	Immunog	11083	102	6(2)	2(1)	0.31	32%
Ighv1-78	Immunog	11154	102	6(2)	2(2)	0.72	35%
Rps5	40S ribo:	20572	100	5(2)	4(2)	0.35	14%
Rpl3	Uncharac	46378	99	3(3)	3(3)	0.23	6%
	Ig heavy	13081	98	4(3)	3(3)	1.01	27%
Gm8797	Predicte	8723	98	6(5)	4(3)	2.83	51%
Igkv6-15	Immunog	12863	98	6(4)	3(3)	1.03	33%
VH186.2-	VH186.2-	15875	97	8(3)	2(2)	0.47	18%
Boll	Protein b	31103	97	7(3)	4(3)	0.36	15%
Ighv1-85	Immunog	13117	96	4(4)	2(2)	1.01	26%
Cyp17a1	Uncharac	57928	95	6(4)	6(4)	0.25	15%
	B2 18-5	13334	93	2(2)	1(1)	0.26	15%
Ilf2	DZF dom	43211	93	5(3)	4(2)	0.16	15%
Rps14	Uncharac	16507	93	2(2)	2(2)	0.45	15%
Igkv16-1(	Immunog	13037	92	4(1)	2(1)	0.60	23%
Col18a1	Collagen	183210	92	2(1)	2(1)	0.04	1%
	IgA heav	12215	91	4(2)	3(2)	0.64	26%
Rplp0	60S acid	16160	89	5(3)	4(3)	0.77	30%
Rps25	40S ribo:	10360	89	6(4)	2(2)	0.78	20%
Mov10	RNA heli	121766	87	10(4)	9(4)	0.14	10%
Rps7	40S ribo:	22113	85	6(3)	5(3)	0.53	30%
Atp5f1a	ATP synt	59830	84	8(3)	6(3)	0.31	12%
Ywhaq	14-3-3 p	28046	84	2(1)	2(1)	0.12	8%
Igkv4-9(	Immunog	12715	82	2(2)	1(1)	0.61	13%
	V(H) regi	12583	81	6(3)	2(2)	1.06	21%
Ighv1-5	Immunog	10953	81	4(3)	1(1)	0.73	19%
Igkv6-13	Immunog	10548	81	6(4)	5(4)	2.11	64%
	IgM heav	12410	80	6(3)	3(3)	1.08	21%
Ighv8-11	Immunog	13188	80	3(2)	1(1)	0.26	13%
	Anti-VIP	12008	79	9(4)	4(3)	1.13	41%
Igkv14-1(	Immunog	12813	78	4(2)	1(1)	0.27	13%
Cct3	T-compl	58811	77	7(2)	6(2)	0.24	12%
	IgA heav	12263	76	1(1)	1(1)	0.28	14%
Fcgr1	High affi	45259	76	8(3)	5(3)	0.33	14%
Col6a1	Collagen	109562	75	4(2)	4(2)	0.06	3%
Pcbp2	Poly(rC)-	19973	74	3(2)	3(2)	0.37	20%
Dsp	Desmopl	263538	74	8(2)	7(2)	0.04	3%
Rpl8	60S ribo:	28277	74	5(2)	4(1)	0.25	11%
Serpina3	Serine pr	46871	72	4(2)	3(1)	0.15	5%
Nadk2	NAD kina	39281	71	1(1)	1(1)	0.08	4%
Cltc	Uncharac	115368	71	2(1)	2(1)	0.03	1%

Ighv7-1	Immunog	13807	70	2(2)	2(2)	0.56	16%
Rpl18	60S ribos	18004	70	5(3)	5(3)	0.68	26%
Jup	Junction	82490	69	3(2)	3(2)	0.08	3%
Rps9	40S ribos	22635	68	11(3)	5(2)	0.32	24%
Rps19	40S ribos	15353	68	3(2)	3(2)	0.49	21%
Sfxn3	Siderofle	12543	66	3(1)	3(1)	0.27	30%
Igkv9-12	Immunog	12978	65	1(1)	1(1)	0.26	13%
Rpl27	60S ribos	10498	65	2(2)	1(1)	0.33	10%
Rpl4	60S ribos	47409	65	6(2)	5(2)	0.14	16%
Atp2a3	Calcium-	108900	64	1(1)	1(1)	0.03	1%
Ighv5-4	Immunog	13128	64	4(3)	3(2)	1.01	24%
Dlst	Dihydroli	49306	62	2(2)	1(1)	0.07	1%
Rpl23a	Rpl23a p	17553	62	2(2)	2(2)	0.42	13%
Fn1	Fibronec	266327	62	6(2)	6(2)	0.02	2%
Prkra	Interfero	34806	60	3(2)	3(2)	0.20	12%
	IgM heav	11911	59	7(3)	2(2)	1.14	34%
Rpl15	Ribosom	24245	59	4(2)	4(2)	0.30	16%
Fnbp1	Formin-b	71634	59	4(2)	4(2)	0.20	6%
Gm17190	Predicted	38159	59	5(1)	2(1)	0.18	8%
Rpl13	60S ribos	24639	58	2(1)	2(1)	0.14	10%
	Pterin-m	13131	58	4(3)	4(3)	1.01	40%
Igkv4-91	Immunog	12830	56	1(1)	1(1)	0.27	29%
Rbmxl2	RNA-bin	42115	56	3(2)	3(2)	0.16	9%
Ighv1-12	Immunog	10885	55	10(2)	3(1)	1.29	61%
Mrps34	28S ribos	25868	51	3(2)	2(2)	0.27	6%
Ncbp1	Nuclear c	92951	51	3(2)	3(2)	0.07	5%
Col4a2	Collagen	168417	51	5(3)	3(2)	0.06	2%
Rpl14	60S ribos	23663	51	3(2)	3(2)	0.30	13%
Ighv8-4	Immunog	10905	51	1(1)	1(1)	0.32	14%
Tgm2	Protein-g	78153	51	4(1)	4(1)	0.04	6%
Rpl19	Ribosom	23347	50	1(1)	1(1)	0.14	4%
Eef1a1	Elongatio	50414	50	4(1)	4(1)	0.07	12%
Ighv1-9	Immunog	13037	49	2(2)	1(1)	0.60	21%
Rpl7a	60S ribos	30057	49	3(1)	3(1)	0.23	13%
Ppfibp1	Uncharac	109098	49	2(1)	2(1)	0.03	2%
	IgM heav	12303	48	6(2)	3(2)	0.63	25%
	Imunoglo	14188	48	1(1)	1(1)	0.24	12%
Nid2	Nidogen-	131181	48	3(2)	3(2)	0.05	3%
Hsp90aa	Hsp90aa	66081	48	3(2)	2(2)	0.10	2%
Gna13	Uncharac	44353	48	2(1)	2(1)	0.07	7%
Dynll2	Dynein li	10457	47	2(2)	2(2)	0.77	20%
Rps16	Rps16 pr	16549	47	1(1)	1(1)	0.20	6%
Fbn1	Fibrillin-	126518	47	2(2)	2(2)	0.05	1%

Rps4l	40S ribo:	29394	46	4(1)	4(1)	0.24	16%
Gmpr2	GMP red	38507	46	4(1)	4(1)	0.18	13%
Igkv3-9	Immunog	13023	46	1(1)	1(1)	0.26	9%
	V(Kappa)	16813	46	4(2)	2(2)	0.44	11%
Ighv13-2	Immunog	11450	45	1(1)	1(1)	0.30	14%
Fgg	Fibrinoge	50044	45	2(1)	2(1)	0.07	4%
Rpl24	TRASH d	18355	45	3(2)	2(2)	0.40	10%
	Anti-myc	12158	44	2(1)	1(1)	0.28	21%
Hnrnpul1	Heteroge	96513	44	4(2)	4(2)	0.07	6%
Hnrnpd	Heteroge	24878	43	4(1)	3(1)	0.13	14%
Scml2	Scm poly	90264	42	2(1)	2(1)	0.04	2%
Snrpb	Small nu	8608	42	1(1)	1(1)	0.40	10%
Ighv7-4	Immunog	13615	42	3(2)	3(2)	0.56	28%
Cct8	CCT-the	53391	42	2(1)	2(1)	0.06	3%
Hspa4l	Heat sho	95178	42	3(1)	3(1)	0.03	3%
Ighv14-4	Immunog	12241	41	2(1)	2(1)	0.28	39%
	B cell an	16615	41	1(1)	1(1)	0.20	20%
FAM120A	Constitut	122766	40	2(1)	2(1)	0.03	1%
Pdia6	Protein d	49026	40	1(1)	1(1)	0.07	3%
Bhmt2	S-methy	40416	40	3(1)	3(1)	0.08	6%
Rpl36a-p	Ribosoma	12732	40	1(1)	1(1)	0.27	8%
Mtch2	Mitochor	32951	39	1(1)	1(1)	0.10	2%
Cyp11a1	Choleste	60447	39	2(1)	2(1)	0.05	4%
Hnrnpul2	Heteroge	85515	39	8(1)	7(1)	0.04	8%
Rpl36	60S ribo:	12346	39	1(1)	1(1)	0.28	12%
Ighv8-8	Immunog	13291	39	5(2)	3(2)	0.58	12%
C1rb	Complem	81367	39	17(1)	6(1)	0.04	7%
Slc22a6	Solute ca	60715	39	2(1)	1(1)	0.05	1%
Igkv17-12	Immunog	12493	38	2(1)	1(1)	0.27	14%
Pura	Uncharac	35186	38	3(1)	1(1)	0.09	2%
Raly	RNA-bin	23163	38	3(2)	3(2)	0.31	12%
Dhx15	Pre-mRN	69219	38	2(1)	2(1)	0.10	4%
Fus	RNA-bin	14228	38	3(1)	3(1)	0.54	33%
Snx21	Sorting n	40455	37	1(1)	1(1)	0.08	1%
	IgA heav	12944	37	2(2)	2(2)	0.60	15%
Rps12	40S ribo:	16367	37	1(1)	1(1)	0.21	6%
Hsd17b11	Estradiol	33145	37	2(1)	2(1)	0.10	6%
Pi4k2b	Phospha	54014	36	1(1)	1(1)	0.06	1%
Slc25a20	Solute ca	33286	36	1(1)	1(1)	0.10	2%
Ighv1-66	Immunog	13084	36	1(1)	1(1)	0.26	17%
Hnrnpl	Heteroge	51160	36	2(1)	2(1)	0.06	6%
Hbat1	Alpha-gl	15133	35	2(2)	2(2)	0.50	15%
Ighv1-11	Immunog	13027	35	2(1)	1(1)	0.26	10%

Mettl3	N6-aden	65274	35	3(1)	1(1)	0.05	1%
Osbpl3	Oxystero	101346	35	1(1)	1(1)	0.03	0%
Tardbp	TAR DNA	26983	35	1(1)	1(1)	0.12	7%
Atp5f1b	ATP synt	56265	35	4(3)	4(3)	0.19	8%
Ilf3	Interleuk	97791	34	4(1)	4(1)	0.07	4%
Rps18	40S ribo	17736	34	5(1)	3(1)	0.19	17%
	IgG1 hea	12557	34	5(2)	2(1)	0.27	13%
H3c14	Histone H	20348	34	7(1)	5(1)	0.36	19%
Rpl18a	60S ribo	12920	34	2(1)	2(1)	0.27	16%
Brsk2	BR serin	76033	34	1(1)	1(1)	0.04	1%
IGHG2	IgG2 (Fra	36418	33	4(1)	4(1)	0.09	11%
Fga	Fibrinoge	88117	33	2(1)	2(1)	0.04	2%
Igkv8-30	CC49 Fa	14690	32	1(1)	1(1)	0.23	9%
	Elastin (F	67277	32	1(1)	1(1)	0.05	1%
Syncrip	Heteroge	62733	32	1(1)	1(1)	0.05	2%
Rpl21	60S ribo	18607	32	2(1)	2(1)	0.18	16%
G2e3	G2/M ph	83613	31	1(1)	1(1)	0.04	1%
Slc2a3	MFS dom	53957	31	2(1)	2(1)	0.06	5%
9530053	RIKEN cD	292361	31	2(1)	1(1)	0.01	0%
Vcp	Uncharac	80669	30	4(1)	4(1)	0.04	7%
Mocs3	Adenylylt	50313	30	4(1)	2(1)	0.07	3%
Clu	Clusterin	26366	30	1(1)	1(1)	0.13	5%
Idh1	Isocitrate	21211	30	1(1)	1(1)	0.16	4%
Larp4	La-relate	74204	30	2(1)	2(1)	0.04	1%
Mrps24	28S ribo	19174	29	1(1)	1(1)	0.18	4%
Stau1	Staufen (	48823	29	2(1)	2(1)	0.07	4%
Slc4a2	Anion ex	137284	29	1(0)	1(0)	0.02	0%
Cdk20	Cyclin-d	21093	29	9(0)	2(0)	0.16	10%
Igkv4-86	Immunog	12441	28	1(0)	1(0)	0.28	12%
Rps20	40S ribo	13478	28	2(1)	1(1)	0.25	10%
Gm839	Gene mo	14462	28	1(0)	1(0)	0.24	4%
Col4a4	Collagen	166046	28	3(0)	3(0)	0.02	3%
Rpl6	60S ribo	33546	28	4(2)	3(2)	0.21	10%
Ccdc190	Coiled-c	32743	27	1(1)	1(1)	0.10	4%
Col6a2	Collagen	100010	27	2(0)	2(0)	0.03	1%
Snrpe	Small nu	6269	27	1(1)	1(1)	0.58	21%
Hnrnpdl	Heteroge	46413	27	1(0)	1(0)	0.07	1%
Rps15a	40S ribo	8158	27	3(0)	2(0)	0.43	22%
Bag2	BAG fam	23630	26	3(1)	3(1)	0.30	11%
Myo9b	Unconve	223928	26	1(0)	1(0)	0.01	0%
Stambp	MPN dom	49207	26	1(0)	1(0)	0.07	1%
Mcm4	DNA heli	97288	26	3(1)	2(1)	0.03	1%
Tex13c2	TEX13 fa	59404	26	1(1)	1(1)	0.06	1%

Hmgcs2	3-hydrox	57334	25	2(0)	1(0)	0.06	1%
Cct5	T-comple	21569	25	1(0)	1(0)	0.16	5%
Btbd1	BTB (PO:	53812	25	5(1)	1(1)	0.06	1%
Rps3a1	40S ribos	30036	25	2(0)	2(0)	0.11	8%
Ccdc38	Coiled-c	65908	24	1(1)	1(1)	0.05	1%
Slc25a1	Tricarbox	34252	24	4(0)	3(0)	0.10	7%
Tdrd1	Tudor do	132064	24	2(1)	2(1)	0.05	2%
IgG2a	Anti-MO	18510	23	1(0)	1(0)	0.18	11%
Fxr1	Fragile X	15791	23	1(0)	1(0)	0.22	6%
Cct4	T-comple	58561	21	4(0)	3(0)	0.06	6%
Acsbg1	Long-cha	81230	21	2(0)	2(0)	0.04	3%
Cad	CAD prot	245650	20	2(1)	2(1)	0.01	0%
Ctnnd1	Catenin (	99337	18	3(1)	2(1)	0.03	3%
Rps13	40S ribos	5926	18	1(0)	1(0)	0.61	13%
Sf3b2	SAP dom	98242	17	4(0)	1(0)	0.03	0%
Cux2	Homeobo	155424	17	5(0)	2(0)	0.02	1%
Trim35	E3 ubiqu	59998	15	1(0)	1(0)	0.05	1%
Disp2	Protein d	150073	13	5(1)	2(1)	0.02	1%

DAZL_IP2_E8KO

Gene Na	Descripti	Mass	Score	Matches	Sequenc	emPAI	Coverage
HC	MAb 106	51959	3594	187(128)	24(21)	12.23	46%
Oplah	5-oxopro	138950	3423	161(119)	51(42)	4.33	44%
Oplah	Uncharac	92528	2418	111(85)	32(28)	4.89	43%
Ighg	Ighg prot	52711	2335	153(100)	24(20)	9.00	44%
Igh	Igh prote	52873	2237	180(106)	19(16)	11.68	40%
	Uncharac	52407	2070	181(108)	20(19)	16.57	47%
HC	MAb 44E	52527	2017	177(103)	20(18)	13.55	43%
HC	MAb 31C	51376	1810	110(67)	16(13)	2.92	50%
	Uncharac	26104	1665	48(41)	10(9)	5.09	44%
	Anti-colc	26780	1532	61(47)	14(12)	13.92	62%
Igkc	If kappa	24435	1427	63(47)	15(13)	12.02	73%
	Ig gamm	37086	1423	117(71)	12(11)	9.98	31%
Igh	Igh prote	52684	1314	97(56)	18(15)	3.03	53%
Igk	Igk prote	26570	1308	53(41)	13(11)	7.43	56%
	Anti-H5N	51702	1277	93(52)	14(12)	2.44	42%
	Anti-VIPa	12060	1195	21(19)	4(4)	4.75	51%
LC	MAb 110	26858	1174	43(36)	10(9)	4.79	42%
Igk	Igk prote	25971	1170	45(38)	11(10)	6.81	52%
	Fab4201	24356	1170	43(35)	10(9)	5.91	45%
LC	MAb 106	26605	1145	46(36)	11(10)	6.43	53%
Igk	Igk prote	26086	1129	45(37)	11(10)	5.87	45%
LC	MAb 6H1	26372	1120	44(35)	11(9)	5.71	44%
Igkv8-3	ENSMUS	26934	1119	52(39)	10(9)	4.75	40%
LOC100	LOC1000	26313	1066	41(34)	10(9)	5.00	48%
Igh	Igh prote	51659	1014	87(47)	12(10)	2.05	36%
Pabpc1	Polyaden	70824	949	63(40)	31(24)	2.89	47%
	Anti-dec	25885	890	15(14)	4(3)	0.83	27%
Igkv4-5	Immunog	12842	870	13(12)	2(2)	1.03	27%
Krt10	Keratin, t	57178	753	38(27)	11(8)	0.85	18%
Igh	Igh prote	52950	743	47(32)	8(6)	1.19	21%
Ighv1-18	Immunog	13018	708	27(22)	5(4)	5.49	56%
Vim	Vimentin	53712	678	39(24)	23(16)	2.49	46%
Dazl	Deleted i	33605	653	21(19)	8(7)	2.09	32%
	Ig heavy	13040	649	19(16)	6(5)	7.20	72%
Ighv1-4	Immunog	11190	640	23(16)	5(3)	2.82	68%
Krt1	Keratin, t	66079	585	29(24)	9(8)	0.62	8%
Krt14	Keratin, t	53176	583	47(25)	22(11)	1.62	30%
Krt42	Keratin, t	50444	581	44(25)	17(11)	1.76	28%
	IgM heav	11495	571	20(16)	4(4)	5.21	44%
Igh	Igh prote	51955	555	42(27)	9(7)	1.22	25%
Krt6a	Keratin, t	59641	525	23(17)	14(10)	0.81	23%

Krt17	Keratin, t	48417	514	39(24)	18(11)	1.87	23%
Tuba1c	Tubulin a	50592	493	22(18)	10(9)	1.42	29%
Ighv1-2	Immunog	13127	486	16(15)	3(3)	3.02	33%
Hspa8	Uncharac	71041	480	25(18)	17(13)	1.26	28%
	A6 anti-[	10974	478	60(17)	15(5)	14.35	45%
Ighv1-4	Immunog	10883	477	17(12)	5(5)	5.89	79%
Igkv5-3	Immunog	10453	473	10(8)	3(2)	1.36	47%
Tuba1b	Tubulin a	50804	473	21(17)	10(9)	1.41	29%
Tubb5	Tubulin b	50095	469	28(20)	16(12)	1.77	43%
	Type I ep	10712	463	16(10)	6(4)	6.02	43%
	Ig kappa	12017	456	22(16)	4(4)	4.83	53%
	Ig kappa	12087	450	13(11)	5(5)	4.75	90%
	IgA heav	9845	431	13(12)	3(3)	2.34	34%
Igh	Igh prote	51805	430	38(24)	6(5)	0.97	18%
Iglc2	IgL2 (Fra	11419	421	36(19)	7(4)	7.22	77%
Wgn-sc	Single-c	25976	420	16(11)	4(4)	1.07	23%
Ybx3	Y-box-b	38790	419	22(11)	11(6)	1.27	41%
	Anti-HIV	13246	411	15(15)	4(4)	2.98	52%
Tubb4b	Tubulin b	50255	407	30(20)	17(13)	2.14	47%
Iglv2	LOC207	25201	379	24(15)	5(4)	1.39	36%
Actb	Actin, be	42052	377	27(21)	15(13)	2.35	55%
Ighv1-6	Immunog	11332	374	13(10)	3(3)	1.89	43%
Igh	Igh prote	52966	372	41(22)	9(5)	0.83	23%
Adib	Adiponec	29650	371	20(17)	6(5)	2.22	26%
	Anti-VIP	12269	359	16(8)	3(3)	2.41	37%
	CH2-dor	12509	355	25(16)	5(4)	7.79	38%
	Anti-hum	25325	354	25(15)	5(4)	1.38	36%
Hspa9	Stress-7	73701	350	16(12)	11(7)	0.48	20%
Ybx2	Y box pr	38058	345	19(11)	12(9)	1.30	38%
Ighv6-3	Immunog	13422	342	13(10)	6(5)	3.89	49%
Alb	Serum al	70700	341	13(10)	8(7)	0.37	16%
Ighv1-8	Immunog	10757	340	33(17)	6(4)	8.27	84%
Igkv4-6	Immunog	10261	321	9(8)	2(2)	2.22	30%
Igkv9-1	Immunog	10527	313	6(5)	3(2)	1.34	55%
Ighv1-1	Immunog	13058	310	12(11)	3(3)	2.19	33%
Igkv5-4	Immunog	12706	310	8(6)	2(2)	1.05	23%
Ighv4-1	Immunog	13037	307	16(10)	8(7)	5.49	74%
Hnrnpu	B30.2/SI	77152	297	11(8)	7(5)	0.28	11%
Gm108	Ig kappa	12721	297	7(6)	3(3)	1.04	28%
Tubb3	Tubulin b	50842	276	18(11)	12(9)	1.00	31%
	Anti-HIV	12332	276	13(8)	4(3)	1.09	39%
Hnrnpm	Uncharac	86893	267	13(10)	11(9)	0.45	17%
Krt8	Keratin, t	54531	262	16(11)	7(4)	0.34	9%

Ighv6-6	Immunog	13456	260	7(7)	4(4)	2.11	39%
Hnrnpc	RRM dor	36997	257	10(10)	5(5)	0.54	16%
Iglv1	Light cha	10669	256	11(8)	3(3)	4.40	65%
	Immmun	13699	254	12(7)	5(2)	0.95	54%
Igkv1-13	Immunog	13411	244	11(8)	6(5)	2.90	45%
Krt78	Krt78 pro	55415	241	10(5)	2(1)	0.06	4%
Hnrnpk	Heteroge	48760	235	11(7)	7(4)	0.48	18%
Ighv1-78	Immunog	11154	233	12(9)	3(2)	2.87	51%
	IgA heav	12907	226	12(5)	4(3)	1.03	27%
Ighv1-76	Immunog	10986	225	14(9)	5(4)	2.92	69%
VH186.2	VH186.2-	16149	225	15(10)	4(3)	1.60	49%
Ighv5-16	Immunog	13380	214	9(8)	4(3)	1.48	38%
Igkv12-4	Immunog	12725	208	8(7)	3(3)	1.59	40%
Hnrnph1	Heteroge	51470	208	10(8)	6(6)	0.45	14%
Igkv4-8	Immunog	12668	197	2(2)	1(1)	0.27	13%
Upf1	Regulato	125200	196	19(12)	16(9)	0.29	16%
Krt79	Keratin, t	57802	196	12(8)	6(5)	0.32	8%
Ighv10-3	Immunog	13707	191	11(4)	6(3)	0.95	60%
Ighv7-1	Immunog	13807	190	5(5)	4(4)	1.43	38%
Grn	Uncharac	68298	189	9(7)	7(5)	0.33	18%
Adia	Adiponec	30026	187	8(6)	4(4)	0.52	17%
scFv	ScFv 6H8	26188	182	12(6)	5(4)	0.82	27%
Ighv1-39	Immunog	13036	178	5(5)	2(2)	1.55	29%
Ighv7-3	IgA heav	12574	171	7(7)	5(5)	2.35	49%
C1qa	Complem	26186	165	9(4)	4(2)	0.82	20%
	Anti-HIV	13140	164	14(4)	2(2)	0.59	31%
Rps3	40S ribos	26828	163	10(5)	8(3)	0.42	34%
C1s1	Uncharac	77975	160	8(4)	7(4)	0.23	14%
	Anti-myc	11046	156	14(9)	5(5)	4.07	57%
Ighv5-15	Immunog	13106	154	6(5)	3(2)	0.59	32%
	IgG1 hea	11042	153	22(8)	6(4)	4.15	76%
Gapdh	Glyceralc	38914	153	11(6)	8(5)	0.50	30%
V165-D	V165-D-	15866	150	10(6)	2(2)	0.79	30%
Igkv2-10	Immunog	13163	148	2(2)	1(1)	0.26	10%
Ddx5	DEAD bo	69750	148	9(5)	7(4)	0.26	12%
Igkv8-2	Immunog	11083	147	15(9)	3(3)	1.95	39%
Ighv9-4	Immunog	11041	143	9(7)	4(3)	2.92	48%
Ighv7-4	Immunog	13615	143	7(6)	5(4)	2.05	46%
Lig3	DNA liga	107665	141	7(3)	7(3)	0.13	7%
Ighv2-2	Immunog	12798	138	7(5)	3(3)	1.04	47%
C1ra	Complem	81504	138	22(4)	10(4)	0.17	16%
Ighv8-12	Immunog	13369	133	3(3)	2(2)	0.58	18%
Gstm1	Glutathic	28709	131	13(3)	9(3)	0.73	42%

Dynll1	Dynein li	10500	130	4(4)	2(2)	1.34	37%
Trim21	E3 ubiqu	54320	130	7(4)	6(3)	0.27	17%
Igkv6-2	Immunog	12893	128	16(8)	3(3)	1.03	33%
Ighv1-12	Immunog	10885	127	6(4)	3(3)	1.29	59%
Ighv8-8	Immunog	13291	127	6(4)	4(3)	0.98	26%
Ighv1-75	Immunog	13119	127	11(6)	1(1)	1.53	20%
Serpina3	Serine pr	46871	127	6(3)	4(2)	0.31	11%
Igkv8-19	Immunog	11223	122	15(7)	3(2)	0.71	41%
Dhx9	DEAH bo	150836	121	17(8)	12(8)	0.19	9%
Atxn2	LsmAD d	102271	118	4(2)	3(1)	0.03	5%
VH186.2	V304-D-	15937	117	11(5)	3(2)	0.78	41%
Slc25a4	ADP/ATP	33111	117	5(5)	4(4)	0.61	12%
Rps5	40S ribos	20572	116	4(2)	3(1)	0.35	13%
Rps25	40S ribos	10360	116	5(4)	2(2)	0.78	20%
	Aberrantl	12738	115	21(11)	4(4)	2.28	43%
Rpl18	Ribosoma	21687	113	7(4)	5(4)	0.78	20%
Igkv2-13	Immunog	13294	111	4(3)	2(1)	0.58	20%
Slc25a3	Phospha	40044	106	3(2)	3(2)	0.27	8%
C4b	C4a ana	194437	105	9(4)	7(4)	0.10	6%
Igkv6-17	Immunog	12866	105	10(7)	4(4)	2.25	40%
Tgm2	Protein-g	78153	103	4(3)	4(3)	0.18	7%
Hnrnpul	Heteroge	96513	101	4(3)	4(3)	0.11	6%
	IgM heav	12410	100	8(4)	3(2)	0.63	21%
Fcgr1	High affi	45259	99	6(5)	3(3)	0.24	7%
Hnrnpa3	Heteroge	37291	99	3(2)	3(2)	0.19	14%
Hnrnpf	Heteroge	46043	98	6(2)	5(2)	0.23	11%
Col4a1	Collagen	161719	98	5(3)	3(2)	0.08	2%
	Ig heavy	10768	98	9(5)	4(2)	1.30	38%
	IgM heav	11964	98	12(7)	4(3)	1.74	53%
Atp5f1a	ATP synt	59830	94	9(3)	7(3)	0.24	14%
	IgG1 hea	10514	93	10(4)	5(3)	1.34	56%
Igkv4-6	Immunog	12858	93	3(2)	1(1)	0.27	17%
scFv	ScFv B8E	27656	92	10(4)	6(4)	0.58	22%
H3c14	Histone H	20348	91	7(4)	5(3)	0.85	16%
Hsp90al	Heat sho	83571	91	6(5)	5(4)	0.17	7%
	IgA heav	12447	91	3(3)	2(2)	1.08	16%
Ighv13-2	Immunog	11450	91	7(3)	4(3)	1.20	59%
Fnbp1	Formin-b	71634	89	9(5)	7(4)	0.25	15%
Ywhaq	14-3-3 p	28046	89	1(1)	1(1)	0.12	5%
Ighv9-3	Immunog	13101	88	4(2)	2(2)	0.59	30%
Rps8	40S ribos	24533	86	5(3)	3(2)	0.29	20%
V303-D	V303-D-	15866	81	9(2)	2(2)	0.79	30%
Hist2h4	Histone H	11360	77	8(2)	5(2)	0.70	41%

	Ig kappa	12137	76	3(1)	2(1)	0.65	29%
Rpl19	Ribosom:	23347	76	5(3)	5(3)	0.50	23%
	Anti-CD:	26437	75	3(3)	3(3)	0.43	12%
Cct3	T-compl	61162	74	5(2)	5(2)	0.11	8%
Gmpr2	GMP red	38507	74	7(2)	6(2)	0.18	16%
Ighv2-9	Immunog	12697	74	3(3)	2(2)	0.61	18%
Jup	Junction	82490	74	4(3)	4(3)	0.12	4%
Krt72	Keratin,	57228	73	4(3)	3(2)	0.18	4%
Rps19	40S ribo:	15353	73	3(3)	3(3)	0.82	22%
	IgM heav	9665	72	3(2)	2(2)	0.85	28%
Igkv14-	Immunog	12813	72	4(4)	1(1)	0.61	13%
Aldh1a1	Retinal d	55060	71	3(2)	3(2)	0.19	7%
Hbbt1	Beta-glo	15928	71	1(1)	1(1)	0.21	8%
Cyp17a1	Uncharac	57928	71	7(5)	6(5)	0.39	15%
Rps9	40S ribo:	16334	71	9(2)	4(1)	0.21	26%
Pfkfb3	6-phosp	58054	70	4(1)	3(1)	0.06	5%
Igkv4-9	Immunog	12715	70	3(2)	1(1)	0.27	13%
Mov10	RNA heli	121766	69	6(3)	6(3)	0.08	8%
	B2 18-5	13334	69	1(1)	1(1)	0.26	15%
Igkv6-1:	Immunog	10548	69	7(4)	5(4)	2.11	57%
Col6a3	Collagen	289998	68	5(2)	5(2)	0.02	1%
Ass1	Argininos	46813	67	5(1)	5(1)	0.07	12%
Myh11	Myosin-1	224074	67	7(2)	4(2)	0.03	2%
Gmpr	GMP red	37971	67	5(2)	5(2)	0.18	18%
Rps10	40S ribo:	12763	67	2(2)	2(2)	0.61	14%
Rpl23a	Rpl23a p	17553	66	1(1)	1(1)	0.19	8%
Rpl15	Ribosom:	24245	66	5(3)	5(3)	0.47	17%
Ptpn23	Tyrosine-	185956	65	1(1)	1(1)	0.02	0%
Rpl13	60S ribo:	24639	65	2(2)	2(2)	0.29	10%
Sfpq	Splicing	75508	63	6(4)	6(4)	0.24	9%
Acsbg1	Long-ch:	81230	63	3(2)	3(2)	0.08	4%
Rpl3	Uncharac	46378	62	4(2)	3(2)	0.15	5%
Hsd3b1	Uncharac	42492	62	2(1)	2(1)	0.16	7%
Ighv1-8	Immunog	13117	59	3(2)	2(2)	0.59	26%
Col4a3	Collagen	163278	59	5(2)	3(2)	0.06	2%
Ywhaz	14-3-3 p	19167	59	1(1)	1(1)	0.18	8%
Prkra	Interfero	34806	58	4(1)	4(1)	0.10	16%
Ighv14-	Immunog	13154	57	3(2)	2(2)	0.58	21%
Ighv5-4	Immunog	13128	57	3(2)	3(2)	0.59	24%
	Anti-VIP:	12008	57	6(1)	3(1)	0.29	40%
Cyp11a1	Choleste	60447	56	5(1)	4(1)	0.11	8%
Rpl14	60S ribo:	23663	56	2(2)	2(2)	0.30	10%
Rps14	40S ribo:	12874	56	1(1)	1(1)	0.27	9%

Rpl8	60S ribo:	28277	55	3(2)	3(2)	0.25	13%
Mettl3	N6-aden	65260	55	7(4)	2(1)	0.10	3%
Igkv8-2	Immunog	11010	55	2(2)	2(2)	0.73	23%
Rplp0	60S acid	16160	55	5(2)	4(2)	1.14	34%
Slc25a1	Tricarbo›	34252	54	4(2)	4(2)	0.32	12%
Xrcc1	Unchara	69111	54	5(1)	4(1)	0.10	6%
Raly	RNA-bin	23163	53	3(3)	3(3)	0.50	12%
Ilf2	DZF dom	43211	53	2(1)	2(1)	0.08	8%
Ugt1a5	UDP-glu	60718	53	2(2)	2(2)	0.11	3%
Ldhb	L-lactate	37611	53	5(1)	4(1)	0.18	10%
Boll	Protein b	31103	53	4(1)	2(1)	0.11	6%
Rbm14	Unchara	66257	52	1(1)	1(1)	0.05	1%
Rpl4	60S ribo:	47409	52	6(2)	5(2)	0.14	19%
Pspc1	Paraspec	6997	52	1(1)	1(1)	0.51	28%
Igkv4-5	Immunog	10474	51	3(1)	2(1)	0.77	41%
Dlst	Dihydroli	49306	50	1(1)	1(1)	0.07	1%
Bhmt2	S-methy	40416	50	2(2)	1(1)	0.08	2%
Rpl23	60S ribo:	14970	50	2(1)	2(1)	0.23	20%
Gm8797	Predicte	8723	50	2(1)	2(1)	0.96	28%
Ighv1-5	Immunog	10953	49	4(2)	2(1)	0.31	34%
Pkm	Pyruvate	20809	49	1(1)	1(1)	0.16	6%
Pcbp2	Poly(rC)-	19973	49	3(1)	2(1)	0.17	14%
Hspg2	Basemer	479445	49	6(2)	6(2)	0.02	1%
Rps4l	40S ribo:	29394	49	3(1)	3(1)	0.11	11%
Cct8	T-compl	26082	48	1(1)	1(1)	0.13	5%
Osbpl3	Oxystero	101346	48	2(2)	1(1)	0.03	0%
	IgA heav	12263	48	1(1)	1(1)	0.28	14%
Rpl31	60S ribo:	14987	48	1(1)	1(1)	0.23	6%
Ighv1-58	Immunog	13100	48	1(1)	1(1)	0.26	12%
Rps7	40S ribo:	22113	46	4(1)	3(1)	0.15	12%
Rps16	Rps16 pr	16549	46	1(1)	1(1)	0.20	6%
Hsd17b1	Estradiol	33145	45	2(1)	2(1)	0.10	6%
Col18a1	Collagen	183210	45	2(2)	2(2)	0.04	2%
Vcp	Unchara	80669	44	4(1)	4(1)	0.04	6%
Atp1a1	Sodium/	114222	44	1(1)	1(1)	0.03	1%
Gm1719	Predicte	38159	43	2(1)	1(1)	0.18	4%
Hbat1	Alpha-gl	15133	43	2(2)	2(2)	0.50	15%
RP23-4	Ribosom	25044	43	1(1)	1(1)	0.13	5%
Sfxn3	Siderofle	31217	43	4(1)	4(1)	0.11	16%
Ighv1-9	Immunog	13037	43	3(2)	1(1)	0.60	21%
Rps3a1	40S ribo:	30036	42	2(1)	2(1)	0.11	9%
Rpl7a	60S ribo:	30057	42	2(1)	2(1)	0.11	8%
Hnrnpul	Heteroge	85515	42	7(3)	6(3)	0.12	7%

Rpl27	60S ribo	10498	42	1(1)	1(1)	0.33	10%
Rpl24	TRASH d	18355	41	2(2)	2(2)	0.40	13%
Snrpd2	Small nu	13632	41	1(1)	1(1)	0.25	8%
Matr3	Matr3 pr	95099	41	5(2)	4(2)	0.11	5%
Ighv1-11	Immunog	13027	41	3(2)	1(1)	0.26	10%
Hspd1	60 kDa h	61088	41	3(1)	3(1)	0.11	6%
Insrr	Insulin re	147291	41	3(1)	3(1)	0.02	1%
Rps13	40S ribo	17212	40	2(1)	2(1)	0.43	12%
Dsp	Desmopl	335158	40	7(2)	7(2)	0.02	2%
Rps15a	40S ribo	14944	40	4(1)	4(1)	0.51	25%
Igkv17-1	Immunog	12493	40	3(2)	1(1)	0.62	14%
H2bc9	Histone I	13912	38	4(2)	4(2)	0.55	28%
Rpl21	60S ribo	18636	38	1(1)	1(1)	0.18	9%
H2ac4	Histone I	14127	38	2(1)	2(1)	0.24	21%
Rpl12	60S ribo	17935	38	7(1)	4(1)	0.19	33%
Atp5f1b	ATP synt	56265	38	6(1)	4(1)	0.06	11%
Hsp90b	Uncharac	71099	37	2(2)	2(2)	0.09	4%
Rpsa	Laminin	32944	37	2(1)	2(1)	0.10	6%
Igkv8-2	Immunog	13426	37	1(1)	1(1)	0.25	9%
Snx21	Sorting n	40455	37	2(1)	2(1)	0.08	3%
Slc22a6	Solute ca	60715	37	1(1)	1(1)	0.05	1%
Mrps34	28S ribo	25868	36	2(1)	1(1)	0.13	3%
Rps11	40S ribo	15302	36	2(1)	2(1)	0.22	11%
Hnrnpl	Heteroge	51160	36	4(1)	4(1)	0.06	10%
Acad8	Isobutyry	45861	36	1(1)	1(1)	0.07	3%
Eef1a1	Elongatio	50414	36	5(2)	5(2)	0.14	16%
Cct7	T-comple	55536	35	5(1)	4(1)	0.06	8%
IGHG2	IgG2 (Fra	36418	35	4(2)	4(2)	0.19	11%
Igkv4-9	Anti-myc	10885	35	1(1)	1(1)	0.32	10%
Rpl6	60S ribo	33546	35	5(3)	3(2)	0.33	8%
Igkv6-1	Immunog	12954	35	4(1)	2(1)	0.60	20%
Rps12	40S ribo	16367	35	1(1)	1(1)	0.21	6%
Rpl18a	60S ribo	17607	35	1(1)	1(1)	0.19	6%
Plec	Plectin 1	519862	34	9(1)	7(1)	0.01	1%
Hnrnpa2	Heteroge	37437	34	2(1)	2(1)	0.18	5%
	E8 variab	12971	34	9(1)	3(1)	0.26	26%
Rps2	40S ribo	31475	34	2(1)	2(1)	0.11	7%
Col4a4	Collagen	166046	34	1(1)	1(1)	0.02	0%
Ighv1-6	Immunog	13084	33	2(1)	2(1)	0.26	30%
	IgG1 hea	12557	32	3(1)	2(1)	0.27	13%
Gna13	Uncharac	44353	32	6(1)	3(1)	0.07	9%
Igkv12-3	Immunog	12591	32	1(1)	1(1)	0.27	13%
Rps26	40S ribo	13292	32	2(1)	2(1)	0.26	20%

Ighv1-3	Immunog	13175	31	4(1)	3(1)	0.58	30%
Larp4	La-relate	74204	31	2(2)	2(2)	0.09	1%
Rpl5	60S ribos	18214	31	1(1)	1(1)	0.19	8%
Lmna	Prelamin	74478	31	3(1)	3(1)	0.04	4%
Cdk20	Cyclin-d	21093	30	7(1)	2(1)	0.16	10%
Ruvbl1	RuvB-lik	50524	30	2(1)	2(1)	0.07	6%
Igkv8-2	Immunog	13983	30	1(1)	1(1)	0.24	7%
Pura	Uncharac	35186	30	2(1)	2(1)	0.09	9%
Snx25	Snx25 pr	70703	30	13(1)	5(1)	0.05	6%
Hnrnpd	Heteroge	24878	30	3(1)	2(1)	0.13	10%
	IgM heav	12364	30	6(1)	2(1)	0.28	28%
Hmgcs2	3-hydrox	57334	30	3(1)	2(1)	0.06	3%
Igkv4-8	Immunog	12441	30	1(1)	1(1)	0.28	12%
Lamb2	Laminin s	203579	29	9(0)	8(0)	0.03	5%
Med23	Mediator	27094	29	1(1)	1(1)	0.12	3%
Prdx1	Peroxired	19029	29	1(1)	1(1)	0.18	6%
Brsk2	BR serine	76033	29	1(1)	1(1)	0.04	1%
Rpl36a-	Ribosoma	12732	29	1(1)	1(1)	0.27	8%
Igkv4-7	Anti-MO	14630	29	3(1)	2(1)	0.23	14%
Btbd1	BTB (PO	53812	29	4(1)	2(1)	0.06	3%
Piwil1	Piwi-like	57077	29	2(1)	2(1)	0.06	4%
Rpl30	60S ribos	10621	28	1(1)	1(1)	0.32	12%
Elavl1	ELAV-lik	36298	28	5(0)	4(0)	0.09	20%
Fus	RNA-bin	14228	28	1(1)	1(1)	0.24	10%
953005	RIKEN cD	292361	28	3(0)	1(0)	0.01	0%
Dhx15	Pre-mRN	69219	28	1(0)	1(0)	0.05	2%
Stambp	MPN dom	49207	28	1(0)	1(0)	0.07	1%
Pgam1	Phospho	28928	28	1(1)	1(1)	0.11	4%
Bag2	BAG fam	23630	28	3(1)	3(1)	0.14	11%
Immt	MICOS c	75924	28	2(0)	2(0)	0.04	3%
FAM120	Constitut	122766	27	2(1)	2(1)	0.03	1%
Ddx6	Probable	54556	27	2(1)	2(1)	0.06	3%
Rsbn1l	Round sp	69179	27	3(2)	2(1)	0.05	3%
Myo9b	Unconve	223928	26	2(0)	2(0)	0.01	1%
Atp5o	ATP synt	12148	26	1(1)	1(1)	0.28	12%
Ccdc38	Coiled-c	65908	26	3(2)	2(1)	0.05	4%
	MRP4 OS	18081	26	1(0)	1(0)	0.19	7%
Grin3a	Glutamat	128715	25	5(2)	3(1)	0.03	1%
Eef2	Tr-type (	96164	24	1(0)	1(0)	0.03	0%
Ccdc25	Coiled-c	24578	21	5(0)	1(0)	0.14	4%
Igh	Igh prote	53843	21	1(0)	1(0)	0.06	4%
Scml2	Scm poly	90264	21	2(0)	2(0)	0.04	2%
Dsg1b	Desmogl	115408	20	2(0)	2(0)	0.03	2%

Igkv12–8	Immunog	12131	20	1(0)	1(0)	0.28	17%
Hap1	Huntingti	37413	19	1(0)	1(0)	0.09	4%
	Anti–myc	12158	18	2(0)	1(0)	0.28	21%
Sf3b2	SAP dom	98242	17	5(0)	2(0)	0.03	1%
Iglv3	Immunog	13579	16	1(0)	1(0)	0.25	18%

IgG_IP1

Gene Name	Descripti	Mass	Score	Matches	Sequenc	emPAI	Coverage
HC	MAb 106	51959	3445	203(134)	25(20)	8.15	48%
Krt8	Keratin, t	54531	3294	93(80)	15(15)	4.48	21%
Igh	Igh prote	52873	3149	208(122)	20(18)	11.68	41%
	Uncharac	52407	2917	202(120)	22(22)	15.53	52%
HC	MAb 44E	52527	2810	195(115)	21(19)	12.69	45%
	Anti-colc	26780	2729	120(83)	14(12)	25.85	61%
Igkc	If kappa	24435	2640	126(85)	18(13)	35.34	80%
	Uncharac	26104	2633	100(72)	10(8)	11.53	46%
Igk	Igk prote	26570	2614	121(85)	14(12)	20.76	58%
Igh	Igh prote	52950	2455	159(96)	14(13)	5.49	34%
Ighg	Ighg prot	52711	2411	179(113)	24(19)	6.85	43%
Igk	Igk prote	25971	2395	107(75)	11(9)	17.20	54%
Ighg	Ighg prot	52514	2369	183(110)	24(18)	6.01	41%
Igkv8-30	ENSMUS	26934	2342	119(79)	10(8)	10.57	42%
HC	MAb 31C	51376	2322	140(89)	16(14)	4.35	46%
LC	MAb 110	26858	2311	97(69)	10(8)	10.68	44%
	Fab4201	24356	2297	96(68)	10(8)	13.98	48%
Igk	Igk prote	26086	2263	102(71)	12(9)	13.14	54%
LC	MAb 106	26605	2258	101(70)	13(9)	12.40	62%
LC	MAb 6H1	26372	2223	101(69)	12(9)	12.69	50%
	Ig gamm	37086	2220	160(93)	13(13)	13.19	34%
Ighg	Ig gamm	36936	2003	147(89)	13(12)	10.06	41%
Igh-3	Ig gamm	44972	1993	139(87)	22(16)	5.78	45%
Igh	Igh prote	51955	1897	139(79)	15(13)	4.95	37%
Igh	Igh prote	52966	1892	147(82)	17(12)	4.76	41%
Igh	Igh prote	51805	1842	133(78)	12(11)	4.62	31%
Igh	Igh prote	52380	1792	133(77)	12(11)	3.88	30%
	Anti-H5N	51702	1715	117(70)	15(12)	3.68	42%
Igh	Igh prote	52684	1625	115(68)	16(13)	3.55	44%
Ighv1-81	Immunog	13094	1156	40(32)	5(5)	5.40	51%
Igh	Igh prote	51659	1018	99(56)	13(10)	2.45	37%
	Anti-VIPa	12060	989	22(14)	4(3)	4.75	51%
Actb	Actin, be	42052	920	55(30)	17(12)	3.90	60%
	Ig heavy	13040	863	40(30)	6(6)	9.37	72%
Krt10	Keratin, t	57178	810	35(25)	11(9)	0.85	15%
	CH2 dom	12416	793	69(47)	7(7)	22.72	51%
Ighv1-34	Immunog	13137	790	35(26)	4(4)	9.18	39%
Krt18	Keratin, t	47509	755	66(36)	9(8)	1.09	28%
Ighv1-22	Immunog	13127	730	30(21)	3(3)	3.02	33%
	Anti-dec	25885	702	14(11)	5(3)	0.83	34%
	IgA heav	9845	696	28(20)	3(3)	3.51	34%

Ighv1-42	Immunog	10883	694	27(21)	5(4)	5.89	64%
Krt1	Keratin, t	66079	679	34(27)	7(7)	0.62	5%
Igkv9-124	Immunog	10527	671	17(14)	5(4)	6.29	57%
Ighv1-43	Immunog	10868	631	27(19)	6(4)	4.23	59%
Ighv1-18	Immunog	13018	629	26(20)	5(5)	9.37	56%
	A6 anti-[	10974	615	74(30)	19(7)	102.84	45%
	Ig kappa	12017	606	30(19)	5(5)	4.83	54%
Ighv1-20	Immunog	13176	596	24(16)	4(3)	2.98	37%
Ighv1-82	Immunog	10757	575	43(28)	6(6)	20.35	84%
	IgM heav	12403	572	22(15)	5(3)	2.38	52%
	Ig kappa	12087	525	13(13)	3(3)	1.72	40%
	Anti-HIV	13344	520	18(13)	3(3)	2.94	39%
	Ig kappa	12072	517	24(15)	5(5)	3.48	54%
Igkv1-135	Immunog	13411	515	21(17)	7(6)	3.89	45%
Igkv4-55	Immunog	12842	503	7(7)	2(2)	0.60	27%
	Fab4201	25716	492	20(13)	4(4)	0.84	27%
Ighv1-76	Immunog	10986	479	20(15)	6(6)	19.17	84%
Krt14	Keratin, t	53176	467	26(19)	13(9)	0.94	26%
	Heat sho	69966	446	23(16)	16(11)	0.90	31%
Igkv1-133	Immunog	13343	445	17(15)	6(5)	2.94	45%
Ccdc33	Coiled-c	82970	435	33(21)	20(15)	1.26	30%
Krt13	Keratin, t	48066	431	25(15)	9(8)	1.08	15%
Ighv1-47	Immunog	11190	421	15(12)	5(2)	1.24	64%
	CH2-dor	12509	419	45(25)	8(6)	10.20	62%
Igkv5-39	Immunog	10453	413	11(9)	2(2)	2.15	31%
Ighv1-62-2	Immunog	11332	413	16(16)	4(4)	3.92	62%
Igkv8-27	Immunog	11083	402	31(19)	3(3)	1.95	39%
Ighv10-3	Immunog	13707	401	18(10)	5(3)	2.05	44%
Igh	Igh prote	52404	398	14(13)	7(7)	0.73	16%
Wgn-scFv	Single-c	25976	389	19(16)	6(4)	1.33	23%
Igkv4-61	Immunog	10261	385	14(12)	2(2)	6.73	30%
Ighv5-16	Immunog	13380	384	14(12)	4(4)	2.90	38%
Igkv12-46	Immunog	12725	382	16(12)	3(3)	2.28	40%
Krt42	Keratin, t	50444	381	22(16)	10(7)	0.77	22%
Alb	Serum al	70700	355	16(11)	11(7)	0.57	20%
	Ig heavy	13074	352	16(13)	7(7)	7.07	55%
Myh9	Myosin-9	227429	350	19(9)	8(4)	0.09	6%
	Anti-VIPa	12269	342	24(8)	4(3)	3.37	38%
Igkv5-43	Immunog	12706	342	11(7)	2(2)	1.05	23%
Ighv6-3	Immunog	13422	338	17(10)	6(5)	5.13	47%
	VH regio	11239	337	16(13)	4(3)	1.24	38%
	Immmuno	15105	336	12(8)	3(3)	1.76	48%
	Anti-HIV	12332	320	13(10)	4(3)	1.09	39%

Igkv5-45	Immunog	12824	315	8(6)	2(2)	0.60	23%
Ighv4-1	Immunog	13037	313	17(14)	8(8)	12.10	74%
Ighv1-12	Immunog	10885	313	16(11)	4(4)	5.89	80%
Krt6a	Keratin, t	59641	312	21(14)	13(8)	0.71	19%
	Light cha	11273	305	20(10)	7(3)	2.77	53%
VH186.2-D-\	VH186.2-	16149	305	24(11)	3(3)	2.81	28%
	IgG1 hea	11042	295	28(16)	6(6)	10.68	76%
Igkv12-44	Immunog	12720	293	11(9)	4(3)	1.04	40%
	IgM heav	9665	287	14(11)	4(3)	7.61	50%
Tuba1b	Tubulin a	50804	275	18(9)	11(8)	0.87	33%
Gm10881	Ig kappa	12721	274	4(4)	2(2)	0.61	23%
Ighv7-1	Immunog	13807	272	8(8)	5(5)	2.03	50%
VH186.2-D-\	V304-D-	15937	270	14(9)	2(2)	2.17	23%
	Ig kappa	12137	266	8(6)	4(3)	1.12	43%
	Aberrant	12738	265	40(19)	5(5)	3.16	44%
Adib	Adiponec	29650	261	13(9)	5(4)	1.34	22%
Ighv8-12	Immunog	13369	255	6(6)	2(2)	0.58	18%
	Ig heavy	10768	250	15(12)	3(3)	3.02	30%
Igkv6-23	Immunog	12893	249	31(15)	3(3)	1.03	33%
Gm5478	Predicted	58112	247	13(8)	6(4)	0.25	8%
scFv	ScFv 6H8	26188	246	20(10)	4(4)	0.82	24%
	IgG1 hea	12343	243	16(11)	8(5)	3.37	40%
Igkv2-137	Immunog	13294	242	6(6)	2(2)	0.98	20%
Igkv8-19	Immunog	13478	234	29(16)	3(3)	1.48	29%
Ighv14-3	Immunog	13081	233	17(10)	3(3)	3.02	26%
Ighv9-4	Immunog	11041	222	12(8)	5(3)	2.92	60%
scFv	ScFv B8E	27656	222	14(9)	4(4)	0.98	19%
	E8 variab	12971	221	21(9)	4(4)	2.22	35%
Ighv1-69	Immunog	13054	221	10(7)	3(2)	2.19	55%
Ighv6-6	Immunog	13456	219	10(7)	4(3)	2.90	39%
Igkv4-51	Immunog	12945	218	3(3)	1(1)	0.26	13%
Igkv8-21	Immunog	13291	210	26(14)	2(2)	0.58	20%
	Type I ep	10712	209	7(6)	5(4)	2.04	43%
Igkv6-17	Immunog	12866	202	11(8)	3(3)	1.57	33%
Adia	Adiponec	30026	200	12(7)	5(4)	0.69	19%
Igkv8-28	Immunog	11010	198	6(5)	3(3)	1.27	38%
Igkv2-109	Immunog	13163	197	3(3)	1(1)	0.26	10%
Igkv4-80	Immunog	12668	196	3(2)	1(1)	0.61	13%
	Anti-hum	25325	195	23(7)	4(3)	0.64	34%
Ighv1-52	Immunog	13151	193	10(7)	3(2)	2.19	55%
Igkv4-53	Immunog	10474	192	5(4)	2(2)	2.15	41%
	Ig heavy	13883	190	6(6)	4(4)	1.41	44%
Ighv1-78	Immunog	11154	187	9(5)	2(2)	2.87	35%

Ighv1-19	Immunog	12962	186	7(5)	3(3)	2.22	50%
	Anti-myc	11046	185	20(11)	4(4)	1.95	50%
	Ig heavy	12797	183	8(6)	3(2)	1.59	29%
V165-D-J-C	V165-D-	15866	176	10(8)	2(2)	0.79	30%
	Anti-HIV	13140	176	10(4)	3(3)	1.53	39%
Ighv2-2	Immunog	12798	171	8(4)	2(2)	0.61	18%
Ighv5-12	Immunog	13238	167	8(6)	4(4)	2.16	32%
	Anti-CD:	26437	166	7(5)	5(3)	0.43	21%
Igkv16-104	Immunog	13037	162	3(2)	2(1)	0.26	23%
	Anti-HIV	12463	162	10(6)	4(2)	1.08	25%
Tubb4b	Tubulin b	50255	158	14(7)	8(4)	0.37	23%
Krt79	Keratin, t	57802	156	13(9)	7(5)	0.39	9%
Ighv7-3	IgA heav	12574	156	8(5)	3(3)	1.06	31%
Ighv1-67	Immunog	11083	149	9(4)	3(2)	0.72	43%
Ighv5-15	Immunog	13106	149	7(5)	3(3)	1.53	32%
Ighv1-75	Immunog	13119	146	10(4)	2(2)	1.01	33%
	IgA heav	12570	144	7(7)	2(2)	1.06	21%
Igkv6-15	Immunog	12863	143	7(5)	3(3)	1.57	33%
Iglv1	Light cha	10669	143	5(4)	2(2)	1.32	56%
	IgG1 hea	10514	143	11(6)	5(4)	3.13	56%
Ighv1-85	Immunog	13117	136	5(3)	2(2)	1.01	26%
Ighv8-8	Immunog	13291	133	7(5)	4(3)	1.49	27%
Iglc2	IgL2 (Fra	11419	132	8(4)	5(3)	1.20	74%
Grn	Uncharac	68298	132	7(6)	6(5)	0.26	15%
Ighv2-6	Immunog	12815	131	5(4)	2(2)	0.61	18%
Igh	Igh prote	54144	130	6(6)	2(2)	0.13	6%
	IgA heav	10055	128	5(5)	2(2)	1.42	24%
	McAB 0.'	13408	125	7(5)	4(2)	0.97	34%
Igkv6-29	Immunog	12685	123	6(5)	3(3)	1.05	34%
Igkv6-13	Immunog	10548	122	7(4)	4(4)	2.11	50%
Ighv5-4	Immunog	13128	122	7(5)	2(2)	1.53	18%
	B2 18-5	13334	121	3(2)	1(1)	0.26	15%
Krt76	Keratin, t	63319	118	10(7)	6(4)	0.22	7%
Ighv1-56	Immunog	10954	117	10(3)	2(1)	1.27	44%
Ighv9-3	Immunog	13101	117	6(3)	3(2)	0.59	40%
	Anti-lipo	13890	115	14(4)	4(1)	0.93	35%
	B cell an	16615	114	4(3)	2(2)	0.74	20%
C1s1	Uncharac	77975	113	4(4)	3(3)	0.13	4%
Igkv1-132	Immunog	13540	109	4(3)	3(3)	0.96	26%
	IgM heav	12303	108	14(4)	6(3)	1.09	40%
C1qa	Complem	26186	108	10(6)	5(2)	1.05	26%
Ighv1-39	Immunog	13036	107	4(3)	2(2)	1.55	29%
Ighv1-11	Immunog	13027	105	8(6)	3(3)	1.02	27%

VH186.2-D-…	VH186.2-	15875	102	18(4)	2(2)	0.79	18%
	IgG1 hea	12383	102	5(4)	2(2)	0.63	19%
Krt72	Keratin, t	57228	100	5(5)	3(3)	0.18	4%
Igkv4-63	Immunoc	10265	99	12(6)	3(3)	2.22	48%
Hspa9	Stress-7	73701	97	5(2)	5(2)	0.09	12%
Eef1a1	Elongatic	50414	94	5(2)	4(2)	0.14	12%
Hnrnpm	Unchara	86893	93	7(3)	6(3)	0.12	9%
Ighv2-9	Immunoc	12697	92	5(4)	2(2)	1.05	18%
	IgM heav	12386	91	6(3)	3(2)	1.08	30%
Gapdh	Glyceralc	38914	88	9(4)	7(4)	0.39	29%
Igkv6-14	Immunoc	12954	88	5(3)	3(3)	1.02	28%
	Fd (Fragr	5741	87	5(3)	2(2)	1.64	45%
	IgG1 hea	12575	85	4(2)	1(1)	0.27	18%
Dlat	Dihydroli	68469	84	7(3)	5(2)	0.15	10%
Ighv13-2	Immunoc	11450	83	5(2)	3(2)	0.69	41%
	IgM heav	12410	83	10(3)	3(3)	1.08	21%
Igkv5-37	Immunoc	12712	81	1(1)	1(1)	0.27	10%
	Anti-lox-	51928	81	4(4)	3(3)	0.20	9%
	Anti-HIV	12351	77	6(4)	4(2)	1.09	23%
Igkv8-24	Immunoc	13426	75	3(2)	2(2)	0.57	17%
	B cell an	14944	73	5(5)	2(2)	0.51	15%
C1ra	Complem	81504	72	12(1)	4(1)	0.04	5%
	Ig kappa	12148	72	4(1)	2(1)	0.28	38%
Ldhc	L-lactate	36231	70	4(2)	4(2)	0.19	10%
Igkv4-90	Immunoc	12715	69	2(2)	1(1)	0.27	13%
Ighv1-62-1	Immunoc	3210	68	6(2)	1(1)	3.82	63%
Hnrnpu	B30.2/SI	77152	67	1(1)	1(1)	0.04	2%
	Anti-lox-	26229	65	4(2)	4(2)	0.27	28%
Hist2h4	Histone I	11360	64	5(2)	3(2)	0.70	29%
Igkv4-81	Immunoc	10342	64	2(1)	1(1)	0.34	18%
	IgA heav	12447	64	4(3)	2(2)	0.63	19%
Igkv3-9	Immunoc	13023	64	2(1)	1(1)	0.26	9%
Igkv14-100	Immunoc	12813	64	2(2)	1(1)	0.27	13%
	Ig kappa	11734	63	2(2)	2(2)	0.67	29%
	Anti-hun	13085	63	2(2)	2(2)	0.59	16%
Slc25a31	ADP/ATF	35521	54	3(1)	3(1)	0.09	8%
Ighv1-5	Immunoc	10953	54	4(2)	1(1)	0.73	19%
Ybx2	Y-box-b	31419	53	3(1)	3(1)	0.11	15%
	Heavy ch	12158	52	3(2)	2(2)	0.64	14%
Igkv8-16	Immunoc	13615	52	1(1)	1(1)	0.25	10%
Igkv2-112	Immunoc	13386	51	3(1)	1(1)	0.25	13%
Vim	Vimentin	49220	49	5(1)	5(1)	0.14	13%
Igh	Igh prote	53315	49	2(1)	2(1)	0.06	4%

Lyz1	1,4-beta-	17240	49	2(1)	2(1)	0.20	12%
Tsks	Serine/tl	57915	48	2(1)	2(1)	0.06	7%
Igkv17-127	Immunog	12493	47	2(2)	1(1)	0.62	14%
Sfpq	Splicing	75508	46	5(1)	5(1)	0.04	7%
CU041261.1-	Novel me	20926	46	2(2)	1(1)	0.35	6%
Dsp	Desmopl	335158	44	3(1)	3(1)	0.01	0%
Trim21	E3 ubiqu	54320	44	7(1)	4(1)	0.13	8%
Osbpl3	Oxystero	101346	44	2(2)	1(1)	0.03	0%
Igkv8-26	Immunog	13983	44	1(1)	1(1)	0.24	7%
Igkv12-38	Immunog	12591	44	5(1)	4(1)	0.62	54%
Igkv14-130	Immunog	13207	42	1(1)	1(1)	0.26	11%
Ighv1-9	Immunog	13037	41	2(1)	1(1)	0.26	21%
Dlst	Dihydroli	49306	40	1(1)	1(1)	0.07	1%
Apol10b	Apolipop	37141	40	1(1)	1(1)	0.09	1%
Ighv3-3	Immunog	11404	39	1(1)	1(1)	0.30	16%
Pspc1	Paraspec	58835	39	2(1)	2(1)	0.06	4%
Serpina3h	Serpina3	45823	36	2(1)	2(1)	0.07	4%
Rps27a	40S ribos	18282	35	3(1)	3(1)	0.18	19%
Ighv1-58	Immunog	13100	35	1(1)	1(1)	0.26	12%
Hbbt1	Beta-glo	15928	35	1(1)	1(1)	0.21	8%
	VH regio	11299	34	3(2)	2(1)	0.30	18%
Igkv8-30	CC49 Fa	14690	34	1(1)	1(1)	0.23	9%
Rpl23a	Rpl23a p	17553	34	1(1)	1(1)	0.19	8%
Mettl3	N6-aden	65274	33	3(1)	1(1)	0.05	1%
IGHV	Immunog	12969	33	2(1)	1(1)	0.26	10%
Gstm1	Glutathic	28709	33	3(1)	3(1)	0.12	14%
Hist1h2bj	Histone I	13570	33	3(2)	2(1)	0.25	19%
Pabpc2	Polyaden	70824	33	5(1)	5(1)	0.05	7%
	Anti-myc	11331	32	2(1)	2(1)	0.30	34%
Myl12b	Myosin r	19824	32	1(1)	1(1)	0.17	10%
9530053A07	RIKEN cD	292361	31	3(0)	1(0)	0.01	0%
Slc2a3	MFS don	53957	31	2(1)	1(1)	0.06	1%
Btbd1	BTB (PO	53812	29	2(2)	1(1)	0.06	1%
Dnajb1	J domain	38314	29	3(1)	3(1)	0.09	8%
C3	Complem	187905	29	6(2)	6(2)	0.04	2%
Cnot9	CCR4-N	33922	28	1(1)	1(1)	0.10	4%
Pdia6	Protein d	49026	27	1(0)	1(0)	0.07	3%
	IgA heav	12416	27	1(1)	1(1)	0.28	8%
Rsbn1l	Round sp	8450	26	2(1)	1(1)	0.42	9%
Nf1	Neurofib	323097	26	3(0)	2(0)	0.01	0%
Hsp90aa1	Hsp90aa	66081	26	4(1)	4(1)	0.10	6%
Stmn2	Uncharac	24150	25	2(0)	1(0)	0.14	3%
Brsk2	BR serin	76033	24	1(0)	1(0)	0.04	1%

Myo9b	Unconve	223928	24	3(0)	3(0)	0.01	2%
Pdha2	Pyruvate	44196	23	1(0)	1(0)	0.07	2%
Hbat1	Alpha-gl	15133	22	2(0)	2(0)	0.23	15%
Ighv1-49	Immunog	10976	22	2(0)	1(0)	0.31	17%
Opa3	Optic atr	24158	19	4(0)	2(0)	0.14	8%
Tceanc	Transcrip	41407	17	1(0)	1(0)	0.08	4%
Fyttd1	UAP56-i	35923	14	2(0)	2(0)	0.09	8%

IgG_IP2

Gene Na	Descripti	Mass	Score	Matches	Sequenc	emPAI	Coverage
Igkc	If kappa	24435	5025	206(145)	20(15)	52.40	84%
Igk	Igk prote	26570	4883	210(149)	18(15)	33.94	66%
	Uncharac	26104	4796	176(130)	12(10)	14.95	51%
HC	MAb 106	51959	4783	267(181)	28(22)	12.23	47%
Ighg2b	Immunog	37284	4767	268(181)	29(22)	34.63	64%
	Anti-colc	26780	4748	198(142)	16(14)	37.19	66%
Igk	Igk prote	25971	4688	189(138)	13(11)	22.18	58%
	Uncharac	26625	4590	179(132)	14(11)	20.49	62%
	IgE L cha	26561	4528	183(133)	16(12)	18.33	64%
Igkv8-30	ENSMUS	26934	4379	199(146)	12(10)	13.60	47%
LC	MAb 110	26858	4235	170(124)	12(10)	13.76	48%
	Fab4201	24356	4226	169(123)	12(10)	18.38	53%
LC	MAb 6H1	26372	4220	175(128)	14(12)	18.56	55%
Igk	Igk prote	26086	4218	177(128)	15(12)	19.29	58%
LC	MAb 106	26605	4178	172(126)	14(12)	18.10	63%
LC	MAb 31C	26238	4149	169(123)	12(10)	16.78	58%
LOC1000	LOC1000	26313	4104	168(121)	12(9)	14.59	50%
Igh	Igh prote	52873	3854	257(164)	20(16)	18.37	41%
Ighg	Ighg prot	52711	3556	240(160)	28(22)	11.75	46%
	Uncharac	52407	3419	253(161)	22(20)	29.42	52%
HC	MAb 44E	52527	3379	249(155)	21(18)	19.97	45%
Ighg	Ighg prot	52514	3355	239(151)	27(20)	8.50	41%
Igh	Igh prote	52950	3149	191(120)	15(12)	6.33	36%
HC	MAb 31C	51376	2903	168(118)	16(13)	5.06	46%
Igh-3	Ig gamm	44972	2712	177(115)	24(18)	7.38	43%
Ighg	Ig gamm	36936	2710	182(117)	13(12)	14.59	44%
	Ig gamm	37086	2659	194(125)	13(12)	24.84	34%
Igh	Igh prote	52966	2631	183(109)	17(13)	6.33	43%
Igh	Igh prote	51955	2605	176(106)	17(13)	5.73	43%
Igh	Igh prote	51805	2505	167(103)	12(11)	5.35	33%
Igh	Igh prote	52380	2497	168(104)	12(11)	4.52	32%
	Anti-H5N	51702	2407	152(105)	15(13)	4.99	42%
Igh	Igh prote	52684	2358	154(106)	17(14)	5.16	47%
Igh	Igh prote	51659	1849	137(90)	13(11)	3.42	37%
	Anti-VIPa	12060	1511	24(24)	4(4)	6.38	51%
	CH2 dom	12416	1137	86(61)	6(6)	48.27	44%
	Anti-dec	25885	1062	21(16)	5(3)	1.34	36%
Ighv1-42	Immunog	10883	981	36(27)	6(4)	8.07	86%
	Ig heavy	13040	980	41(28)	8(5)	9.37	73%
Ighv1-31	Immunog	11073	946	41(28)	6(4)	13.97	66%
Krt10	Keratin, t	57178	911	41(29)	13(10)	1.19	16%

Ighv1-81	Immunog	13094	894	32(25)	5(4)	3.02	51%
Ighv1-43	Immunog	10868	875	36(26)	6(5)	10.95	66%
	Kappa ch	12375	869	22(19)	5(4)	3.37	47%
	Ig kappa	12017	863	44(29)	5(5)	8.66	54%
Ighv1-22	Immunog	13127	853	29(24)	3(3)	4.07	33%
Ighv1-34	Immunog	13137	814	33(23)	5(3)	5.40	39%
	Ig kappa	12087	805	22(18)	5(5)	4.75	90%
Igkv4-55	Immunog	12842	805	13(12)	2(2)	0.60	27%
Ighv1-20	Immunog	13176	802	28(20)	5(3)	5.31	43%
	IgM heav	12403	788	26(21)	5(4)	4.50	58%
Igkv12-4	Immunog	12725	774	26(21)	3(3)	2.28	40%
	IgA heav	9845	761	24(21)	3(3)	3.51	34%
Igkv9-12	Immunog	10527	760	25(19)	6(6)	11.85	57%
	A6 anti-[	10974	740	92(35)	16(7)	59.14	41%
Igkv1-135	Immunog	13411	722	40(27)	8(8)	8.64	45%
Krt1	Keratin, t	66079	721	34(28)	9(7)	0.71	9%
	Ig kappa	12072	714	35(21)	5(5)	6.38	54%
	Ig heavy	15619	684	17(16)	3(3)	0.80	27%
	Pterin-m	12153	630	16(13)	4(3)	1.72	73%
	Anti-HIV	13246	625	28(21)	5(4)	2.98	58%
	B2 12-1 a	11751	614	13(11)	3(2)	2.60	37%
Igkv1-133	Immunog	13343	607	30(22)	7(7)	5.22	45%
Igkv4-61	Immunog	10261	606	18(16)	2(2)	6.73	30%
Ighv1-18	Immunog	13018	604	28(17)	6(4)	9.37	56%
	Anti-HIV	12332	598	18(15)	4(3)	1.67	39%
	Anti-VIPa	12269	578	26(16)	4(4)	4.58	38%
Ighv1-82	Immunog	10757	574	39(24)	6(6)	27.21	84%
Ccdc33	Coiled-c	82970	572	42(23)	26(18)	1.35	38%
	Fab4201	25716	572	29(17)	4(4)	1.08	27%
scFv	ScFv 6H8	26188	564	38(18)	6(6)	1.61	33%
Igkv8-27	Immunog	11083	558	41(31)	3(3)	1.95	39%
	Light cha	11273	557	28(17)	10(4)	2.77	49%
Ighv1-12	Immunog	10885	550	23(17)	5(4)	19.75	87%
	CH2-dor	12509	546	53(35)	7(6)	17.15	55%
Gm10881	Ig kappa	12721	525	10(10)	2(2)	1.04	23%
Igkv8-19	Immunog	11223	519	42(33)	4(4)	4.00	53%
	Aberrantl	12738	516	52(34)	5(5)	3.16	44%
Igkv4-74	Immunog	12997	506	10(10)	2(2)	1.02	36%
Wgn-scF	Single-c	25976	504	21(15)	7(5)	1.63	26%
Krt8	Keratin, t	54531	481	25(19)	7(4)	0.51	8%
	Immmun	13699	477	20(12)	6(3)	0.95	57%
Krt42	Keratin, t	50444	475	30(20)	15(12)	1.43	32%
Ighv1-76	Immunog	10986	474	23(18)	7(6)	33.83	84%

Ighv1-47	Immunog	11190	471	21(15)	6(3)	2.82	79%
Igkv13-8	Immunog	12872	471	10(8)	2(2)	0.60	32%
Krt6a	Keratin, t	59641	457	25(17)	15(8)	0.71	22%
	Ig heavy	13074	443	22(16)	7(6)	5.40	55%
Igh	Igh prote	52404	442	17(14)	6(5)	0.44	11%
Ighv5-16	Immunog	13380	430	19(12)	4(3)	2.11	38%
	IgM heav	11306	423	20(14)	4(3)	7.38	67%
	IgM heav	11304	423	17(13)	4(3)	5.42	71%
Ighv1-53	V23-D-J	15784	420	24(16)	4(3)	3.76	45%
	VH regio	11239	418	16(12)	4(3)	1.92	38%
VH186.2-	VH186.2-	16149	409	31(15)	3(3)	2.81	28%
Igkv5-43	Immunog	12706	402	10(8)	2(2)	1.05	23%
Ighv1-62	Immunog	11332	401	20(16)	3(3)	2.77	43%
Krt17	Keratin, t	48417	395	28(19)	14(10)	1.21	20%
Ighv10-3	Immunog	13707	393	19(10)	5(3)	2.05	44%
Ighv6-3	Immunog	13422	392	19(12)	7(5)	5.13	55%
	IgG1 hea	12343	384	27(16)	10(5)	10.66	50%
Krt14	Keratin, t	53176	376	30(18)	16(11)	1.18	26%
Alb	Serum al	70700	376	23(12)	12(7)	0.50	24%
Ighv1-15	Immunog	13058	375	15(13)	3(3)	2.19	33%
Igkv6-23	Immunog	12893	374	39(28)	3(3)	1.57	33%
Krt77	Keratin, t	61379	371	19(14)	7(4)	0.52	7%
VH186.2-	V304-D-	15937	368	17(12)	2(2)	2.17	23%
Ighv6-6	Immunog	13456	364	15(11)	6(5)	5.13	57%
Adib	Adiponec	29650	363	15(11)	5(4)	1.61	22%
Igkv4-53	Immunog	10474	357	12(7)	2(2)	3.19	41%
Ighv4-1	Immunog	13037	356	21(15)	7(6)	5.49	55%
IGHVDJ	Immunog	11943	354	19(13)	5(3)	4.83	55%
	Ig heavy	10768	349	19(13)	3(3)	3.02	30%
Ighv9-4	Immunog	11041	349	17(13)	5(4)	4.15	60%
	Ig kappa	12137	348	11(10)	4(4)	1.72	43%
Ighv1-52	Immunog	13151	347	17(13)	4(3)	5.40	61%
Ighv8-12	Immunog	13369	344	8(7)	3(3)	0.98	31%
	IgM heav	9665	338	19(15)	5(4)	10.71	50%
Igkv16-10	Immunog	13037	337	7(6)	2(1)	0.60	23%
Iglv1	Light cha	10669	335	12(10)	3(3)	4.40	65%
Igkv5-45	Immunog	12824	330	6(6)	2(2)	0.60	23%
Krt73	Keratin, t	59502	330	16(13)	7(5)	0.46	11%
	IgG light	12446	328	34(26)	3(2)	0.63	33%
Igkv12-4	Immunog	12720	322	12(11)	4(4)	1.59	40%
	Anti-hum	25325	301	26(16)	5(5)	1.38	38%
	IgA heav	10055	297	11(11)	3(3)	4.88	45%
Igkv6-17	Immunog	12866	293	17(12)	4(4)	3.11	40%

Ighv9-1	Immunog	11196	290	12(11)	3(3)	1.92	34%
Ighv1-85	Immunog	13117	288	9(9)	2(2)	1.53	26%
	Anti-myc	11046	285	26(16)	5(5)	5.65	57%
Ighv14-3	Immunog	13081	285	20(13)	3(3)	3.02	26%
	IgA heav	10273	284	15(10)	3(2)	4.67	38%
	Heat sho	69966	281	18(12)	15(9)	0.58	29%
Igkv8-28	Immunog	11010	271	12(10)	4(4)	2.92	53%
Ighv1-19	Immunog	12962	270	9(7)	4(3)	3.07	56%
scFv	ScFv B8E	27656	266	17(12)	4(4)	0.77	19%
Igkv2-10	Immunog	13163	262	4(4)	1(1)	0.26	10%
C1qa	Complem	26186	260	10(9)	5(4)	1.05	26%
Grn	Uncharac	68298	255	11(10)	6(5)	0.39	15%
	IgG1 hea	11042	251	24(14)	6(6)	14.35	76%
Igkv4-80	Immunog	12668	248	5(5)	1(1)	0.61	13%
Igkv4-51	Immunog	12945	242	6(3)	2(1)	0.60	31%
	Anti-HIV	13140	240	12(7)	3(3)	1.53	39%
Ighv1-78	Immunog	11154	234	10(8)	2(2)	2.87	35%
Igkv6-13	Immunog	10548	234	14(9)	5(5)	6.29	57%
V165-D-	V165-D-	15866	227	10(8)	2(2)	0.79	30%
EP3-1VH	EP3-1 he	13588	226	11(7)	4(3)	2.08	41%
Igkv4-68	Immunog	12858	224	5(5)	1(1)	0.60	17%
	Anti-CD:	26437	215	11(10)	6(6)	1.04	24%
	Ig heavy	13883	211	6(5)	3(3)	0.93	32%
Ighv7-1	Immunog	13807	211	6(5)	4(4)	1.43	38%
	Ig heavy	13029	211	9(8)	3(3)	1.02	27%
Igkv2-13	Immunog	13294	208	6(5)	3(2)	0.98	43%
Ighv7-3	IgG1 hea	12200	204	9(7)	4(3)	1.69	45%
	IgA heav	10250	204	11(9)	3(3)	3.31	37%
Iglc2	IgL2 (Fra	11419	199	12(6)	4(3)	2.73	66%
	Ig heavy	12753	198	13(5)	5(2)	1.04	42%
C1sa	Complem	78347	197	7(5)	6(4)	0.18	11%
Ighv1-75	Immunog	13119	193	15(9)	4(2)	2.19	60%
Krt79	Keratin,	57802	192	19(10)	8(4)	0.39	10%
Ighv1-56	Immunog	10954	186	16(7)	2(1)	0.73	44%
Ighv5-12	Immunog	13238	185	7(6)	4(4)	2.16	32%
Igkv4-63	Immunog	10265	182	15(8)	4(3)	4.77	56%
	Ig heavy	12797	181	11(7)	4(3)	1.59	37%
	Anti-lox-	26229	180	9(4)	4(2)	0.43	29%
	E8 variab	12971	179	22(8)	5(4)	2.22	42%
Igkv6-15	Immunog	12863	179	9(6)	3(3)	1.57	33%
Adia	Adiponec	30026	176	12(7)	5(5)	0.88	19%
Actb	Actin, be	42052	175	22(12)	10(8)	1.13	31%
Ighv2-2	Immunog	12798	172	7(2)	2(1)	1.04	18%

	IgM heav	12606	171	17(9)	5(3)	3.21	73%
Ighv5-15	Immunog	13106	166	6(5)	3(3)	1.53	32%
Ighv1-39	Immunog	13036	164	4(4)	2(2)	1.02	29%
	B2 18-5	13334	163	8(4)	1(1)	0.26	15%
Ighv2-9	Immunog	12697	163	7(6)	2(2)	1.61	18%
	Anti-HIV	11864	162	14(8)	5(5)	4.92	51%
	IgM heav	12303	161	20(6)	5(3)	1.67	35%
	Ig heavy	13106	160	12(4)	4(2)	0.59	36%
Tubb4b	Tubulin b	50255	159	14(7)	10(5)	0.46	26%
	IgA heav	12570	158	10(6)	2(2)	1.06	21%
	Anti-lox-	51928	158	7(5)	5(3)	0.28	13%
	IgM heav	11964	156	15(8)	5(3)	4.83	53%
Trim21	E3 ubiqu	54320	154	13(7)	10(7)	0.60	20%
Igkv2-112	Immunog	13386	154	5(4)	1(1)	0.25	13%
	IgG1 hea	10514	153	11(6)	5(3)	2.11	56%
Igh	Igh prote	54144	152	9(6)	2(2)	0.19	6%
	Ig kappa	12068	152	26(11)	4(4)	2.49	35%
Tuba1c	Tubulin a	50592	151	13(9)	8(6)	0.56	27%
	IgM heav	12030	151	10(7)	3(3)	1.74	37%
	Anti-HIV	12463	147	8(6)	4(2)	0.63	42%
	IgG1 hea	12511	147	8(6)	3(3)	2.35	45%
Igkv1-132	Immunog	13540	147	6(6)	3(3)	0.96	26%
	IgG1 hea	12383	146	8(7)	2(2)	1.08	19%
Ighv1-67	Immunog	11083	142	8(3)	3(2)	0.72	43%
Krt35	Keratin, t	51809	140	5(5)	2(2)	0.13	3%
Krt76	Keratin, t	63319	140	15(9)	7(4)	0.29	9%
	Type I ep	10712	139	6(5)	5(4)	2.04	43%
Ighv8-8	Immunog	13291	134	7(5)	4(3)	1.49	27%
	IgA heav	9949	134	4(4)	2(2)	1.45	24%
	VH gene	15412	131	9(7)	3(3)	1.22	19%
	IgG1 hea	11715	130	8(5)	3(2)	0.67	29%
Ighv2-6	Immunog	12815	129	7(5)	2(2)	1.04	18%
	Pterin-m	13131	127	8(7)	3(3)	2.19	34%
Ighv1-54	Immunog	12856	125	6(5)	2(2)	1.57	33%
Igkv6-20	Immunog	12746	125	8(6)	4(3)	1.59	40%
Igkv8-24	Immunog	13426	123	5(4)	2(2)	0.57	17%
Gapdh	Glyceralc	38914	121	6(3)	5(2)	0.18	18%
Ighv4-2	IgM heav	11663	121	7(5)	4(3)	1.17	34%
	B cell an	14944	121	4(4)	2(2)	0.51	15%
Igkv5-37	Immunog	12712	120	3(2)	2(1)	0.27	17%
Igkv6-14	Immunog	12954	120	9(5)	3(3)	1.55	28%
Hspa9	Stress-7	73701	120	5(4)	5(4)	0.19	9%
Krt72	Keratin, t	57228	119	9(6)	4(3)	0.25	5%

	Ig kappa	11668	118	8(5)	4(2)	2.64	42%
Ighv1-66	Immunog	13084	117	6(3)	2(2)	0.59	30%
	Anti-HIV	13453	116	5(5)	2(2)	0.97	19%
IGLC2	IgL2 (Fra	10067	113	3(3)	2(2)	1.42	32%
	IgG1 hea	12507	112	9(4)	5(2)	1.06	37%
Ighv9-3	Immunog	13101	111	6(3)	3(3)	1.53	40%
	Ig kappa	11734	111	4(3)	2(2)	0.67	29%
Ighv5-4	Immunog	13128	110	7(4)	2(2)	1.53	18%
Dsp	Desmopl	335158	107	9(3)	8(2)	0.02	2%
Igkv9-12	Immunog	12978	107	4(2)	2(2)	0.60	26%
	IgG1 hea	11750	106	15(5)	4(3)	2.60	45%
Igkv4-90	Immunog	12715	105	2(2)	1(1)	0.61	13%
Ighv1-11	Immunog	13027	105	9(5)	5(3)	1.02	40%
Igkv12-3	Immunog	12591	98	7(5)	3(3)	1.63	37%
	V(Kappa)	16813	97	6(3)	2(2)	0.74	11%
	IgM heav	12386	94	7(3)	4(2)	1.65	37%
	Ig kappa	12148	91	8(1)	2(1)	0.28	38%
Dlat	Dihydroli	68469	90	6(3)	5(2)	0.15	12%
Igkv12-8	Immunog	12131	90	9(3)	1(1)	1.12	17%
Igkv14-10	Immunog	12813	89	4(3)	1(1)	0.27	13%
Vim	Vimentin	49220	87	8(2)	7(2)	0.14	18%
Hnrnpu	B30.2/SI	77152	86	1(1)	1(1)	0.04	2%
	Anti-hun	13085	84	5(3)	4(3)	1.53	33%
	Anti-hun	12208	84	3(3)	1(1)	0.64	15%
Ighv13-2	Immunog	11450	82	8(3)	5(3)	1.87	68%
Pspc1	Paraspec	58835	78	3(2)	3(2)	0.11	8%
Ighv1-5	Immunog	10953	77	6(2)	2(1)	0.73	34%
Gm8797	Predicted	8723	76	3(2)	3(2)	0.96	40%
	Anti-myc	11331	75	5(3)	4(3)	1.22	80%
Sfpq	Unchara	28328	73	4(1)	3(1)	0.25	15%
Igkv12-9	Immunog	12508	72	2(2)	1(1)	0.62	16%
Ighv1-9	Immunog	13037	71	6(5)	2(1)	1.02	34%
C1ra	Complem	81504	70	12(2)	5(2)	0.08	8%
	Anti-HIV	12351	68	5(4)	3(2)	0.63	18%
Igkv4-70	Anti-MO	14630	68	7(2)	4(2)	0.52	37%
	IgM heav	12410	67	11(3)	3(3)	1.08	21%
Fcgr1	High affi	45259	64	5(2)	3(1)	0.07	7%
Ighv3-3	Immunog	11404	64	2(1)	1(1)	0.30	16%
	Imunoglo	14188	63	2(1)	1(1)	0.24	12%
22100100	RIKEN cD	27089	61	20(2)	2(1)	0.12	8%
C4b	C4a ana	194437	60	3(1)	3(1)	0.02	2%
Eef1a1	Elongatio	50414	59	2(1)	2(1)	0.07	4%
Igh	Igh prote	53315	58	5(2)	3(2)	0.20	6%

Rpl13	60S ribos	24639	56	4(2)	3(1)	0.14	13%
Pdhx	Pyruvate	54250	55	1(1)	1(1)	0.06	1%
Igkv3-9	Immunog	13023	54	1(1)	1(1)	0.26	9%
Vcp	Uncharac	80669	53	2(1)	2(1)	0.04	3%
	IgA heav	12143	52	8(3)	3(2)	1.12	38%
Mtch2	Mitochor	32951	51	1(1)	1(1)	0.10	3%
Igkv14-1	Immunog	13207	51	1(1)	1(1)	0.26	11%
	Ig kappa	13357	49	3(1)	3(1)	0.26	26%
Ighv1-49	Immunog	10976	48	3(1)	2(1)	0.31	19%
Igkv11-12	Immunog	12973	46	2(1)	1(1)	0.26	13%
Igkv4-91	Anti-myc	10885	46	1(1)	1(1)	0.32	10%
	IgA heav	8933	46	7(3)	3(2)	0.94	29%
Ddx5	DEAD bo	69750	45	1(1)	1(1)	0.05	1%
Ighv1-58	Immunog	13100	44	1(1)	1(1)	0.26	12%
Ldhc	L-lactate	25448	44	4(2)	3(2)	0.28	9%
Hist2h4	Histone	11360	44	4(2)	2(1)	0.30	15%
Cct7	T-compl	55536	43	4(1)	3(1)	0.06	6%
Col3a1	Uncharac	140112	42	1(1)	1(1)	0.02	0%
Slc25a31	ADP/ATF	35521	42	3(1)	3(1)	0.09	9%
Ighv2-5	Immunog	12719	42	6(1)	3(1)	0.61	27%
Lyz1	1,4-beta-	17240	42	3(2)	2(1)	0.20	12%
Pabpc6	Polyaden	71356	40	4(2)	4(2)	0.09	6%
Osbpl3	Oxystero	101346	40	3(2)	2(1)	0.03	2%
Fyttd1	UAP56-i	35923	37	4(1)	3(1)	0.09	8%
IGHV	Immunog	12969	37	2(2)	1(1)	0.26	10%
	Anti-myc	12158	36	5(1)	1(1)	0.28	21%
Igkj1	Immunog	1447	36	2(2)	1(1)	3.07	69%
	VH regio	11299	36	3(2)	2(1)	0.30	18%
Anxa2	Annexin	21957	35	1(1)	1(1)	0.15	4%
Jup	Junction	82490	35	5(1)	4(1)	0.04	5%
Igkv4-50	Immunog	12914	35	2(1)	1(1)	0.27	11%
Igkv8-26	Immunog	13983	35	1(1)	1(1)	0.24	7%
Runx1	Runt-rel	48724	35	1(1)	1(1)	0.07	1%
Hist1h2bj	Histone	13570	34	2(1)	1(1)	0.25	7%
Atp5a1	ATP synt	54675	34	4(1)	3(1)	0.06	3%
Akap9	A-kinase	439138	34	5(1)	4(1)	0.01	1%
Suclg1	Succinat	36474	33	1(1)	1(1)	0.09	4%
Igkv17-12	Immunog	12493	33	8(3)	2(1)	0.62	34%
9530053	RIKEN cD	292361	33	3(0)	1(0)	0.01	0%
Dlst	Dihydroli	49306	33	2(1)	2(1)	0.07	4%
	Anti-HIV	13133	33	1(1)	1(1)	0.26	6%
Hnrnpm	Uncharac	86893	32	3(1)	2(1)	0.04	2%
Slc2a3	MFS dom	53957	32	1(1)	1(1)	0.06	1%

C3	Complem	187905	32	10(1)	6(1)	0.02	3%
Igkv4-54	Immunog	10344	32	1(1)	1(1)	0.34	18%
Mettl3	N6-aden	65274	32	2(2)	1(1)	0.05	1%
	IgA heav	10592	32	3(1)	3(1)	0.76	35%
Hsp90aa	Hsp90aa	66081	31	2(1)	2(1)	0.10	3%
Gstm1	Glutathic	28709	30	1(1)	1(1)	0.12	6%
Ybx2	Y-box-b	31419	30	1(1)	1(1)	0.11	5%
Brsk2	BR serine	76033	30	1(0)	1(0)	0.04	1%
Igkv15-10	Immunog	12767	29	1(1)	1(1)	0.27	13%
	Ig heavy	15504	28	1(0)	1(0)	0.22	7%
Nf1	Neurofibr	323097	26	6(1)	3(1)	0.01	0%
Prf1	Perforin	63239	25	1(0)	1(0)	0.05	1%
Prss1	Protease	26802	22	4(0)	3(0)	0.12	15%
Rbm45	Uncharac	47977	19	20(1)	2(1)	0.07	3%
